# Magnesium-based alloys with adapted interfaces for bone implants and tissue engineering

**DOI:** 10.1093/rb/rbad095

**Published:** 2023-11-01

**Authors:** Iulian Antoniac, Veronica Manescu (Paltanea), Aurora Antoniac, Gheorghe Paltanea

**Affiliations:** Faculty of Material Science and Engineering, National University of Science and Technology POLITEHNICA Bucharest, 060042 Bucharest, Romania; Academy of Romanian Scientists, 050094 Bucharest, Romania; Faculty of Material Science and Engineering, National University of Science and Technology POLITEHNICA Bucharest, 060042 Bucharest, Romania; Faculty of Electrical Engineering, National University of Science and Technology POLITEHNICA Bucharest, 060042 Bucharest, Romania; Faculty of Material Science and Engineering, National University of Science and Technology POLITEHNICA Bucharest, 060042 Bucharest, Romania; Faculty of Electrical Engineering, National University of Science and Technology POLITEHNICA Bucharest, 060042 Bucharest, Romania

**Keywords:** adapted interfaces, bone tissue engineering, fracture healing, Mg-based alloys, surface modifications

## Abstract

Magnesium and its alloys are one of the most used materials for bone implants and tissue engineering. They are characterized by numerous advantages such as biodegradability, high biocompatibility and mechanical properties with values close to the human bone. Unfortunately, the implant surface must be adequately tuned, or Mg-based alloys must be alloyed with other chemical elements due to their increased corrosion effect in physiological media. This article reviews the clinical challenges related to bone repair and regeneration, classifying bone defects and presenting some of the most used and modern therapies for bone injuries, such as Ilizarov or Masquelet techniques or stem cell treatments. The implant interface challenges are related to new bone formation and fracture healing, implant degradation and hydrogen release. A detailed analysis of mechanical properties during implant degradation is extensively described based on different literature studies that included *in vitro* and *in vivo* tests correlated with material properties’ characterization. Mg-based trauma implants such as plates and screws, intramedullary nails, Herbert screws, spine cages, rings for joint treatment and regenerative scaffolds are presented, taking into consideration their manufacturing technology, the implant geometrical dimensions and shape, the type of *in vivo* or *in vitro* studies and fracture localization. Modern technologies that modify or adapt the Mg-based implant interfaces are described by presenting the main surface microstructural modifications, physical deposition and chemical conversion coatings. The last part of the article provides some recommendations from a translational perspective, identifies the challenges associated with Mg-based implants and presents some future opportunities. This review outlines the available literature on trauma and regenerative bone implants and describes the main techniques used to control the alloy corrosion rate and the cellular environment of the implant.

## Graphical abstract

**Figure:**
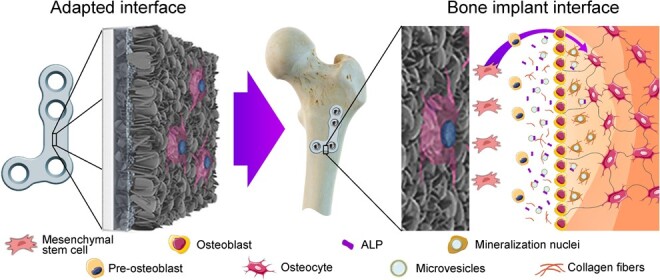

## Clinical challenges related to bone repair and regeneration

Nowadays, bone defects are a major health concern due to their prevalence. This issue results in a variety of problems, including decreased productivity, higher healthcare expenses, limited mobility and significant pain for those affected. The main causes of orthopedic fractures are motor vehicle accidents and osteoporosis. It is estimated that by 2040 a number of 316 million people will suffer from osteoporotic fractures [1]. A statistical analysis presented in [2] showed that about 3 million patients had yearly femoral shaft fractures. It is well-known that the injury mechanisms such as oblique, transverse or comminuted fractures, the complexity of the fracture site such as spine, head and limbs, and injury types are important factors that must be considered to establish a reliable treatment.

Bone regeneration is a complex physiological process that can be observed during fracture healing, and it is included in continuous bone remodeling all adult life. Bone defects can be cavity defects, for which the bone loss does not interfere with the biomechanics of the limb, or segmental defects that significantly impact bone stability and compromise the normal biomechanics’ movements [3]. Schemitsch proposed in [4] a classification of bone defects by considering that the small ones exhibited a size lower than 2 cm and a 50% cortical circumference loss. In the case of intermediate defects, the size was between 2 and 6 cm, with cortical circumference loss higher than 50%, and for the large bone defects, the size was established to be higher than 6 cm [5]. For small bone defects, fractures heal without scar tissue apparition, and new bone formed after the injury is similar to the adjacent healthy tissue. The intermediate and large bone defects can be included in the critically sized defect class, which are in the order of 2.5 cm or greater and are defined as a defect that will not heal spontaneously and requires supplementary surgical operations, although it was firstly right stabilized. Unfortunately, the absence of a standard definition may confuse the treatment of defects with sizes between 1 and 2 cm and >50% loss of the bone circumference and conflicting opinions in the patient treatment can occur. This definition must also take into consideration the anatomic defect location and the status of the soft tissue health, which can have an important influence on the healing process [4]. This process is impaired in the case of intermediate and large bone defects because a high quantity of bone must be produced for a complete skeletal reconstruction. Usually, the bone regeneration process can be described as a continuous process of bone conduction and induction, dependent on many cell types and extracellular and intracellular molecular-signaling pathways [6, 7]. Unfortunately, in some cases, due to infection, trauma, skeletal abnormalities, avascular necrosis or tumor resection, the bone cannot heal normally, and clinical approaches used to enhance bone regeneration must be applied.

**Figure 1.**
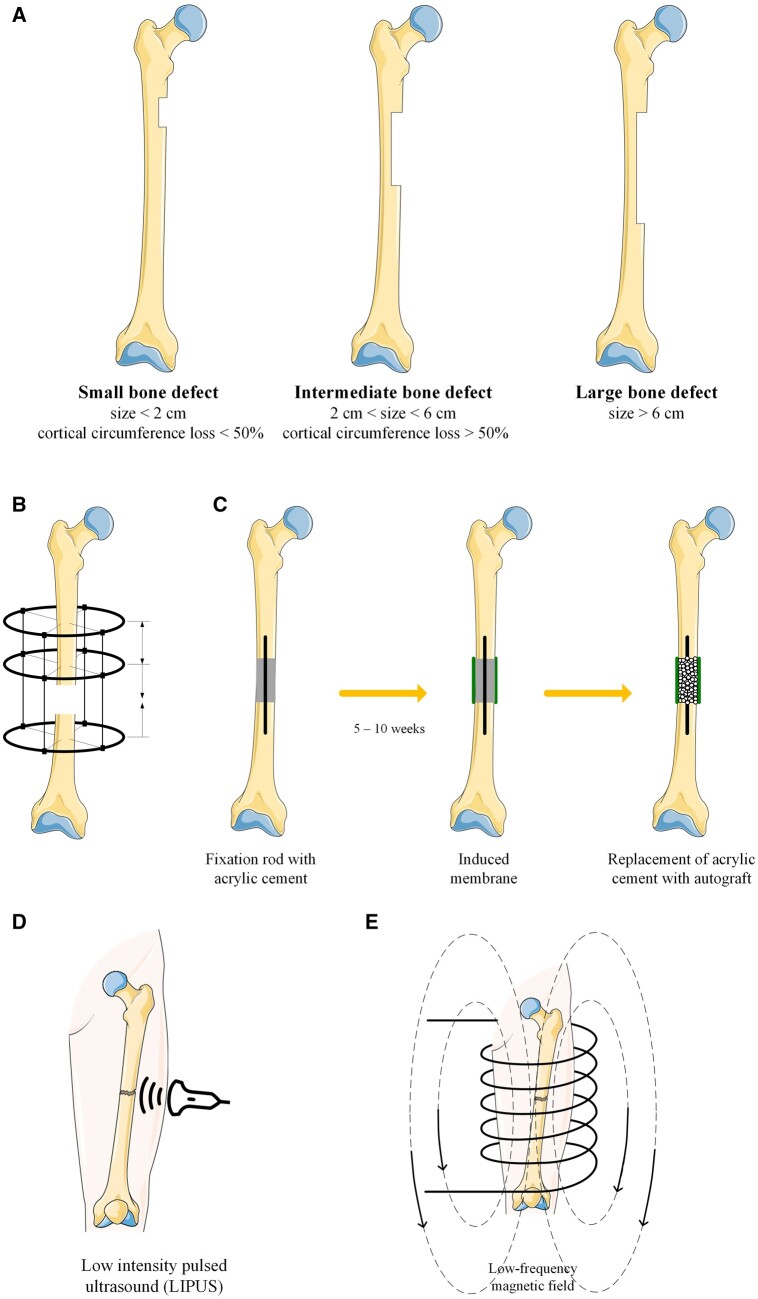
Bone defects and different treatment techniques. (**A**) Classification of bone defects; (**B**, **C**) invasive treatment strategies: Ilizarov, Masquelet; (**D**, **E**) non-invasive treatment strategies: low-intensity ultrasound therapy, pulsed electromagnetic field. The figure was generated using images assembled from Servier Medical Art, which are licensed under a creative commons attribution 3.0 unported license (https://smart.servier.com, accessed on 2 April 2023).

The applied strategies to correct bone defects are classified into non-invasive and invasive treatments. The non-invasive solutions include pulsed electromagnetic fields [8], hyperthermia [9] or low-intensity ultrasound therapy [5]. Invasive methods include surgical treatments (e.g. debridement, Ilizarov technique, Masquelet technique, osteotomy, arthroplasty for hip and knee replacement, osteogenic distraction, bone transplantation, arthroscopy and arthrodesis) that are usually applied in practice to provide a treatment for critical-size bone defects (Figure 1).

The gold standard for critical-sized defects involves using autologous bone grafts taken from the patient iliac crest. Unfortunately, many side effects can occur, and the treatment must be chosen by considering the size of the bone defect, its location and the surrounding healthy soft tissue. It was proven that the segmental defects of the femur with a size between 6 and 15 cm could heal without complications because the femur exhibits good soft tissue coverage [10]. Problems were reported in the case of much more minor defects with a size between 1 and 2 cm and a circumferential loss >50% localized in the diaphysis of the tibia, which is characterized by a limited blood supply and surrounding soft tissue [11, 12]. Other treatment solutions can include growth factors and bone-graft substitutes or allografts [13] combined with bone transport and distraction osteogenesis [14]. In the case of bone grafts, an increased risk of infections, substantial pain and incomplete defect healing are possible. Regarding the bone transport and distraction osteogenesis technique, bone regeneration is produced based on the slow development of the distracted osseous surface. The most used methods are based on external fixators and the Ilizarov technique [15], which can be supplemented by intramedullary nails that have external monorail distraction parts [16]. These techniques are characterized by different drawbacks, such as the prolonged duration of the treatment for the distraction and consolidation processes, limb shortening, deformity, high impact on joints or severe pain in some patients that require limb amputation. The positive results of the Ilizarov technique are impaired by factors such as patients older than 20 years, high bone loss and a diaphyseal defect site [16]. Lately, this technique has been combined with tissue engineering. It is noted that pure Mg and its alloys may assist distraction-induced bone regeneration. In [17], an external device made from Mg-Al-Zn (AZ31) alloy was used for distraction osteogenesis in a canine model. It was noticed that the fixation stability of the implant was similar to that provided by a stainless-steel device. The degradation products resulting from the implant deterioration were not harmful, but the efficiency of the AZ31 alloy in reducing the distraction osteogenesis treatment time was not investigated [18]. Another treatment option described in the literature consisted of hybrid devices containing an Mg-based alloy intramedullary nail used to promote bone consolidation in combination with an external fixator. It can be foreseen that the development of intramedullary nails made entirely from Mg-based alloys or coated with Mg can be very helpful to the bone formation process and reduce the distraction osteogenesis time because it upregulates the expression of different endogenous agents, which enhance the osteogenesis, angiogenesis, and neuronal regeneration [18]. Li *et al.* [19] proved that a pure Mg nail introduced into the marrow cavity and combined with distraction osteogenesis was an efficient treatment. It generated a 5-fold increase in new blood vessel formation and a 4-fold in new bone formation in comparison with the results obtained in the case of simple distraction osteogenesis (control group) in rats with a 5-mm femoral segmental defect. It was noticed that the Mg implant upregulated the calcitonin gene-related peptide (CGRP) in the new bone. This peptide-rich positive nerve fiber sustained the phosphorylation of focal adhesion kinase and simultaneously increased the expression of vascular endothelial growth factor (VEGF) A. The authors concluded that this link between endothelial cells and sensory nerve could be a principal mechanism explaining the beneficial effect of Mg implants when combined with distraction osteogenesis for critical-size defects, resulting in reduced treatment time.

Another solution surgeons propose is the reamer-irrigator-aspirator (RIA) method, which consists of cell collection from the femoral canal after reaming. This type of product exhibits a high similarity with the cells collected from the iliac crest [20, 21]. It was concluded that the RIA technique has a benefic effect on the mesenchymal stem cells’ (MSCs) osteogenic differentiation in comparison with the classical methods such as cancellous bone harvesting and marrow aspiration [20, 22]. The revascularization process appears in the first 3 months of reaming, followed by bone thickness improvement that occurs after 14 months [23]. Like the other described techniques, RIA exhibited promising results for cavity defects, but an osteosynthesis supplementation is necessary in the case of segmental defects.

One of the most used techniques in long bone defect treatment is the Masquelet method, which involves growing an induced membrane onto an acrylic cement rod implanted to cover a lengthy bone hole. When the membrane has been formed, the cemented rod is removed, and the space is then filled with allogenic or autologous cancellous bone [24]. Some studies put in evidence the fact that based on this technique, the revascularization process occurred after 30 days, but its level was drastically diminished after 90 days [25]. The 30-day-old membranes exhibited an increase of VEGF, collagen 1 and interleukin 6 by comparing it with the 60-day-old samples that were characterized by <40% of the 30 days levels [25–27]. Although the Masquelet technique proved efficient in some cases, due to the prolonged and complicated reconstruction process, its outcome is not predictable, and more research must be conducted to optimize this method properly [28].

A combined treatment based on stem cell therapy and tissue engineering has been developed. The most used stem cells are the multipotent MSCs that result from postnatal adult stem cells, compared to the embryonic stem cells (ESCs) or induced pluripotent stem cells (IPSCs) because these cell lines have practical limitations regarding cytogenetic regulation of teratoma development. Also, different ethical issues, immune system response for ESCs, or genetic reprogramming in the case of IPSCs must be considered [29–31]. The main sources for stem cells involved in clinical research are bone marrow and adipose tissue [32]. These cell lines can be combined with different types of scaffolds to restore bone defects. Also, stem cells can differentiate into osteoblasts. Still, this strategy also has some limitations, such as serum-free culture media to avoid infection and contamination risks, the *ex vivo* multiplication process for cells for more weeks that can generate genomic unpredictability in culture, and cell recruitment, manipulation and grafting must be done in one-step operation [33].

One of the most important challenges of bone defect treatment consists of vascularization and angiogenesis because to maintain bone viability, a highly vascularized medium around it is necessary, facilitating the transfer of oxygen and nutrients [34, 35]. Another challenge identified in the literature [36, 37] is the bone induction that is directly linked to the differentiation process of the cells into osteoblasts under the surrounding microenvironment action. This phenomenon is essential in bone regeneration because it determines the failure or success of reconstructive and regenerative treatments. In the case of implant use, osteoconduction becomes of interest due to the existence or absence of vascular and bone cells’ attachment, proliferation, and migration in the implant pores [5]. Osseointegration can be considered another important challenge in bone defect treatment, and it can be described as a non-fibrous connective tissue formation that acts as an interface between implant and bone [38, 39]. Clusters made from osteoblasts on the prosthesis or scaffold surface occur, and new bone formation is facilitated [5].

Biomedical materials can be used for bone implant manufacture to help the healing of the bone. Three main requirements are applied when a material is selected for bone treatment. Firstly, it must have excellent mechanical properties with values approximately equal to those of healthy bone to avoid the so-called “stress shielding” effect; secondly, its biocompatibility must be increased to hinder the immune system reaction, and finally, in the case of temporary implants the material must have good corrosion behavior to permit a controllable degradation process followed by bone tissue regeneration. Regarding permanent implants, there are many primary reasons for surgical removal, such as foreign body reactions, permanent nail paths and seldom metallic ion emission. This type of implant is indicated to be removed if complications appear at a certain time because, in some cases, difficulties were met a long time later. Foreign body reaction occurs because the implantation process generates tissue injuries around the foreign object, starting an inflammatory process. After a variable time comprised between weeks and months, the inflammatory process evolves through a fibrotic response that isolates and envelops the permanent implant [40]. The formed fibrous capsule represents an important disadvantage for the implant and can be linked to non-specific protein absorption on the implant surface, leading to implant failure or complications in some cases. Filip *et al.* [41] summarized the following drawbacks related to metallic permanent implants as a function of material: for stainless steel, allergic reaction and stress shielding effects were reported since the cobalt-chromium-based alloys were related to early implant failure and titanium-based alloys generated bone resorption and allergic responses. The permanent nail path is an important concern, which refers to the fact that when a permanent implant is introduced inside the human body, local modifications placed in the bone metaphysis, such as permanent drilled tunnels or channels, occur. They can be difficult to treat when a later implant insertion is needed because the bone area in the affected zone (HAZ) modifies its hardness, so this induced mechanical anisotropy can push the implant into a non-anatomical position. Usually, permanent inert metallic implants are used to fix musculoskeletal injuries. Unfortunately, they are characterized by limitations, such as the stress shielding effect, which appears due to Young’s modulus value mismatch between human bones and implant, leading to secondary fractures with poor or slow healing. Also, the metallic ion emission is considered another concern with these materials because bone osteolysis can be induced, and implant mobilization occurs. Metallosis is an affection of the human tissue characterized by the deposition and accumulation of metal debris in the body’s soft tissues associated with metallic implants. In addition, local and systemic symptoms can characterize it through a chronic inflammatory response. In these rare cases, implant instability appears because fractures do not heal and lead to high mechanical stress sustained only by the implant. So, as a direct consequence, implant failure due to cyclic stress can be expected. Removal surgery is necessary in all these cases [42, 43]. Another material extensively used in orthopedy is biodegradable polymers with adequate mechanical properties with values close to those of cancellous bone, high biocompatibility, and exhibit biodegradability properties [8, 44]. The main drawbacks of polymeric materials can be considered the long-term foreign body responses due to their byproducts and the diminution of the mechanical strength, which decreases in time due to the biodegradation process. The most commonly used bone substitutes are synthetic ceramics based on calcium phosphates such as hydroxyapatite (HAp), beta-tricalcium phosphate (β-TCP), other compounds and bioactive glasses (BGs). They have important disadvantages, such as reduced mechanical properties and the impossibility of use as implants in load-bearing zones due to their brittle nature [45]. One of the most modern solutions to treat bone defects involves Mg-based alloys. Magnesium is used to manufacture fixation implants such as nails, plates and screws, the main stabilization devices in fracture management [46, 47]. Supplementary, porous Mg alloy scaffolds can be developed to enhance bone regeneration in non-union defects [48]. Although Mg-based alloys have become an important and actual research topic, their main drawback, which consists of an accelerated corrosion process that must be controlled using different solutions, such as alloying with other inorganic compounds [49] or using different surface treatments [47, 50, 51] must be carefully addressed.

In this review article, we focus our attention on Mg-based alloy implants with adapted interfaces for fracture healing and hard tissue regeneration. We investigate the main interface challenges of Mg implants by considering their effect on new bone formation, fracture and osseous defect treatments, the degradation phenomena and hydrogen release, and the mechanical properties variation during the degradation process. Trauma implants and scaffolds from biodegradable magnesium alloys section presents the design, the most important examples of implant types found in the literature, and the manufacturing technologies. Adapted interface between Mg alloys and bone section is devoted to a comprehensive analysis of methods for obtaining adapted interfaces between Mg alloys and bone, underlining the effect of surface modifications and coatings for corrosion control, mechanical property improvement and cellular environment enhancement. Our published results are combined with other authors’ findings to sustain and present the most used methods for surface treatments of Mg-based implants. The last part of the article gives some recommendations from a translational perspective, challenges and opportunities of this topic.

## Interface challenges of Mg-based implants

This section will discuss crucial interface challenges regarding implants made from Mg-based alloys. We will consider the positive impact of magnesium on fracture healing and new bone formation, as well as the negative effects associated with hydrogen evolution during the alloy degradation process and changes in mechanical properties under different conditions. The main advantages of the Mg-based implants can be easily summarized as follows: osteogenesis and high biocompatibility [52, 53], biodegradability and possibility to avoid implant removal surgery [47, 54], increased damping capacity [51], dimensional stability [55] and good mechanical properties [47, 50]. As we have underlined in Clinical challenges related to bone repair and regeneration section, the major drawback of Mg alloys is their low corrosion resistance in physiological media [47, 56], which promotes a fast decrease of the mechanical properties resulting in the early failure of implants before the completion of the tissue-healing process [57]. Also, the hydrogen evolution that represents the main cathodic reaction, which occurs simultaneously with the Mg-based alloy corrosion process, may significantly impair bone healing [58, 59]. Magnesium has an electrochemical potential of −2.3 V versus the standard hydrogen electrode and is considered to be highly prone to corrosion in aqueous media. The resulting oxide/hydroxide layer on the implant surface does not protect in a high amount the material. The chloride ions in the human body transform the hydroxide layer into a magnesium chloride compound. The dissolution of the protective layer accelerates the material corrosion process, and due to hydrogen gas emission, subcutaneous bubbles occur and create, in some cases, a separation zone between implant and tissue [50, 51]. A physiological limit for the hydrogen evolution rate was established to values lower than 0.01 ml/cm^2^/day [47]. Another important disadvantage of Mg-based alloys is their low elastic modulus, which can be beneficial in stress shielding effect avoidance, but in the case of load-bearing applications, the implants must be engineered to hinder mechanical deformation [60].

### Fracture healing and new bone formation

In this section, we summarize some literature studies that describe examples of fracture healing and new bone formation in different animal models. We underline the importance of magnesium ions in osteogenic differentiation by comprehensively analyzing different coated or uncoated Mg-based implants. We discuss the limitations of some studies that used uncoated implants and point out the importance and beneficial effect on bone healing of surface coating by providing the authors’ conclusions extracted from the literature.

It is well-known that bone can be considered a dynamic tissue with precisely coordinated resorption combined with an adequate synthesis of osseous tissue [61]. Regarding the inorganic compound Mg, 67% is stored in bone, and 30% can be exchanged through the bone crystal surface [56]. Some studies show that magnesium ions (Mg^2+^) sustain the total rate of calcium phosphate crystallization, determining the HAp growth [62]. Supplementary, Mg^2+^ ions proved to induce osteoblast differentiation [63] since a study [64] performed on Sprague Dawley (SD) rats with a weight between 150 and 175 g showed that an Mg deficiency between 0.04% and 10% of the nutrient requirement of about 0.82 g/kg determines the increase of osteoclast production [65], diminishing the bone regeneration process concomitantly and being associated with an increased release of a neuropeptide denoted as substance P and tumor necrosis factor TNF-α. Consequently, bone mass decreased, producing proinflammatory cytokines [66, 67].

In a study performed by Vormann [68], an estimated average requirement of 300 mg/day for women and, in the case of men, a value of about 350 mg/day, are considered optimal for Mg intake following the recommended dietary allowances.

The osteogenic differentiation of the stem cells is governed by the MAPK/ERK pathway, which Mg^2+^ ions can selectively activate by inducing cyclic adenosine monophosphate (cAMP)-responsive element binding protein 1 (CREB1) phosphorylation through Mg^2+^ channel magnesium transporter 1 (MAGT1) [69, 70]. MAGT1 influences the osteoinductive role of Mg by controlling the influx of Mg^2+^ and promoting bone marrow mesenchymal stem cell (BMSCs) differentiation directly into osteoblasts through a transmembrane transport process [71]. Supplementary, the magnesium ions exhibit a beneficial influence on the expression of intracellular osteogenic signaling molecules such as osteocalcin (OCN), type-I collagen (COL1), alkaline phosphatase (ALP) and runt-related transcription factor 2 (RUNX2) [72].

**Figure 2.**
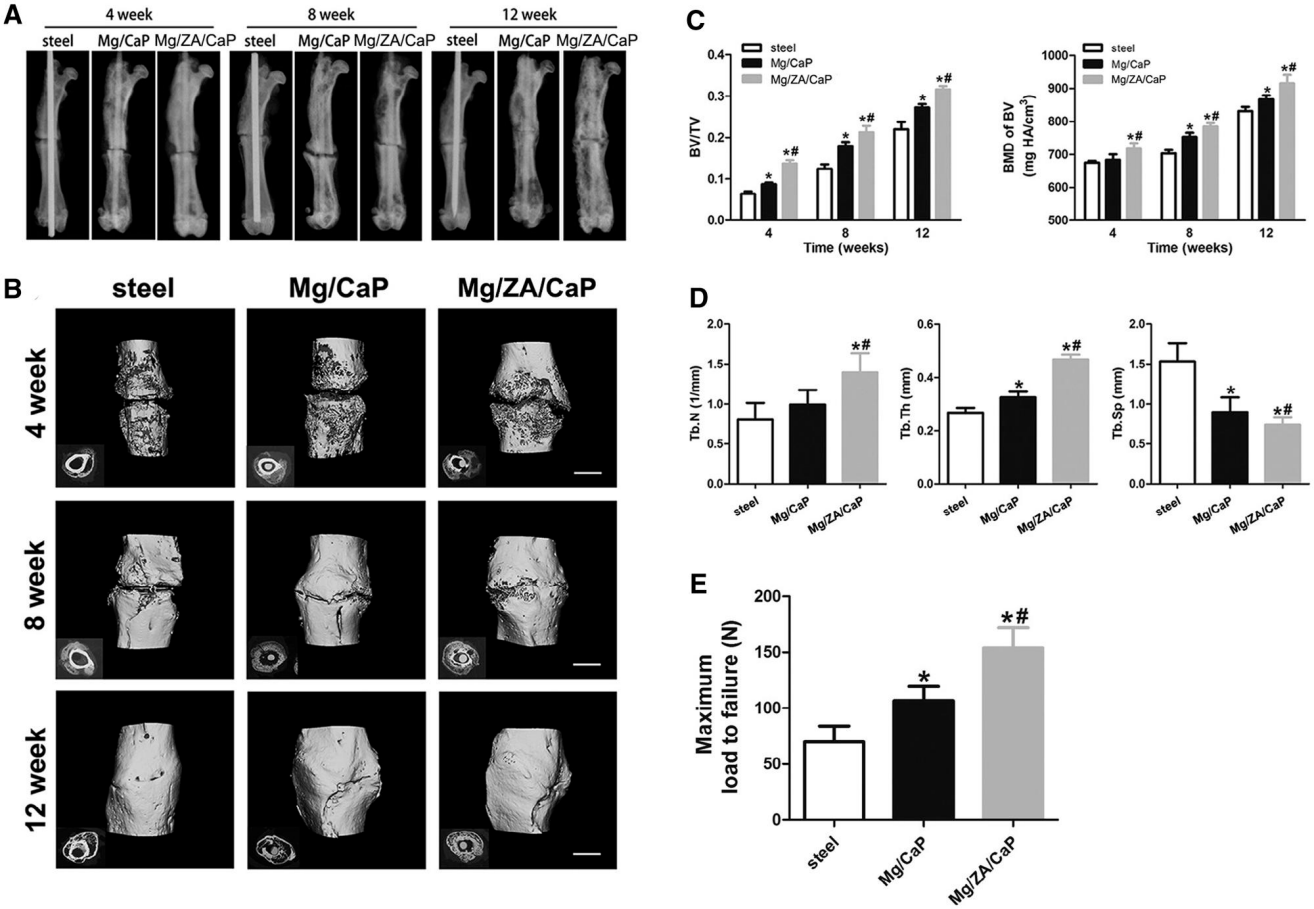
The fracture healing effect of the control (steel) and Mg-based (Mg/CaP and Mg/ZA/CaP) implants. (**A**) X-ray investigations; (**B**) micro-CT scans; (**C**) bone volume fraction (BV/TV) and mineral density (BMD) in the three investigated materials between 4 and 12 weeks after the surgery; (**D**) trabecular number (Tb.N), thickness (Tb.Th) and spacing (Tb.Sp) in the case of Mg/ZA/CaP implants at 12 weeks; (**E**) maximum load to failure of the fractured bone for Mg/ZA/CaP pins at 12 weeks (^*^P < 0.05 vs. stainless steel control group; ^*#^*P* < 0.05 vs. Mg/CaP group) [73]. Reprinted from [73] Copyright (2023), with permission from Elsevier.

Li *et al.* [73] analyzed the dual modulation of bone formation and resorption for zoledronic acid (ZA)-loaded magnesium alloy implants in the case of osteoporotic fractures. They developed Mg-Nd-Zn-Zr (JDBM) nails coated with polylactic acid (PLA)/brushite (CaP) and loaded with ZA and implanted in osteoporotic SD rats that were subjected to a mid-shaft femoral transverse osteotomy. It was concluded that the amount and density of newly generated trabeculae and calluses of the tested magnesium alloy group were better than those of the control group at 8 and 12 weeks after the surgery (Figure 2).

Kong *et al.* [74] investigated the *in vivo* biodegradation and biocompatibility of Mg-Nd-Zn-Zr alloy screws by creating a femoral condyle fracture model in goats. The implants were coated with brushite or PLA. The histological images showed that the uncoated and brushite-coated samples exhibited superior capabilities for new bone formation compared to those coated with PLA. The osteogenic factors such as bone morphogenetic protein, ALP and OCN were characterized by decreased values in the peri-implant callus area for the PLA-coated screws. It was noticed that the thickness of the brushite-coated samples could be adjusted according to the necessity of bone formation. Zhang *et al.* [69] developed pins that contain an uncoated pure magnesium rod inserted in a hollow intramedullary nail made from stainless steel and implanted into the distal femur of rats. New bone formation was observed at peripheral cortical sites concomitantly with increased neuronal CGRP-α in the ipsilateral dorsal root ganglia and cortex of the femur. The cAMP-signaling pathway was activated by the CGRP receptor and phosphorylated the transcription factor CREB1. This led to the activation of the transcription factors SP7 (*Osterix—*Osx) and RUNX2, two additional transcription factors that are essential for osteogenesis. It was observed that these innovative implants promoted fracture healing due to the benefic effect of Mg^2+^ ions exhibiting callus formation, intramembranous and endochondral ossification. The authors concluded that the development of this study would consist of magnesium rod manufacture designed to provide osteogenic effect and mechanical support to repair the long-bone fracture and possibly include some innovative implant coatings. Han *et al.* [75] made *in vivo* and *in vitro* analyses on the degradation phenomenon of an uncoated pure Mg screw, which was inserted in a femoral intercondylar fractured rabbit model. After the *in vitro* studies, they showed that the implant favored bone cell viability, and increased values for ALP, osteopontin (OPN) and RUNX2 were reported. Regarding the *in vivo* analysis, increased bone volume and good mineral density were observed at different moments, such as 4, 8, 16 and 24 weeks. The fracture gap was almost closed, and the authors concluded that pure Mg screws are adequate implants for fracture healing. The limitation of this study can be considered the fact that pure magnesium suffered a rapid corrosion process, and although the authors considered that it was adequate for animal models in the case of a large body, a decrease in the mechanical properties can be foreseen, so measures for corrosion rate reduction must be applied. Schaller *et al.* [76] investigated the bone remodeling process and fracture healing by comparing the results obtained in the case of two trauma implants. These were plates and screws made from uncoated Mg-Y-Nd-Zr (WE43) and polylactide-co-glycolide (PLGA). The *in vivo* studies were performed on Yucatan miniature pigs. The osteotomies were done in the animals’ midface in the supra-orbital rim and zygoma. It was concluded, in accordance with the radiological and clinical follow-up, that all the fractures healed well. A complete bone union was observed, and no negative influence of hydrogen pocket was seen. Also, it was noticed that Mg, in comparison with PLGA, is an innovative material that permits the manufacture of implants with reduced size and good mechanical properties if additional solutions are considered to control its degradation.

Dong *et al.* [77] prepared Mg-based scaffolds using additive manufacturing (AM) technology. The scaffolds were coated with magnesium fluoride (MgF_2_) and MgF_2_-CaP to reduce their corrosion rate and increase biocompatibility. Direct culture of MC3T3-E1 murine preosteoblasts was seeded on scaffold extracts and showed good bone formation capabilities. Xie *et al.* [78] fabricated three-dimensional (3D) porous uncoated scaffolds from Mg-Nd-Zn-Zr (JDBM). The scaffolds showed good cytocompatibility in immature osteoblasts MC3T3-E1 and macrophage RAW267.4 mouse cells and high osteoconductivity. Based on histological analysis, blood tests, and Mg^2+^ deposition detection outcomes, the 3D-printed JDBM scaffolds demonstrated high *in vivo* biocompatibility and prevented methicillin-resistant *Staphylococcus aureus* (MRSA)-induced implant-related infection in a rabbit model with a distal femoral defect. It was observed that because the implants were uncoated, a high Mg^2+^ ion concentration was reported in the first 6 days. One can estimate that this release was also followed by hydrogen gas emissions that can harm healthy tissue. Augustin *et al.* [79] manufactured different pore-sized scaffolds (400 and 500 μm) from Mg-Li-Al-RE (LAE442) coated with MgF_2_. This type of alloy proved good results regarding the bone formation process near the implant. It was noticed that both scaffold geometries showed bone ingrowth. In the 500 μm pore size configuration case, improved osseointegration was obtained. The newly formed bone was also found in the central scaffold area due to a larger bone–scaffold interface. Wang *et al.* [80] developed JDBM alloy scaffolds coated with brushite with main spherical pores (400–450 μm) interconnected by smaller pores (150–250 μm). This particular geometry enhanced *in vitro* osteogenesis and mineralization in BMSCs cells. The *in vivo* studies were performed on SD rats using a femoral condyle defect with a diameter of 3 mm and rabbits based on a radius longitudinal incision with a length of 20 mm. It was concluded that the scaffolds enhanced osteogenesis and angiogenesis, and the large bone defects in rats and rabbits were perfectly repaired.

Osteogenesis and angiogenesis can be improved using bioactive coatings on Mg-based alloys that reduce their corrosion process and provide additional chemical elements useful in bone remodeling. Some studies presented in this section put in evidence the beneficial effect of uncoated Mg-based alloys due to an increased Mg^2+^ ion emission, which can help the upregulation of the MAPK/ERK pathway. However, they also underline major drawbacks such as decreased mechanical properties and hydrogen gas emissions. In the cases of rapid osteogenesis, uncoated Mg-based implants can be a good solution due to the regenerative effect of Mg^2+^ ions, but instead, for some orthopedic applications, in which time extended mechanical support is required, the coating of the Mg-based alloys is recommended. On the other hand, another option is given by the coating of Mg-based alloys with calcium phosphates containing Mg^2+^ ions [81–86]. A general conclusion of this section is that the surface of the implants must be carefully adapted in accordance with the medical application and the expected results.

Table 1 presented some *in vivo* studies that evidence the properties of new bone formation and fracture healing in the case of different coated and uncoated Mg-based alloy implants [69–86, 90].

**Table 1.** *In vivo* studies performed to underline the new bone formation and fracture healing properties

Implant material	Implant shape	Animal model				Fracture model	Remarks	Ref.
		Species	Age	Body weight	Follow-up			
Mg-Y-Nd-heavy REs (modified WE43 based on ASTM B80) coated with magnesium phosphate (Mg_3_(PO)_4_) with some traces of yttrium and neodymium	Rivet-screw	Minipig	30–36 months	53 (±7) kg	12 and 24 weeks	Mandibular fracture	The bone tissue regeneration exhibited a different pattern because of bone growth over the head of some rivet screws. Due to surface coating, the degradation process was slow, good bone density was achieved, and no side effects were reported	[87]
Mg-Y-Nd-heavy REs (modified WE43 based on ASTM B80) with a plasmaelectrolytic (PEO) coating	Rectangular plates of 60 × 6.0 ×1.50 mm^3^	Miniature pigs	30–36 months	53 (± 7) kg	4, 8, 12 and 24 weeks	Midface of the minipig	The PEO coating did not prevent corrosion, but it was very effective in delaying the gas release and enhancing the calcium phosphate apparition at the implant surface. Good bone formation capabilities were put into evidence	[88]
Mg-Nd-Zn-Zr (JDBM) coated with (PLA) or brushite	Screw	Goats	Mature	–	6 months	Femoral condyle fracture	The uncoated and brushite-coated samples exhibited superior capabilities for new bone formation compared to those coated with PLA	[74]
Uncoated Mg-Y-Nd-Zr (WE43)	Plate and screw	Yucatan miniature pigs	24 months	80 ± 10 kg	9 months	Supra-orbital rim and zygoma	A complete bone union was observed, and no significant negative influence of hydrogen pocket was seen, but coating for the alloy was recommended	[76]
Uncoated Mg-Y-Zn	Screw	Beagle dogs	1 year	10 kg	12 weeks	Crank-shaped tibial fracture	It was concluded that bone fractures at load-bearing zones could be fixed with WE43 screws, but future investigations are needed for search coatings that can enhance the biocompatibility and corrosion resistance of the screws	[71]
Mg-Zn-Gd coated with Ca-P	Membrane	New Zealand White (NZW) rabbits	16–20 weeks	3–3.5 kg	3 months	Critical-sized calvarial defect	The coated implants were characterized by denser and more mature mineralized matrix deposited in the defect compared to uncoated counterparts. It was concluded that the Ca-P coating improved bone remodeling and new bone formation	[89]
Uncoated pure Mg	Screw	NZW rabbits	Skeletally mature	3 ± 0.5 kg	24 weeks	Femoral intercondylar fracture	The fracture gap was almost closed, and the authors concluded that pure Mg screws are adequate implants for small fracture healing, but in the case of critical-size defects, different screw coatings must be used to provide sufficient mechanical support	[75]
Mg-Nd-Zn-Zr (JDBM) coated with (PLA)/brushite and loaded with ZA	Nail	SD rats	6 months	–	12 weeks	Femoral shaft fracture	The amount and density of newly generated trabeculae and callus of the magnesium alloy group tested were better than those of the control group	[73]
Uncoated pure Mg	Needle	SD rats	6 months	–	12 weeks	Femoral shaft fracture	New bone formation was observed at peripheral cortical sites concomitantly with increased neuronal CGRF-α in the ipsilateral dorsal root ganglia and cortex of the femur. The authors suggested future improvements in the use of coated needles	[69]
Uncoated Mg-Nd-Zn-Zr	Nail	SD rats	–	250–400 g	8 weeks	Fracture of tibia	There were noticed excellent fracture healing results in comparison with the control group (Ti screws) due to increased osteogenic properties of Mg^2+^ ions	[90]
Mg-Nd-Zn-Zr (JDBM) coated with coated with brushite	Scaffold	SD rats/rabbits	–	Rat unspecified Rabbit 2.5 kg	4 and 8 weeks for rats 6, 12 and 18 weeks for rabbits	Femoral condyle defect with a diameter of 3 mm for rats and a radius longitudinal incision with a length of 20 mm	The scaffolds enhanced osteogenesis and angiogenesis, and the large bone defects in rats and rabbits were perfectly repaired	[80]
Mg-Li-Al-RE (LAE442) coated with MgF_2_	Scaffold	Zika rabbits	Skeletally mature	3.9 kg	6, 12, 24 and 36 weeks	Osteotomy placed in the greater trochanter	For 500 μm pore size configuration, improved osseointegration was obtained. This process was lower than in the case of β-TCP control scaffolds	[79]
Uncoated pure Mg	Scaffold	NZW rabbits	Skeletally mature	3.87 kg	8 and 16 weeks	Lateral epicondyle	Mg scaffolds with larger pore sizes stimulate osteoblastic activity and upregulate the expression of collagen type 1 and OPN, resulting in increased bone mass and more mature bone formation at the implant site	[91]

### Degradation process for Mg-based implants and scaffolds

In this section, we focus our attention on Mg-based implants and scaffolds degradation in different physiological media. We chose in our analysis different literature studies, which put in evidence the importance of the medium chemical composition in the case of *in vitro* studies, the shape and place inside the animal body in which will be inserted the implant, because the water content is an important aspect that must be considered, and the presence or the absence of the coatings. The examples detailed in this section consider the corrosion behavior, mass loss during degradation phenomenon, and hydrogen evolution and its side effects for the *in vivo* studies. The importance of the implant interface will be discussed at the section end.

**Figure 3.**
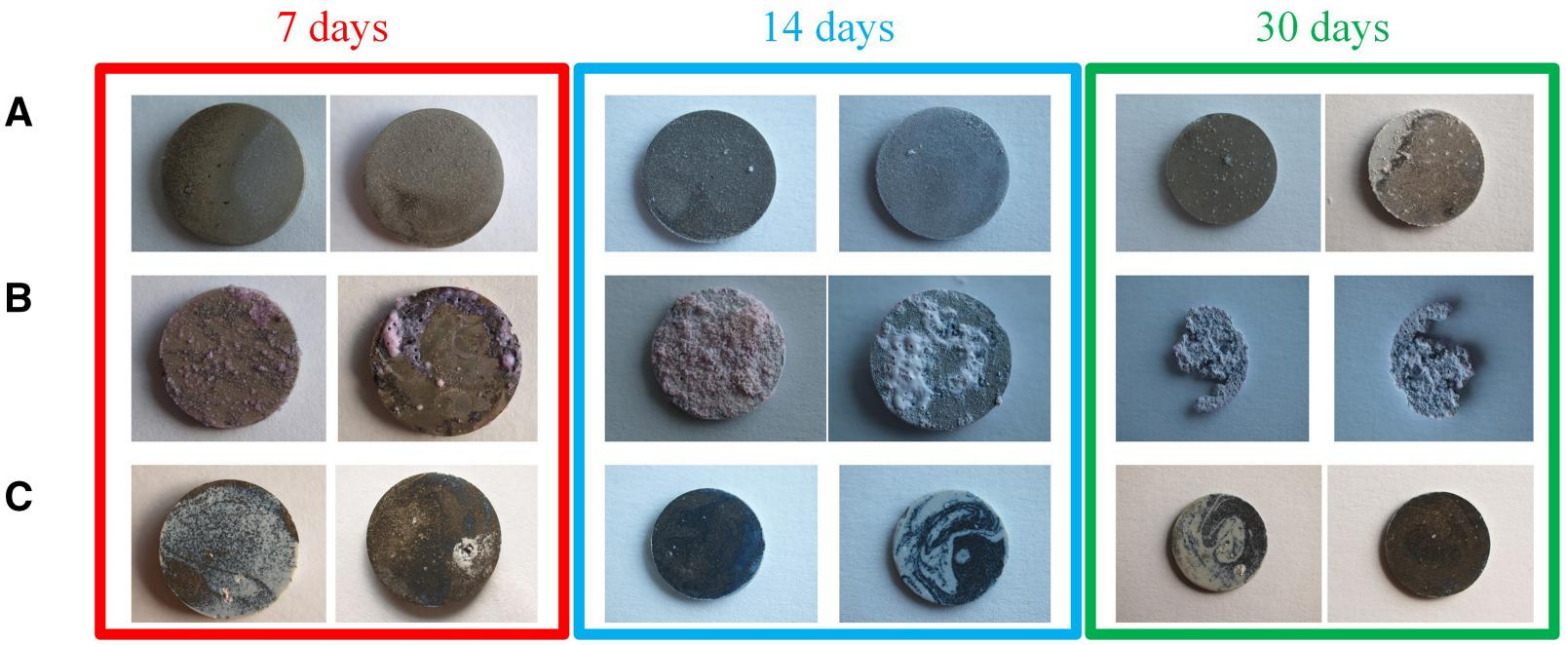
The surface macro-morphology of uncoated Mg-Ca alloys after 7-, 14-, and 30-day immersion in (**A**) SBF, (**B**) HBSS, (**C**) DMEM + 10% FBS.

As mentioned previously, the high-speed degradation of the Mg-based implants is a significant drawback that must be addressed. Different environmental factors strongly influence this process. The physiological medium is usually highly corrosive for Mg because it contains chloride ions. Supplementary, other factors such as organic buffering molecules, inorganic ions, oxygen molecules and mechanical stress must be considered [47]. Figure 3 presents the surface macro-morphologies of uncoated Mg-Ca alloys after 7, 14 and 30 days of immersion in different simulated fluids such as phosphate-buffered saline solution, Hanks’ balanced salt solution (HBSS) and Dulbecco’s modified Eagle medium (DMEM) and 10% fetal bovine serum (FBS).

Xin *et al.* [92] proved that the buffering system inside the human body based on the ${\text{HCO}}_{3}^{-}$/CO_2_ complex determines an accelerated corrosion process of Mg-based materials by generating the MgCO_3_ precipitation and dissolving the OH^−^ ions. In order to put in evidence the mechanical stress influence on the corrosion rate, Gu *et al.* [93] applied compression loads or cyclic solicitations and saw that the corrosion process of uncoated Mg-Y-RE-Zr (WE43) alloy increased. Also, the shape of the implant is very important for the degradation mechanism. Chaya *et al.* [94] observed that a higher corrosion rate was achieved for uncoated Mg-based alloy plates in the case of the ulna fracture model than in the case of screw implants. A viable explanation for this observation can be based on the fact that plates are surrounded by muscles that contain a higher percentage of blood flow and water, in comparison with screws, which are implanted in the bone. Similar differences were established in the case of Mg screw shafts and heads [95]. Wang [90] observed that an uncoated 99.93% Mg-based alloy implant used for long-term fracture treatment dissolved quickly and led to implant fracture. On the contrary, in the case of coated samples, the shape and mechanical integrity of the implant were maintained for a sufficiently long time to ensure fracture healing. Chou *et al.* [96] investigated the degradation behavior of the uncoated Mg-Y-Zn-Zr-Ca (WZ42) pin implanted to treat an osteotomy placed in a rat femur over a 14-week follow-up. It was noticed that corrosion was a gradual process observable at 2 weeks, followed by a high-stress point apparition near the fracture sites, and ended with pin fracture. New bone formation and osteoblast activity were put in evidence at 14 weeks, proving that WZ42 alloy is adequate for fracture treatments. Marukawa *et al.* [71] analyzed the degradation process of uncoated Mg-Y-RE-Zr (WE43) magnesium alloy screws compared to those made from biodegradable polymers. At the end of the fracture healing, they showed that the Mg implants did not degrade significantly since the polymeric screws were broken or severely deformed. It was concluded that the WE43 is a suitable alloy for fracture management. Regarding hydrogen generation, Chou *et al.* [96] observed a higher quantity of gas in the early stage of fracture healing, followed by reduced hydrogen emission as the fracture closed. In [90], during the middle stage of the fracture treatment, subcutaneous emphysema occurred as a consequence of the hydrogen presence. Kong *et al.* [74] noticed that during the degradation process of Mg-Nd-Zn-Zr alloy screws, a high quantity of hydrogen gas was measured in the case of uncoated samples during the early fracture stage. The hydrogen pockets were missing for coated and control implants in the middle stage of fracture healing. At the treatment end, the hydrogen was eliminated from the animal bodies for the uncoated samples, and no secondary medical effects were reported.

Dong *et al.* [77] analyzed Mg-based scaffolds’ *in vitro* degradation behavior. They investigated bare, MgF_2_-coated, and MgF_2_-CaP-coated scaffolds and concluded that the first ones lost their structural integrity after just 1 day of immersion in revised simulated body fluid (r-SBF) since the other two categories maintained well their mechanical properties. When the scaffolds were inspected on day 7, it was observed that corrosion products were present on the coated samples’ surface. Xie *et al.* [78] prepared uncoated Mg-Nd-Zn-Zr scaffolds using the selective laser melting (SLM) method. The *in vitro* degradation was performed in SBF solution at 23°C for 7 days. The degradation rate of the material was determined at a value of 0.039 ± 0.003 g/day, and it was concluded that the hydrogen release decreased directly proportional to the immersion time of the samples. The degradation process of two-pore-sized scaffolds from Mg-Li-Al-RE (LAE442) coated with MgF_2_ was investigated in [79]. The study was performed *in vivo* on 60 Zika rabbits, using surgical defects placed in the greater trochanter. The animals were euthanized at the end of each observation time (6, 12, 24 and 36 weeks). All the scaffolds showed uniform degradation behavior and a progressive volume loss. The degradation process was followed by a low increase in the surface area until week 24 for the 500 μm and week 36 for the 400 μm pore sizes. Regarding hydrogen, emission gas was detected outside and inside the scaffolds. The amount of gas was continuously reduced for the 500 μm pore size implant, and only small bubbles were found in the scaffold structure at week 36. In the case of 400 μm pore size, a higher gas quantity was detected in week 24, and it did not decrease until week 36. Cheng *et al.* [91] developed uncoated open-porous pure Mg scaffolds using the titanium wire space holder (TWSH) method. Based on this technology, the pore size and the porosity can be controlled, and the implant’s mechanical properties can be adapted well to the defect characteristics. Two pore-sized scaffolds (250 and 400 μm) were manufactured, and the *in vitro* corrosion was investigated by soaking the samples in solution. It was noticed that the 400 μm pore size implant degrades faster, and a corrosion rate of 1.53 ± 0.15 mm/year was computed since, in the case of the 250 μm pore size scaffold, a value of 1.31 ± 0.11 mm/year was reached. Xue *et al.* [97] manufactured based on 3D weaving Mg wires with controlled microstructure and chemical composition method scaffolds. The implants were coated with PLA in order to reduce their degradation process. To analyze this phenomenon, HBSS was chosen, and the samples were introduced in this physiological medium for 28 days. Using experimental measurements for the solution pH, it was concluded that the PLA coating decreased the degradation process of the implants in the early stage of the analysis. On the other hand, later, it was noticed that the uncoated samples’ pH increased more slowly, showing that the corrosion process was stabilized through the apparition of a protective film formed on the implant surface. The pH of coated scaffolds increased at a high rate, being at the end of the experiment almost equal to that obtained in the case of uncoated samples. It can be concluded that the PLA coating was deteriorated by the hydrogen evolution resulting from the Mg wire degradation.

The high-speed degradation of implant and hydrogen generation drawbacks are trying to be solved firstly by alloying pure Mg with alloying elements that can control the alloy microstructure by the grain size and the phase distribution, which strongly affect these properties [47]. Other methods used are the thermal treatments applied to dissolve the secondary phases.

Another proposed solution consists of surface treatments by applying an adequate coating or forming a passive layer onto Mg-based alloys to maintain the hydrogen emission in the biological range and modulate the corrosion rate and the speed of biodegradation. As a main conclusion of this section, we can notice the importance of controlling the degradation speed of Mg-based implants or scaffolds through different methods because the interface quality influences in a high amount the success of the treatment by reducing the side effects associated with unwanted hydrogen emissions. Supplementary, we can conclude that if we opt to use coatings, they must be highly biocompatible and biodegradable but with a much slower degradation rate in comparison with uncoated Mg-based alloys to decrease the corrosion and degradation step and reduce the by-products emission.

### Mechanical properties during the degradation process

We provide many examples that present the mechanical behavior of different Mg-based alloys used for implant or scaffold manufacture in the presence of physiological media in this section. We underline the importance of controlling the alloy degradation rate to maintain the implant’s mechanical integrity. Some differences regarding the interface of the implant are presented through research that sustained the importance of coating in reducing corrosion and mass loss by measuring optimal values for mechanical properties in comparison with uncoated alloys, which degrade faster. The *in vivo* studies presented in this section show a different behavior of the Mg-based alloys compared to *in vitro* investigations and finite element model simulations.

The orthopedic implants must exhibit good mechanical properties with values close to those of the human bone. The mass density of the Mg is equal to 1.74 g/cm^3^, and its Young’s modulus is 43 GPa, values very similar to those of bone (i.e. bone density of 1.95 g/cm^3^, bone Young’s modulus of 20 GPa). Magnesium fracture toughness is higher than ceramic materials such as calcium phosphates, making it an adequate candidate for load-bearing implant manufacture. Unfortunately, the main drawback of the Mg is that it has a low compressive yield strength of about 82.5 MPa compared to natural human bone, characterized by an average value of 155 MPa. Recently, alloying with biocompatible elements was performed, and Mg-based compounds with adequate mechanical properties were achieved [98, 99]. Regarding the crystalline structure of the Mg alloys, they exhibit hexagonal close-packed structures that are characterized by a low number of slip planes. Consequently, they have a reduced ultimate tensile strength (UTS) of about 50 MPa [100].

It is well-known that the material mechanical properties are drastically diminished during the Mg-based alloy degradation process, and adequate analysis must be performed *in vivo* for both types of implants: the trauma ones used to fix the fracture and the regenerative ones used to solve the problem of bone critical-size defects. Also, *in vitro* experimental determinations can be very helpful in proving the strong dependence between the degradation process of the material and mechanical properties.

Marukawa *et al.* [71] compared uncoated Mg-Y-RE-Zr (WE43) and PLLA screws used as degradable implants in a canine fracture model. Their attention was devoted to mechanical strength and implant stability for the Mg-based alloy implant. WE43 material is an alloy that exhibited improved corrosion resistance and mechanical strength compared to pure Mg. The rare earth (RE) component decreased the corrosion rate through intermetallic compound or solid solution formations by increasing the oxide film thickness combined with an additional magnesium hybrid layer apparition. These phenomena proved to help improve mechanical properties. In the case of anodized or monolithic WE43, the tensile strength was about 280 MPa. The *in vivo* investigation concluded that the anodized WE43 screw behaved very well from the mechanical point of view because only one screw was broken after 4 weeks, and one screw loosened after 12 weeks. The last unwanted result can be explained through the implant degradation and diminution of the protective oxides/hydroxides layer formed during the anodization procedure. Chou *et al.* [96] investigated the degradation process influence on the mechanical stability of uncoated Mg-Y-Zn-Zr-Ca pins introduced in a non-immobilized rat femoral fracture. Based on micro-CT scan investigations, progressive degradation of the intramedullary pins was put in evidence, which was characterized by a reduction of the cross-sectional area. It was noticed that a more pronounced reduction of the mechanical properties appeared in the fracture vicinity, perpendicular to the defect, in the site where the stress was supposed to have the maximum value. It was concluded that even in the case of applied stresses with values lower than yield strength due to the combined action of the mechanical loading and corrosive medium that is present in the animal body, an embrittlement phenomenon occurred. This was linked in some cases with the premature failure of the implant. Local corrosion regions were noticed at the end of the pins and attributed to the imperfect implant manufacturing method. Another aspect that must be considered is the fact that a higher exposure of the implant in the fracture zone at surrounding fluids occurred due to bone absence. Zhang *et al.* [69] developed an innovative uncoated Mg-based intramedullary nail that accelerated in a high amount the bone-fracture healing process in rats. In comparison with the control group, the mechanical strength of the bone was improved in the case of Mg implants, and it was concluded that even though the material is a biodegradable one, it offered sufficient mechanical strength during 12 weeks of treatment because the xylenol/calcein staining ratio was superior and completion of bone remodeling was achieved. Han *et al.* [75] manufactured pure uncoated Mg screws for fracture fixation in a rabbit animal model. Scanning electron microscopy (SEM) images put in evidence the importance of the degradation process on the mechanical integrity of the implant because, at 24 weeks, the screws were barely visible. Also, a weight reduction of 24.6% was detected after 4 weeks and 39.2% at 24 weeks. From the mechanical point of view, the bending force decreased after 4 weeks due to the accelerated degradation process of the Mg screw under *in vivo* load-bearing conditions. Then the bending force was attenuated as a consequence of weight loss. Krause *et al.* [101] investigated the mechanical properties variation of pin osteosynthesis implants made from uncoated Mg-Ca, Mg-Li-Al-RE (LAE442), and Mg-Y-RE-Zr (WE43). The pins were introduced in the medullary cavity of the tibia in 30 rabbits. Before implantation, based on the three-point bending testing method, the maximum initial strength was obtained for the LAE442 alloy (255.7 ± 5.69 N), and the minimum value was achieved in the case of Mg-Ca (178.8 ± 25.1 N). The WE43 alloy was characterized by a value of 122.23 ± 23.65 N. Measurements were carried out 3 months after implantation, and the maximum strength value was in the case of WE43 (185.5 ± 15.64 N), and the lowest value for Mg-Ca (115.42 ± 9.66 N). After 6 months, the LAE442 exhibited a maximum value of 134.68 ± 14.7 N and Mg-Ca alloy a minimum value of 52.9 ± 6 N. It can be concluded that the degradation of WE43 and Mg-Ca alloys linearly decreased over time since the LAE442 material degraded faster in the first 3 months and then this process stabilized for the next 3 months. Kannan *et al.* [102] conducted a study regarding the uncoated Mg-Y-RE-Zr-Ca (AZ91Ca) mechanical properties inserted into an SBF corrosive media showed that the alloy had a lower UTS and elongation rate (ER) in SBF (i.e. 106 ± 10 MPa—UTS, 3.5 ± 0.2%—ER), by comparing these values with those measured in air (126 ± 5—UTS, 4.6 ± 0.3%—ER). This low decrease in the mechanical properties can be attributed to the fact that due to alloy insertion into SBF, a protective calcium phosphate layer on the alloy surface appeared and decreased the degradation process. Compared to the commercially available Mg-Y-RE-Zr (AZ91) alloy, the mechanical properties behaved better during corrosion because the AZ91 did not contain the calcium chemical element. Van Gaalen *et al.* [103] numerically studied the correlation between the pitting corrosion and mechanical properties of a rare-earth-based Mg alloy. They used 3D cylindrical geometries similar to those present in the dog-bone samples (diameter of 3 mm and a gauge length of 18.95 mm), and based on finite element simulations, a combined model with corrosion and uniaxial tests was developed. Before the simulations, the authors performed immersion tests in SBF for 28 days with weekly analysis. The numerical results provided relationships between mechanical properties such as maximum strength, Young’s modulus, and strain that corresponded to the maximum value of the strength and surface-based pit formation. It was concluded that the most important parameter is the minimal cross-sectional area of the implant, which is directly linked to the remaining mechanical strength, but it cannot predict the reduced elastic modulus of the samples. It was noticed that in some cases, the numerical simulations failed to create the experimentally observed pit density due to the limits imposed by programs for the finite element size. The authors concluded that the modification of the mechanical properties must be correlated with *in vitro* studies.

Cheng *et al.* [91] investigated the mechanical properties of open-porous magnesium scaffolds that exhibited pipe-like pores with two pore sizes of 250 and 400 μm. The compressive strengths of the two types of scaffolds were 41.2 ± 2.14 and 46.3 ± 3.65 MPa, and their Young’s moduli were 2.18 ± 0.06 and 2.37 ± 0.09 GPa. The authors explained that open-porous scaffolds usually have much better mechanical properties than other similar structures. Micro-CT scans revealed that both scaffolds’ density and volume decreased 8 weeks after implantation surgery, and at 16 weeks, the Mg scaffolds were almost fully dissolved and replaced by new bone. It can be concluded that the developed structures had adequate mechanical properties at the beginning of the treatment, and as long as the degradation in physiological media occurred, they lost their mechanical stability and helped due to the bioactive character of Mg^2+^ ions at the new bone apparition. Wang *et al.* [80] made Mg-Nd-Zn-Zr (JDBM) scaffolds coated with brushite or MgF_2_. The compressive variations of the scaffolds were similar to those obtained in the case of cancellous bone. The yield strength of the brushite-coated samples was equal to 6.87 ± 0.16 MPa, and Young’s modulus of 0.40 ± 0.09 GPa was measured. These values were close to those of normal cancellous bone (Young’s modulus—0.02–0.5 GPa; yield strength—1–12 MPa) and superior to those obtained in the case of MgF_2_-coated scaffolds. Based on X-ray investigations, rabbits’ radius segmental bone defect was analyzed at 4, 12 and 24 weeks. The implant shadow was completely visible at 4 weeks, but after 24 weeks, the brushite-coated scaffolds were degraded, and new bone formation was seen in the implant neighborhood. Xie *et al.* [97] put in evidence the mechanical properties of biodegradable 3D woven magnesium scaffolds uncoated and coated with PLA with standard architecture (STD) and modified architecture with an increased porosity grade (HPFW). The load–displacement characteristics were determined based on bending tests. It can be noticed that higher values were obtained in the case of PLA-coated samples for the bending strength (i.e. (1.67 ± 0.05)—(19.23 ± 4.07) MPa—STD geometry and (1.34 ± 0.02)—(19.09 ± 2.15) MPa—HPFW geometry) and Young’s modulus (i.e. (29.15 ± 3.13) – (1259.14 ± 153.78) MPa—STD geometry and (23.93 ± 3.67) – (1021.75 ± 202.45) MPa—HPFW geometry). To evaluate the mechanical properties evolution in physiological media, the scaffolds were implanted in surgically created defects in mice. No abnormalities in how wounds heal or significant negative effects were noticed. Also, the PLA-coated Mg scaffolds can enhance vascularization, especially for the scaffolds with bigger pores, according to *in vivo* animal experiments. It was concluded that the scaffolds exhibited adequate mechanical properties and facilitated the critical size defects in mice healing by promoting new bone formation and providing sufficient mechanical support.

**Figure 4.**
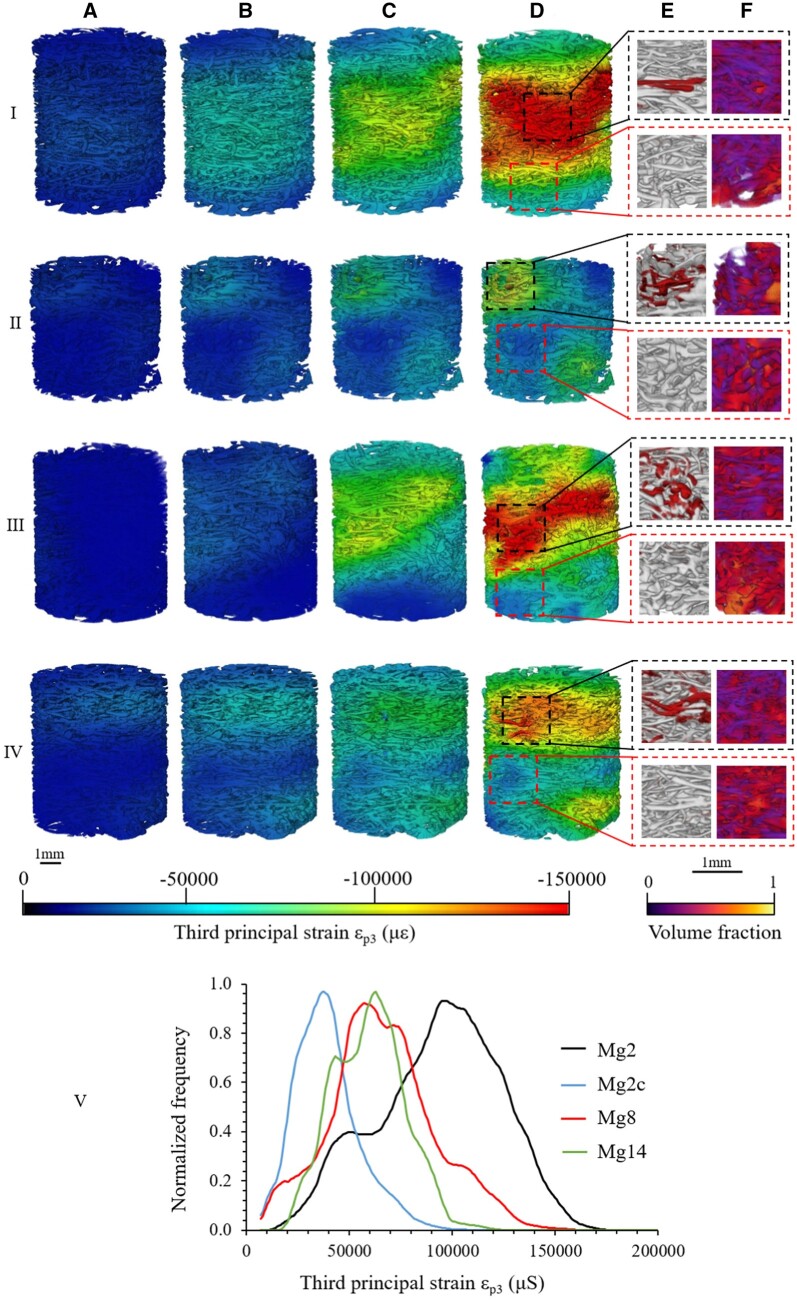
The third principal strain map at (**A**) 1%; (**B**) 3%; (**C**) 6%; (**D**) 10% for Mg alloy sample at (**I**) 2; (**II**) 2 cyclic; (**III**) 8, (**IV**) 14 days of *in vitro* corrosion due to immersion in HBSS; (**V**) Normalized frequency versus third principal strain. (**E**) XCT image reconstruction (red figures represent the material loss); (**F**) volume fraction distribution linked to low-strain (lower dotted square parts) and high-strain (upper dotted square parts) [104]. (Figure is licensed under CC-by 4.0.)

Bonithon *et al.* [104] developed open-porous Mg-Y-Zn-Mn (WZM211) scaffolds coated with MgF_2_ and analyzed their *in vitro* corrosion behavior under cyclic loading. As corrosion media, the HBSS was used, and its effect was combined with *in situ* cyclic compression (30 N/1 Hz, −150 000 loads achieved in 2 days). The X-ray computed tomography mechanical results were correlated with digital volume computation. The samples were subjected to an accelerated corrosion process at 37°C and 5% CO_2_ for 2, 8 and 14 days. It was observed that the mechanical solicitations could be applied only in the first 2 days because on the third day, under the combined effect of the cyclic mechanical load and the Hanks’ solution, a full degradation of samples was achieved. These samples, denoted as Mg2c, had appropriated values of Young’s modulus in comparison with the control ones (Mg2) that were only immersed in HBSS and with no applied mechanical loads (i.e. 86 ± 8 MPa for Mg2c and 89 ± 39 MPa in the case of Mg2). The yield stress of Mg2c samples was found to be comparable with that measured in the case of alloys immersed for 14 days in HBSS (Mg14) (i.e. 2.9 ± 0.5 MPa—for Mg2c and 3.0 ± 1.3. MPa—for Mg14). The local third principal strain was computed, and it was noticed that an increased corrosion time favored a diminished strain accumulation. In the case of samples Mg2c, Mg14, and Mg2, there were found the following values −40 791 ± 1321 με, −60 093 ± 2414 με and −91 000 ± 6361 Mg2 (Figure 4). These findings were correlated to those of CT tomograms, and it was observed that a lower volume fraction and an increased material loss were present in the high-strain zone vicinity. It can be concluded that the cyclic mechanical load exhibited an important influence on the degradation of samples, and the MgF_2_ offered high protection against corrosion in the physiological medium in the absence of mechanical solicitations.

Dong *et al.* [77] investigated the correlation between the immersion effect in r-SBF and the mechanical properties of Mg-based alloys coated with MgF_2_ and MgF_2_-CaP. Uniaxial compression tests were applied on samples made from uncoated, MgF_2_-coated, MgF_2_-CaP-coated samples and MgF_2_-coated, MgF_2_-CaP-coated scaffolds after 1, 3 and 7 days of soaking in r-SBF. It was observed that the coated samples exhibited higher values of Young’s modulus (i.e. 5 MPa—bare Mg, 8 MPa—MgF_2_-coated samples, and 13 MPa—MgF_2—_CaP-coated samples). Regarding the *in vitro* tests, Young’s modulus of MgF_2_-coated scaffolds had an average value of about 350 MPa and showed a stable character since the yield strength was determined to be around 10 MPa until day 3, followed by a decrease at 6.6 ± 0.4 MPa at day 7. The MgF_2_-CaP-coated samples had a maximum value of about 24 MPa on day 3 for the yield stress and a minimum of 13 MPa on day 7. The Young’s modulus of the MgF_2_-CaP-coated scaffolds exhibited a maximum of 1100 MPa, kept almost constant between day 1 and day 3 of immersion, and a minimum of 400 MPa on day 7. It was concluded that during the *in vitro* degradation process, the mechanical properties of coated Mg-based alloys maintained within the limits of those of trabecular bone due to the fact that the corrosion products are first deposited inside the strut of the scaffolds and produced an increase of the mechanical properties. After day 7 of immersion, the corrosion process advanced, and the scaffold dissolved in a low amount.

As an overall finding, due to the strong link between the degradation process speed of Mg-based alloys and their mechanical properties, the same strategies underlined in the previous subsections, such as alloying and controlling the composition and microstructure of the material and surface modifications or surface coatings must be applied and adapted to the characteristics of the physiological media. A general conclusion from the literature review presented in this section is that polymeric coatings or other surface modifications can enhance the vascularization process and longer maintain the implant's mechanical stability by offering the necessary time to heal the affected hard tissue. These types of investigations are of utmost importance in the implant or scaffold design, and choosing an adequate coating may influence in a high amount the healing process for *in vivo* applications. The interface problem must be carefully addressed, and a combination of mechanical measurements made in the presence of physiological media and material biocompatibility investigations must be further developed in order to obtain an ideal implant that facilities new bone and blood vessel formation and degrades together with its coating after a given time, so removal surgery is prohibited.

## Trauma implants and scaffolds from biodegradable magnesium alloys

In this section, we describe, among others, the main trauma implants used in orthopedy by presenting a comprehensive analysis divided into animal experiments and clinical trials. Few studies are reported in the literature regarding implant surface modifications or coatings for the animal experiments section compared to the number of papers investigating uncoated Mg-based alloys’ efficiency. Even so, the importance of surface treatment must not be neglected due to the fact that research reports good results, such as new bone formation and complete union of fractures. Treatments such as plasma electrolytic oxidation (PEO) and silicon or polymeric coatings applied through different methods exhibited important osteoinductive and osteoconductive properties. By taking into consideration the clinical trials existent in the literature, there are only three producers who sell Mg-based trauma implants, as presented in Trauma implants section. From the commercial marketplace, only one product, MAGNEZIX^®^ CSC 4.8 screw, has a special surface consisting of a dense and porous magnesium-based oxide film obtained after applying an electrochemical conversion. Maybe shortly, more trauma implants will benefit from a surface treatment or coating to preserve the material mechanical integrity for a longer time, increase the corrosion resistance and reduce the side effects such as avascular necrosis related to hydrogen pockets. Complementary in this section, we present examples of experimental coated and uncoated scaffolds used in regenerative medicine. Coatings such as magnesium fluoride films, brushite, or polymeric layers proved to be efficient in improving the scaffold degradation behavior, but supplementary studies are necessary to perform a clinical translation. Last but not least, in this section, we enumerate the main advanced technique for obtaining magnesium trauma implants and scaffolds, such as conventional technologies and 3D printing methods, to provide a clear overview of the way of the implant and scaffolds fabrication procedure.

Bone represents a hard tissue containing cells in a matrix of 90% collagen and 10% amorphous organic substances reinforced by a mineral structure. The main bone mineral components are calcium phosphate (Ca_3_(PO_4_)_2_) and calcium carbonate (CaCO_3_) [105]. As described in Clinical challenges related to bone repair and regeneration section, the bone remodeling process strongly depends on properties that give maximum mechanical strength with low mass. Healing of the bone becomes impossible in the case of critical-size defects. Today the so-called “internal fixation” is possible using an aseptic technique in the case of open fracture reduction and direct fixation with metallic implants [106]. We can define trauma and regenerative implants as medical devices that fix or replace the bone and the joints articulating surface.

### Design and examples

#### Trauma implants

Usually, common orthopedic *trauma implants* include plates, screws, and nails. Also, special implants such as compression screws for ligaments (i.e. anterior cruciate ligament (ACL) screw), mandibular plates and screws, spine cages, or rings for joints can be considered.

##### Animal experiments involving trauma implants

In surgery, different types of plates are used. In order to fix a fracture, the compression plates are involved by bringing together the two fracture ends [107]. They act by providing dynamic pressure on the defect and giving mechanical stability to promote the bone repair process. Fisher *et al.* [108] made magnesium plates from Mg-Y-RE-Zr (WE43MEO) alloy that was chill-casted and contained six-hole locking plates. The implant surface was modified by PEO with a phosphate-based solution. They used the plates in order to fix a mandible fracture model in sheep. Biomechanical tests were performed on sheep heads bought from a butcher. In comparison with the titanium plates (233.3 N), it was noticed that the peak forces at failure were higher for the magnesium group (288.6 N). Regarding the mechanical stiffness, minimal differences were reported compared to the Ti control grou*p* values. The authors concluded that the Mg-based plates showed good mechanical behavior, and compared with Ti control material, they had the advantage of biodegradability, and the need for a second surgery is prohibited. Imwinkelried *et al.* [109] implanted different types of plates and screws made from uncoated Mg-Y-RE-Zr (WE43) alloy in two miniature pig models (Göttingen and Yucatan pigs) to analyze the systemic and local influence of the alloy related to metallic ion emission, degradation process, and hydrogen accumulation. They developed a second model underlining the Mg-based plate and screw performance on bone healing. In the case of Göttingen pigs, a complete mandible osteosynthesis was achieved after 6 weeks of treatment, but unfortunately for the Yucatan pigs, three from seven animals proved instabilities of the implant. The study’s main conclusions were that WE43 alloy uniformly degraded, so the plates or screws did not suffer plastic deformations, no high amount of gas accumulation was reported, and the Göttingen pigs exhibited anatomy of the bone defect more closely to that of the human bone. Hence, as a direct consequence, this type of animal model is adequate in the osteosynthesis analysis of the implants compared to the heavier Yucatan pigs. Chaya *et al.* [94] used pure uncoated Mg plates and screws to fix a rabbit ulnar fracture. They found that the Mg-based trauma implants did not affect the normal healing of the bone and supplementary accelerated it due to the important role of Mg in bone growth and cell differentiation. This study emphasized the importance of the Mg-based alloys in the load-bearing zone treatment, by providing sufficient mechanical integrity and an improved regenerative character, compared with the inert materials.

Buttress plates are designed to hold together fractures of the long bones and are usually used around joints such as ankles and knees [110]. They have defined contours and can be moved with the body. Byun *et al.* [111] developed buttress plates made from WE43 alloy to fix an osteotomy performed in a canine model. It was concluded that the WE43 implants showed adequate mechanical strength for the mid-facial zone. No surface treatment or coatings were applied, and negative drawbacks such as increased corrosion rate or hydrogen emissions were reported. It was noticed that if the corrosion resistance is increased, the developed fracture management systems comprised buttress plates and screws will have the potential to treat the mid-facial defects efficiently.

One of the meetest treatment strategies applied in current medical practices involves locking plates and screws, which is considered a safe procedure. Usually, orthopedic screws tighten up a damaged zone by reducing the gap between bones, and they can be combined with locking plates or can be used alone. Compression screws are used for ligament treatment. The ACL represents a common joint injury, and as fixation devices for the tendon graft, the interference screws are used. The Mg-based interference screws exhibit the advantage of bone growth at the tendon–bone interface, but they are characterized by important drawbacks such as insufficient mechanical strength due to the degradation process speed. Wang *et al.* [112] prepared uncoated Mg-Zn-Sr interference screws dedicated to tendon graft fixation to the femoral tunnel in rabbits. The authors used commercially type poly-lactide (PLA) screws as control samples. The degradation behavior of the implants was analyzed *in vivo* with the help of computed tomography at 0, 6, 12 and 16 weeks. It was found that the Mg-based interference screw was fully degraded after 12 weeks, and a reduced peri-tunnel bone loss was observed in comparison with the PLA control group. After 16 weeks, a high level of new bone formation was seen. The results proved that the Mg-Zn-Sr alloy is a suitable candidate for ACL reconstruction. Cheng *et al.* [113] proved that pure uncoated Mg interference screws supported early graft incorporation in a rabbit animal model. They showed that due to the fact that graft degradation is a vital process in ACL reconstruction, the Mg-based implants exhibited superior performance in comparison with Ti screws used as control. The tendon graft that was fixed based on Mg screws had good biomechanical properties with a superior collagen fiber area at the tendon–bone interface. A uniform corrosion behavior for Mg interference screws was put in evidence, and it was concluded that pure Mg screws modulated the MMP-13 expression by reducing it and maintaining Collagen II fibers formation during the collagen deterioration process. A biological attachment of the tendon graft was obtained. Mau *et al.* [114] proposed an innovative design of an ACL interference screw made from Mg-based alloys using the finite element analysis method. Based on *ex vivo* animal experiments, it was observed that a screw stripping phenomenon was in the case of a full torque applied to the implant during surgery to prevent a full insertion and a poor fixation of the graft. The authors analyzed the mechanical stresses in the screw head, then used this information to improve the implant design. Six drive models such as turbine, hexagonal, torx, quadrangle, trigonal, and trilobe were used. It was noticed that the torque value for screw insertion was about 2 Nm, and the maximum shear stress had the highest value in the turns or corners of the drive. A stripping effect was put in evidence in the case of values higher than 193 MPa (Mg yield stress). It was concluded that by modifying the implant design, the surgical success rate could be increased, and the number of failure cases could be reduced.

The intramedullary nail is a metal rod inserted into the medullary cavity of long bones such as the tibia or femur to fix a fracture and permit the patient to recover within a few weeks. Jähn *et al.* [115] showed that intramedullary nails manufactured from uncoated Mg-Ag enhanced callus formation during fracture healing in a mouse animal model. The *in vivo* implant degradation process was extensively analyzed, and the nails were implanted in mice without or with a femoral shaft fracture. The animal tissues were examined. Compared with the *in vitro* studies, nail degradation was accelerated *in vivo.* As control implants, the authors used steel nails. The main conclusion was that increased bone formation and decreased bone resorption accompanied by more considerable callus formation during the first 21 days of treatment were observed for the mice group treated with Mg-based nails. It can be noticed that the Mg-Ag alloy had a benefic effect on fracture healing, and it prevented the apparition of infections due to the Ag antibacterial effect. Krämer *et al.* [116] investigated biocompatibility, corrosion resistance, and biomechanical properties of uncoated Mg-Li-Al-Se-Mn (LAE442) intramedullary nail. In order to evaluate the mechanical behavior, a cyclic fatigue test was applied in distilled water and HBSS at the human body temperature (37°C) until nail failure or 500 000 cycles. The results showed that in HBSS, a nail broke after 70 000 bending cycles, and in water, four of the five implants remained geometrically intact at the end of the mechanical tests. Regarding the material’s corrosion, an increased weight loss and hydrogen emission was noticed in HBSS. The cytotoxicity of the alloy was tested on the fibrosarcoma cell line (L929), and it was observed that good cell viability was detected for diluted extracts (12.5%, 25%, and 50%), and in the case of 100% extract the cellular viability was lower than 70%. It was concluded that LAE442 is adequate for load-bearing applications, but an anti-corrosive coating must be applied to prevent mechanical integrity loss. Rossig *et al.* [117] implanted uncoated Mg-Li-Al-Se-Mn (LAE442) intramedullary nail in a sheep model. A biomechanical examination of the implant followed by a finite element simulation put in evidence adequate mechanical properties such as average stiffness of 2179.34 ± 24.69 N/mm and an average maximum force of 3443.61 ± 57.08 N. In the case of retrieved nails, reduced values were obtained (average stiffness of 1769.03 N/mm and average maximum force of about 2621.68 N). Computed tomography proved a slight degradation of the implant at 24 weeks and a good osteointegration of the nail. Adverse effects (ADE), such as high hydrogen accumulation, were not reported. It can be concluded that LAE442 is a good candidate for intramedullary nail manufacture, and it can be used in clinical trials in humans.

In the case of spine trauma, one of the most used types of implants is the spine cage, which is surgically placed and creates a space between the affected vertebrae [118]. This implant allows bone growth inside and can become an integrated part of the human body. Guo *et al.* [119] developed a high-purity uncoated Mg interbody cage. They studied degradation, biocompatibility, and interbody fusion based on a goat cervical spine model. Regarding the mechanical properties, a universal testing machine was used, and the implant Young’s modulus in compression was about 609.43 ± 52.16 MPa, and the compression strength was equal to 151.59 ± 8.34 MPa. The *in vivo* investigation consisting of spinal cage implantation between C2-3 and C4-5 vertebrae in a goat animal model was assessed based on radiological examination. It was concluded that the fusion area is lower than 30%, and the results are considered worst compared to the autogenous ilium group. The degradation tests pointed out a faster degradation process in the first 3 weeks after surgery, followed by a stable behavior. There were no reported adverse reactions, and the disc space between segments did not modify at 24 weeks after surgery, showing a good mechanical strength of the implant. Zhang *et al.* [120] manufactured a silicon (Si)-containing coated Mg-Al-Zn (AZ31) cage and implanted it in C3/C4 and C5/C6 zones in goats. The control group had autologous iliac crest bone implants. At 3 weeks after implantation, the intervertebral Mg levels increased and persisted at 12 weeks. This observation is directly linked to the rapid corrosion of the implant. The level of Mg decreased at 24 weeks. Compared with the control group, low interbody fusion was observed, and it was concluded that excessive intervertebral Mg accumulation is the main cause of implant failure. The authors suggested that more research must be done in these fields, and innovative coatings solutions must be found to control the metallic ion emission and degradation process.

Mg-based ring devices for joint treatment are an innovative approach that consists of a ring-shaped device with adequate suture, which offers high joint stability concomitant with the load of the ligament and the insertion places. Usually, these implants are applied in ACL injury treatments. Farraro *et al.* [121] designed a monocrystalline Mg ring combined with a new suture implantation technique to treat a transected ACL problem and stabilize the knee. They observed that the Mg ring was suitable for restoring the normal function of stifle joint stability and helped the regeneration of ACL. The implant decreased the mechanical load that acted on the meniscus, and no secondary injuries were reported. The goat model was a suitable choice for Mg-based ring effect investigation, but significant differences can occur when human subjects are involved due to anatomical differences and different ranges of motion. The main conclusion was that this study showed the potential of Mg rings in ACL injury treatment, but more research must be done in this direction. Wang *et al.* [122] made a finite element analysis for an Mg ring used in the mechanical augmentation process of an ACL rupture. A 50% reduction in the anterior-posterior tibial translation was observed after the ring repair effect. Supplementary, the *in situ* forces simulated in the ACL zone were restored at 60% from the performances of a healthy ACL. At the femoral insertion, a value for the maximum Von Mises stress up to 71% from the normal value was computed. It can be concluded that the Mg ring could not completely restore the ACL functions, and the existing designs must change to generate a mechanical augmentation of the implant.

The most challenging trauma implants are the Mg-based hybrid systems that offer stable mechanical support for fracture healing, providing, at the same time, an accelerated healing process at load-bearing sites. Tian *et al.* [123] developed an innovative Mg/Ti hybrid system for long bone fracture fixation. Based on finite element analysis, they investigated if the proposed device offered enough mechanical support for fractures localized in load-bearing skeletal sites. The efficacity of the hybrid system was analyzed *in vivo* by performing a “Z-shaped” osteotomy at the mid-shaft of the rabbit tibia. Two groups of animals were set. In the first one (Mg/Ti group), the fixator was made from Ti, and the screws from PLA film coated pure Mg to avoid the electrochemical corrosion induced by the direct contact between the two different metals. Regarding the second group (Ti/Ti control group), the system was made entirely of Ti. After 12 weeks, different investigations such as a four-point bending mechanical experiment, radiographic images, histomorphometric and histological analysis were performed. The medical images put in evidence for the Mg/Ti group a larger callus formation, and the biomechanical test made on rabbit bone evidenced a higher mechanical strength. A faster endochondral ossification with higher expression of OCN and COL1 was obtained in comparison with the Ti/Ti group. It was concluded that the developed hybrid system was very efficient because Mg screws provided sufficient mechanical support and upregulated the local CGRP secretion, which ultimately affected the acceleration of callus remodeling and mineralization. Hybrid fixation systems promote osteoporotic fracture healing and could reduce the refracture rate. In Ref. [124], it is underlined that Mg-based hybrid systems can be successfully applied in the case of osteoporotic bone fractures because these patients exhibit a delay in callus formation and bone remodeling. Usually, stainless steel or titanium components offer enough mechanical support for fractures at load-bearing sites since the Mg parts enhance the healing process during its degradation.

This section shows that different animal models are used for *in vivo* tests of Mg-based alloys. The model must be chosen following the implant application. However, a standard procedure to establish the implant location and size is not currently defined. From an anatomical point of view, there are significant differences between blood flow and water content in miniature pigs, sheep, goats, rabbits, dogs and rodents. The researchers can use one or more animal models to analyze the *in vivo* behavior of different trauma implants and different coating technology effects on living bodies.

##### Clinical trials involving trauma implants

Clinical experiments described in the literature showed the high biocompatibility of the Mg-based trauma implants with minimum foreign body responses [125]. Nowadays, only three Mg alloys for orthopedic implant manufacture receive approval for human use. One design made of high-purity (99.99%) Mg (Eontec, Dongguan, Guangdong) is used mainly in China. The second one is manufactured from Mg-Y-RE-Zr (WE43), developed by Syntellix AG (Hanover, Germany), and sold under the commercial name of MAGNEZIX^®^. The third implant design made of Mg-Ca is produced by U&I Corporation (Gyeonggi, Korea) and found on the market under the RESOMET^TM^ name [47, 126, 127].

One of the most important designs in trauma management is the Mg-based Herbert screw that can be defined as a headless, variable pitch, cannulated compression screw. It is usually used to treat scaphoid [128–130] humerus [131], malleolar fractures [132–134] hallux valgus [67, 135] and for compressive osteosynthesis stabilization [136]. These types of screws exhibited special characteristics, such as being fully introduced into the bone without tissue inflammation or protrusion. Percutaneous application is possible because these screws are cannulated and can secure the fracture better. Other trauma implants made from Mg-based alloys used in clinical trials are classical compression screws, interference screws, and pins [137].

Könneker *et al.* [128] compared Mg-based headless Herbert screws and Ti Herbert screws for scaphoid fracture treatment. A number of 190 patients were chosen, and the follow-up time was set at 1 year per patient. Three endpoints were established to check the treatment results. The first one was related to the patient-rated wrist evaluation score after 6 months, while the second one investigated the bone union, the existence of ADE and wound healing possibility until 6 months. The third endpoint was directly linked to the implant presence in the magnetic resonance images (MRI). The study was performed to analyze if the Mg-based screw had superior performance over the Ti one. The non-inferiority regarding the first two endpoints of the Mg was put in evidence in comparison with the Ti in the case of scaphoid fractures, and regarding the MRI images, the absence of the Mg-based screws was noticed and compared to Ti screws, a better osteoconduction was observed.

Sonnow *et al.* [138] compared the intensity of artifacts between a biodegradable Mg Herbert screw with a similar implant made from Ti. The screws were characterized by the same geometrical dimensions of 3.2 mm × 20 mm, and images of them were taken based on different methods such as multidetector computed tomography, digital radiography, high-resolution flat panel, and MRI. The testing media were *in vitro* or after the implantation of the screws in a fresh chicken tibia. By analyzing all the obtained images, it was concluded that the Mg-based Herbert screws generated a low number of artifacts, and post-surgical follow-up became more facile and accurate.

**Figure 5.**
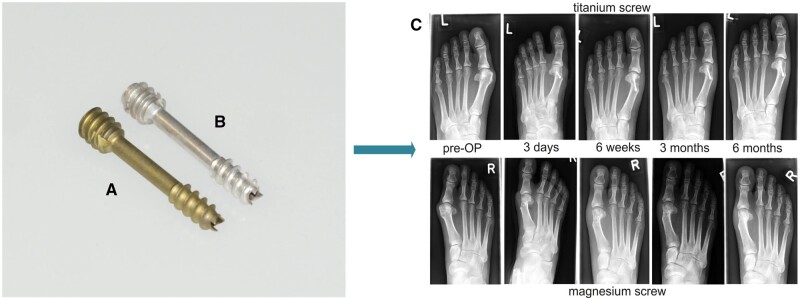
A comparison between Ti- and Mg-based compression screws. (**A**) Titanium screw from Königsee implantate GmbH; (**B**) MAGNEZIX^®^ compression screw from Syntellix AG; (**C**) preoperative and postoperative medical images of the hallux valgus deformity [67]. (Figure is licensed under CC-by 2.0.)

Windhagen *et al.* [67] conducted a randomized, controlled clinical pilot study that analyzed the differences between Mg-based and Ti-based Herbert screws in the case of 26 patients with hallux valgus. The study duration was set at 2 years, and the follow-up examination was performed on 12 patients from each group. Almost all the patients exhibited good mobility scores and very few side effects were reported. Only one patient in the Mg-based group reported a problem with superficial wound healing, but none of the patients had gas cavities due to Mg-based screw degradation. Regarding the chemical analysis, there were not found increased levels of Mg in the patient blood. The medical images showed no bone erosion, arthritis, or avascular necrosis. In the case of one patient from the Ti group, it was necessary to perform a second surgical intervention to remove the implant after 8 months. The main adverse reactions were postoperative sickness, pneumonia at 5 months after the surgery, and 3 wounds that healed without supplementary interventions. As an overall conclusion, it was demonstrated that the Mg-based Herbert screws are equivalent to the Ti screws from a clinical and radiological point of view (Figure 5).

Aktan *et al.* [131] used Mg-based (Mg-Y-RE-Zr) compression screws and K wires for a comminuted distal humerus fracture to fix the osteochondral fragments. These types of injuries are usually treated with implants placed beneath the articular surface. Unfortunately, complications such as osteolysis, cartilage damage due to metal implant presence, and migration of the K wires could appear, so biodegradable Mg screws and pins are the implant of choice. The authors treated a humerus fracture in the case of a 50-year-old patient. They used magnesium screws to fix the articular surface, a titanium plate to secure the lateral column that was not in contact with the Mg implants to prevent the apparition of the corrosion process and K wires. The patient follow-up was ∼4 months, and a fracture union was reported. Also, the elbow range of motion was between 5° and 130°. The study's main conclusion was that by using bioabsorbable Mg implants, important problems such as secondary surgery for implant removal and osteolysis apparition were prohibited. Supplementary, the Mg ions helped the bone healing process in high amounts.

Kose *et al.* [132] conducted a short-term clinical study in the case of 11 patients with medial malleolar fractures. The follow-up duration was set between 12 and 24 months as a function of the fracture type (Herscovici type C and type B). The authors used to fix simple, trimalleolar, or bimalleolar ankle injuries with magnesium bioabsorbable compression screws. The authors observed excellent treatment outcomes after radiological investigations, and since no patients suffered from complications or needed revision surgery, they concluded that Mg compression screws are an adequate implant for medial malleolar fractures. Acar *et al.* [133] made an interesting comparison between Mg headless compression screw and Ti Herbert screw in the case of biplane chevron medial malleolar osteotomy performed to cure talus osteochondral injuries. They selected 22 patients, from which 11 were treated with Mg screws. After a follow-up of 1 year, a full union of the osteotomy was observed for both groups. Unfortunately, one patient with Ti screw required implant removal due to side effects such as irritation and pain. It was concluded that the Mg-based screws exhibited comparable therapeutic effects to Ti implants, and they can be considered an alternative to the classical treatment in ankle surgery.

Leonhardt *et al.* [139] investigated the mandible fracture treatment in the case of six patients. The injuries were treated with Mg-Y-RE-Zr bioabsorbable headless compression screws. Only one screw penetration through the condylar surface was observed, but surgery for implant removal was not performed. In other cases, an improvement in mouth opening distance and temporomandibular joint performance were reported. Jungesblut *et al.* [140] made a fixation procedure for unstable osteochondritis dissecans (OCD) lesions and displaced the osteochondral fragments. They used Mg-Y-RE-Zr pins (MAGNEZIX^®^) and treated 19 patients, all under the age of 18 years. The follow-up time was set at 11 months. In almost all cases, the Mg-based pins provide high stability in unstable OCD after patella dislocation or trauma. Only in the case of one patient, an implant failure was reported, and revision surgery was necessary. In the other cases, no intraoperative complications were observed. It can be concluded that Mg-based pins are adequate to treat trauma implants for children and young patients with good bone density.

Lee *et al.* [141] showed that Mg-Ca-Zn screws with a diameter of 2.3 mm and a length of 14 mm were used to fix radius fractures in the case of 53 patients. Excellent outcomes were obtained regarding cortical bone formation, and after 12 months, complete healing of the bone was achieved. It was concluded that an innovative Mg-Ca-Zn alloy that does not contain RE is adequate for fracture healing and can be a viable alternative to the classical Mg-Y-RE-Zn.

Another important study was conducted by Yu *et al.* [142] regarding the treatment of 19 patients with femoral neck fractures, which were fixed based on the iliac graft implantation technique combined with pure Mg screw fixation. There were reported no side effects such as avascular necrosis of the femoral head, and it can be noticed that graft implantation and the use of pure Mg implants is a successful way to treat such important injuries. Another interesting case was reported in [143] by Chen *et al.* for femoral head necrosis. The chosen treatment consists of a pedicled bone flap and pure Mg screws. For internal fixation, a nail was used, and after the implant removal, a healthy bone with no necrosis was found. After 2 years of follow-up, an important reduction in pure Mg screw volume was observed, and the radiological investigations showed that the patient necrosis did not progress.

**Figure 6.**
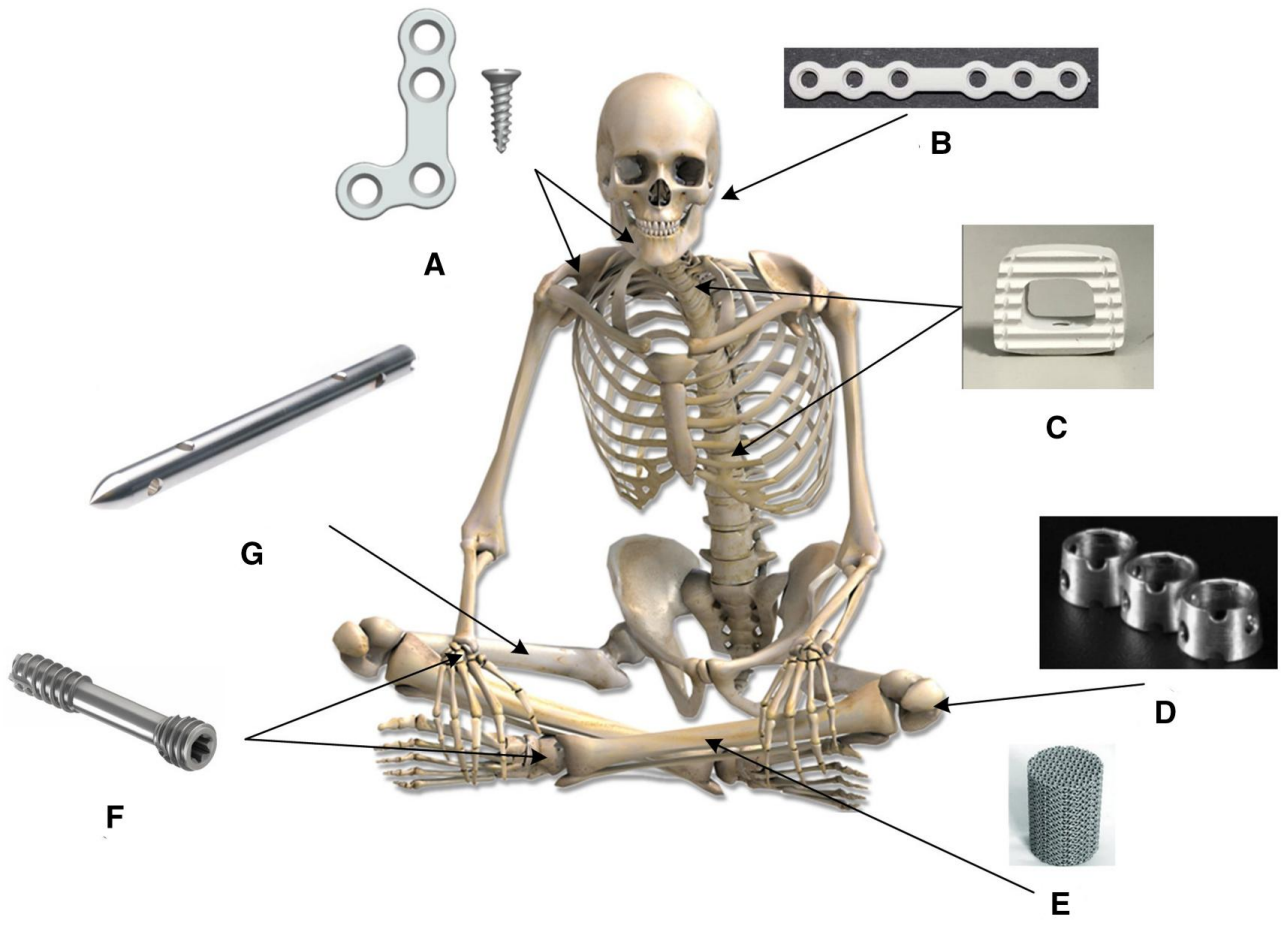
Mg-based implants. (**A**) Buttress plate and compression screw [110] (figure is licensed under CC-by 4.0); (**B**) compression plate [108] (figure is licensed under CC-by 4.0); (**C**) spinal cage [120] (reprinted from [120] Copyright (2023), with permission from Springer Nature); (**D**) rings [121] (reprinted from [121] Copyright (2023), with permission from Wiley); (**E**) scaffold [144]; (**F**) Herbert screw [137]; (**G**) intramedullary nail [116] (reprinted from [116] Copyright (2023), with permission from Elsevier).

Figure 6 presents examples of Mg-based implants used in preclinical and clinical studies.

Numerous studies have demonstrated the effectiveness of Mg-based implants in treating various orthopedic conditions. In comparison to permanent titanium implants, Mg-based screws and pins have shown similar performance without requiring additional revision surgery. Almost all cases presented in this section resulted in complete healing of the fracture. An important observation regarding the MAGNEZIX^®^ CS^C^ 4.8 screw must be mentioned. This screw is dedicated to the treatment of extra- and intraarticular fractures, arthrodeses, pseudarthroses, or osteotomies of medium and small-sized bone, similar indications for talus, metatarsus, calcaneus, and distal tibia, and re-fixation of bone fragments in the proximal tibia or distal femur. It has a unique surface design. Its top layer is electrochemically converted into a porous, dense and adherent magnesium-based oxide film, which is completely bioabsorbable and biocompatible, exhibits a protection function against corrosion, and the degradation phenomenon occurs at a low speed. This trend should be applied for much more trauma implants due to the fact it would be helpful to the reduction of hydrogen emission, concomitantly with good mechanical support for a longer time [137].

Table 2 summarizes different examples of coated and uncoated Mg-based implants used in trauma management.

**Table 2.** Mg-based implants constructive details and materials used in fracture healing

Implant type	Mg-based alloy	Manufacturing technology	Implant characteristics	Animal model/humans	Fracture place	Ref.
*Animal experiments*						
Compression plate and screws	Surface-treated Mg-Y-RE-Zr (WE43MEO)	Chill casting and milling to obtain six hole locking plates, PEO surface treatment	Plates with a thickness of 1.75 and 1.5 mm with sixhole and MaxDrive locking screws with 2.3 × 5 mm	Sheep heads from 1 year old	Mandible	[108]
	Uncoated (Mg-Y-Nd-Zr) WE43	Milling, drilling, threading; the machining was carried out dry	1. Rectangular plate (60 × 6 × 1.5 mm) 2. Six holes plate (60 × 6 × 1.5 mm) with cortex non-locking screws with a diameter of 2 mm 3. Six holes plate (47 × 7 × 1.8 mm) with cortex non-locking screws with a diameter of 2 mm 4. Four holes plate (31 × 7 × 1.8 mm) with cortex non-locking screws with a diameter of 2.7 mm	Götting and Yucatan pigs	1. Nasal bone 2. Frontal bone 3. Rib arches 4. Mandible	[109]
	Pure Mg	Machining in order to adapt the implant to rabbit ulnar geometry	Plates (20 × 4.5 × (1–1.5) mm) and screws with a length of 7 mm	New Zealand white rabbit	Ulna	[94]
	Uncoated (Mg-Y-Nd-Zr) WE43	PM route followed by hot extrusion	Four holes plate with a length of 22 mm and a thickness of 1 mm and cortex screws with a diameter of 2 mm and a length of 5 mm	Miniature pig	Skull	[107]
Buttress plate and screws	Uncoated (Mg-Y-Nd-Zr) WE43	Mg alloy was pre-treated with an extrusion process, and then it was homogenized at 525°C for 8 h and pressed with the help of a three-roll planetary mill; the final step was quenching and aging at 210°C for 16 h	L-shaped plates with four holes with 4.5 mm inter-hole distance, 4.5 mm outer hole size and 1 mm thickness with screws of 6 mm length and 2 mm diameter	Dog	Mid-facial zone	[111]
Interference screws	Uncoated Mg-Zn-Sr	Permanent mold casting at a pouring temperature of 720°C and air cooled; solution treated at 340°C for 4 h and quenched in water followed by extrusion at 320°C and finally, sample cutting with special machines	5 mm rods were machined to achieve the commercially available geometrical dimensions of PLA interference screws	Rabbit	Femoral tunnel	[112]
	Pure Mg	Commercial implant from Suzhou Origin Medical Technology adapted through conventional technologies to rabbit anatomy	Screws with a length of 12 mm, major diameter of 2.7 mm, and core diameter of 2.1 mm	New Zealand white rabbit	Femoral condyle	[113]
Intramedullary nail	Uncoated Mg-Ag	Gravity die casting followed by hot extrusion	Nails with a diameter of 0.8 mm and a suitable length that permits the implant to be inserted underneath the surface of the articular cartilage	C57BI/6J wild-type mice	Right femoral shaft fracture	[115]
	Uncoated Mg-Li-Al-Se-Mn (LAE442)	Casting procedure followed by an extrusion process	Nails with a diameter of 9 mm, four holes, length of 130 mm, and interlocking screws with a core diameter of 2.9 mm, shank diameter of 3.8 mm, thread diameter of 3.5 mm, thread pitch of 1.25 mm and length between 15 and 40 mm	Black-headed mutton pilot sheep	Right medullary cavity of the tibia	[117]
Spine cage	Pure Mg	Conventional technologies	Spine cage with 12 mm × 10 mm × 4–5 mm and a 7° wedge angle	Goat	C2-3 and C4-5	[119]
	Mg-Al-Zn (AZ31) coated with Si	Conventional technologies	Spine cage with a rectangular design similar to the commercially available one developed by DePuy (Cervios; Synthes, DePuy Spine, Raynham, MA, USA)	Goat	C3/C4 and C5/C6	[120]
	Uncoated Mg-Zn	Conventional technologies followed by a micro-arc oxidation (MAO)/Si-containing coating treatment	Spine cage with 12 mm × 10 mm × 4.5–6 mm and a 7° wedge angle, with a porous structure designed in the central 30 mm area with a pore size of 400 μm and a porosity of 45%	Goat	C2-3 and C4-5	[118]
Rings for joints	Mg	Conventional technologies	Rings with a trapezoidal cross-section with bases of 7 and 8 mm and height of 5 mm, one set of notches anterior placed, and 4 staggered holes with a diameter of 2 mm	Boer goats	Transected ACL	[121]
Mg-based hybrid systems	PLA film-coated ultrapure Mg screws and Ti plates	Conventional technologies	AO 2 mm dynamic compression plate with 2 mm Mg cortical screws	Rabbit	Z-shaped fracture placed on the mid-shaft of the right tibia	[123]
*Clinical trials*						
Compression screws (CS)	Uncoated (Mg-Y-RE-Zr) WE43	Commercial implant from MAGNEZIX^®^ Syntellix AG, Hannover, Germany	MAGNEZIX^®^ compression screws (CS) with a diameter of 2.0 mm, a length between 8 and 24 mm in 2 mm stages; MAGNEZIX^®^ CS with a diameter of 2.7 mm, a length between 10 and 34 mm in 2 mm stages; cannulated MAGNEZIX^®^ CS with a diameter of 3.2 mm, a length between 10 and 40 mm in 2 mm stages	190 patients	Scaphoid	[128]
			MAGNEZIX^®^ compression screws (CS) with a diameter of 3.2 mm	Six patients	Three inter-carpal fusions and three scaphoid fixations	[129]
			MAGNEZIX^®^ compression screws	Five patients	Scaphoid fracture	[130]
			MAGNEZIX^®^ cannulated Herbert screws with a shaft of 2.0 mm and a cannulation of 1.3 mm that included two threads with a diameter of 3.0 and 4.0 mm with different pitches	Twenty-six patients	Hallux Valgus	[67]
			MAGNEZIX^®^ cannulated screws with a diameter of 3.2 mm	Twenty-four patients	Hallux Valgus	[135]
			MAGNEZIX^®^ screws with 2.7 mm diameter	Fifty-year-old male patient	Comminuted distal humerus fracture	[131]
			MAGNEZIX^®^ screws	Eleven patients, males and females, with ages between 20 and 78 years old	Medial malleolar fractures (isolated, posterior malleolus, bimalleolar)	[132]
			MAGNEZIX^®^ cannulated screws with a diameter of 3.2 mm and variable pitch	Eleven patients treated with Mg-based implants vs. the control group comprised 11 patients treated with Acutrak^®^, Acumed, Hillsboro, USA Ti-Al-V alloy screws with a diameter of 3.5 mm and variable pitch along the implant length	Biplane chevron medial malleolar osteotomy in the osteochondral lesions of the talus	[133]
			MAGNEZIX^®^ CS, Syntellix AG, Hanover, Germany screw with a length of 24 mm and a diameter of 3.2 mm	Forty-three-year-old female	Bimalleolar ankle fracture type AO 44-B2.3	[134]
Pin	Uncoated (Mg-Y-RE-Zr) WE43	Commercial implant from MAGNEZIX^®^ Syntellix AG, Hannover, Germany	MAGNEZIX^®^ CS, Syntellix AG, Hanover, Germany pins with a diameter of 1.5 mm or 2 mm, and a variable length between 14 and 28 mm	Nineteen young patients with age under 18 years	Nine patients with unstable OCD lesions, eight patients with a displaced osteochondral fragment following dislocation of the patella, and two patients with posttraumatic osteochondral fragment	[140]
Screw	Uncoated Mg-Ca-Zn	–	Mg-based screws with a diameter of 2.3 mm and a length of 14 mm	Fifty-three patients	Distal radius fracture and scaphoid nonunion	[141]
	Pure Mg	Conventional methods	Special design of pure Mg cannulated screws for the vascularized iliac graft fixation	Nineteen patients	Femoral neck fracture	[142]

#### Regenerative bone scaffolds

Bone scaffolds are considered dedicated platforms used to repair a defect or help in bone regeneration when an important quantity of tissue is missing [8]. The main design features that must be followed when a scaffold is manufactured are mechanical properties, biocompatibility, osteoinductivity, biodegradability, porosity, antimicrobial characteristics, osteointegration and osteogenesis. Two-dimensional scaffolds help repair small bone defects due to the fact that a better interaction between biomaterials and cells becomes possible. At the same time, the 3D devices must have an adequate porosity, characterized by a pore size of a maximum 500 μm, to facilitate bone growth without a high impact on mechanical properties and stability. The scaffold is a biomechanical medium that is beneficial to cell differentiation, proliferation, adhesion and dissemination of nutritional substances and oxygen. Sometimes such devices permit the cells, growth factors or drug encapsulation to restore bone functionality and treat important health issues such as infections or cancers. Besides the scaffold porosity and mechanical properties, another important factor that must be considered is the implant surface. The topological aspects, such as surface roughness and chemical composition, manifest an important influence on cell viability, proliferation and adhesion to the scaffold. The elasticity modulus of the scaffold material must exhibit values close to those of the human bone and offer sufficient mechanical support during new bone formation and until complete bone development is finished. An ideal scaffold must be biodegradable, and its by-products highly biocompatible [8, 144].

Taking into consideration the outstanding mechanical and biological properties of Mg-based alloys, many studies [78–80, 91, 97, 145–150] proposed it as an excellent candidate for scaffold manufacture in bone tissue engineering domain. Table 3 presents examples of the design and application of coated and uncoated Mg-based scaffolds used in regenerative medicine for bone tissue engineering.

**Table 3.** Examples of scaffold design and applications in bone tissue engineering

Mg-based alloy	Manufacturing technology	Implant characteristics	Cell lines/Animal model	Defect place	Ref.
Uncoated Mg-Zn	Extrusion-based AM/3D BioScaffolder printer (BS 3.2, GeSim, Germany	Cylindrical porous scaffolds with a height of 12.64 mm and a diameter of 12.38 mm, exhibiting a lay-down pattern of 0°/90°/0° and a design made with software associated with the 3D printer; the as-printed Mg-Zn was characterized by a strut width of 581.2 ± 14.9 μm, a strut spacing of 343.9 ± 18.5 μm and a porosity of 58.3 ± 1.6 %; the as-sintered Mg-Zn had the strut width of 516.1 ± 15.3 μm, the strut spacing of 331.7 ± 39.5 μm and a porosity of 50.3 ± 3.4 %	MC3T3-E1 cells (mouse pre-osteoblasts)	–	[145]
Uncoated Mg-Nd-Zn-Zr (JDBM)	SLM/3D-printing machine (SLM150, ZRapid Tech Co., LTD, China)	Scaffolds with diamond-shaped unit cells were made based on Materialise Magics (version 15.0, Materialise, Leuven, Belgium); the pore size was between 300 and 400 μm, and the porosity was equal to 80.0%	RAW 264.7 (monocyte/macrophage-like cells collected from Abelson leukemia virus modified murine cell line	–	[78]
Uncoated Mg-Y-RE-Zr (WE43)	SLM (system components: single-mode ytterbium fiber laser IPG YLR-200; galvanometric scanner SCANLAB hurrySCAN 20, and f-theta focusing lens SILL S4LFT 3254/126)	Cylindrical porous specimen with a height of 11.2 mm, a diameter of 10 mm, a pore size of 600 μm, a strut size of 400 μm and a relative density of 67% designed as a diamond lattice	MG63 human osteosarcoma osteoblasts	–	[146]
Uncoated Mg-Y-RE-Zr (WE43)	Laser powder bed fusion (LPBF) (Aconity mini 3D printer)	Cubic open porous scaffolds (10 × 10 × 10 mm^3^) with a body center cubic structure and three nominal strut diameters (250, 500, 750 μm) and two pore sizes (720, 860 μm)	L-929 mouse fibroblast	–	[147]
Uncoated Mg-Zn-Zr	Binder jetting (inhouse-modified 3D printer)	Scaffold with distributed pores and dispersed elements such as Mg, Zn, Zr; a relative porosity of about 13% and an open porosity index of about 95%	–	–	[148]
Mg-Li-Al-Se-Mn (LAE442) coated with MgF_2_	Gravity die casting with casting molds for the scaffolds made with a 3D printer (Solidscape, Inc., Merrimack, USA)	Two types of scaffolds with a diameter of 4 mm and a height of 5 mm were made. The first model had an average pore size of 400 μm, a porosity of 43.4%, and a strut thickness of 0.3–0.4 mm. The second type of implant exhibited an average pore size of 500 μm, a porosity of 41.4% and a strut thickness of 0.4–0.5 mm. All the developed implants were coated with MgF_2_	Female Zika rabbits	6 mm deep hole placed into the greater trochanter	[79]
Mg-Nd-Zn-Zr (JDBM) coated with brushite	Template-replicating method	Scaffolds with main spherical pores (400–450 μm) and smaller pores (150–250 μm) interconnected between the main pores.	Rat bone marrow mesenchymal stem cells obtained from femurs of SD rats/SD rats	Femoral condyle defect with a diameter of 3 mm was surgically created on both sides of the animal’s legs	[80]
Pure Mg coated with MgF_2_ layer formed during the fabrication process	TWSH	Two scaffold models were produced. The first one had a pore size of about 250 μm, a total porosity of 54.78 ± 2.67%, a strut size of 0.28 ± 0.04 and a surface area of 235.98 ± 7.47 mm^2^. The second model exhibited a pore size of an average value of 400 μm, a total porosity of 54.31 ± 3.10, a strut size of 0.36 ± 0.02, and a surface area of 204.5 ± 8.01 mm^2^	MG63 human osteosarcoma osteoblasts/New Zealand white rabbit	A hole with a 3 mm diameter and 8 mm deep was made in the lateral epicondyle of the animal	[91]
Mg-Zn-Ca-Mn (ZMX100) coated with PLA	3D weaving with Mg wires	Scaffolds that contain three layers of warp wires and four layers of fill wires (diameter of 300 μm) interlaced with the help of thickness *Z* wires (diameter of 250 μm) were developed. Two architectures were analyzed, one with lower porosity and the other with higher porosity	ASCs adipose cell tissue/Athymic nude (immunocompromised) mouse	Bilateral intramuscular defects placed in the quadriceps muscles	[97]
Uncoated Mg-Al-Zn (AZ91)	Foam casting process	Scaffolds with two mesh sizes defined by two PPI (pores per inch) values (10 PPI and 20 PPI) with a 3-mm pore size	–	–	[149]
Pure Mg	PM using sucrose spacer agent	Scaffolds with 60% porosity and pore size between 400 and 600 μm exhibiting two designs (one with a filled center and the other one with a hollow center) coated with MgF_2_	–	–	[150]

To prevent the failure of a scaffold, it is important to consider solutions that can enhance its long-term stability. One such solution is to modify the scaffold’s surface through surface coatings or alloys with other materials. Two commonly used techniques are deposited coatings, which are applied through dip coating, spraying, spin coating, or immersion and conversion coatings, which involve the scaffold undergoing an electrochemical or chemical reaction to form a ceramic-like coating [151]. However, it is important to note that increasing the scaffold’s surface area through porosity can actually accelerate its corrosion and lead to failure.

### Advanced technique for obtaining magnesium trauma implants and scaffolds

The techniques used for Mg-based implant and scaffold manufacture are very important because they directly impact the implant features such as biocompatibility, mechanical properties and degradation rate. They can be divided into conventional and 3D printing technologies.

#### Conventional technologies

**Figure 7.**
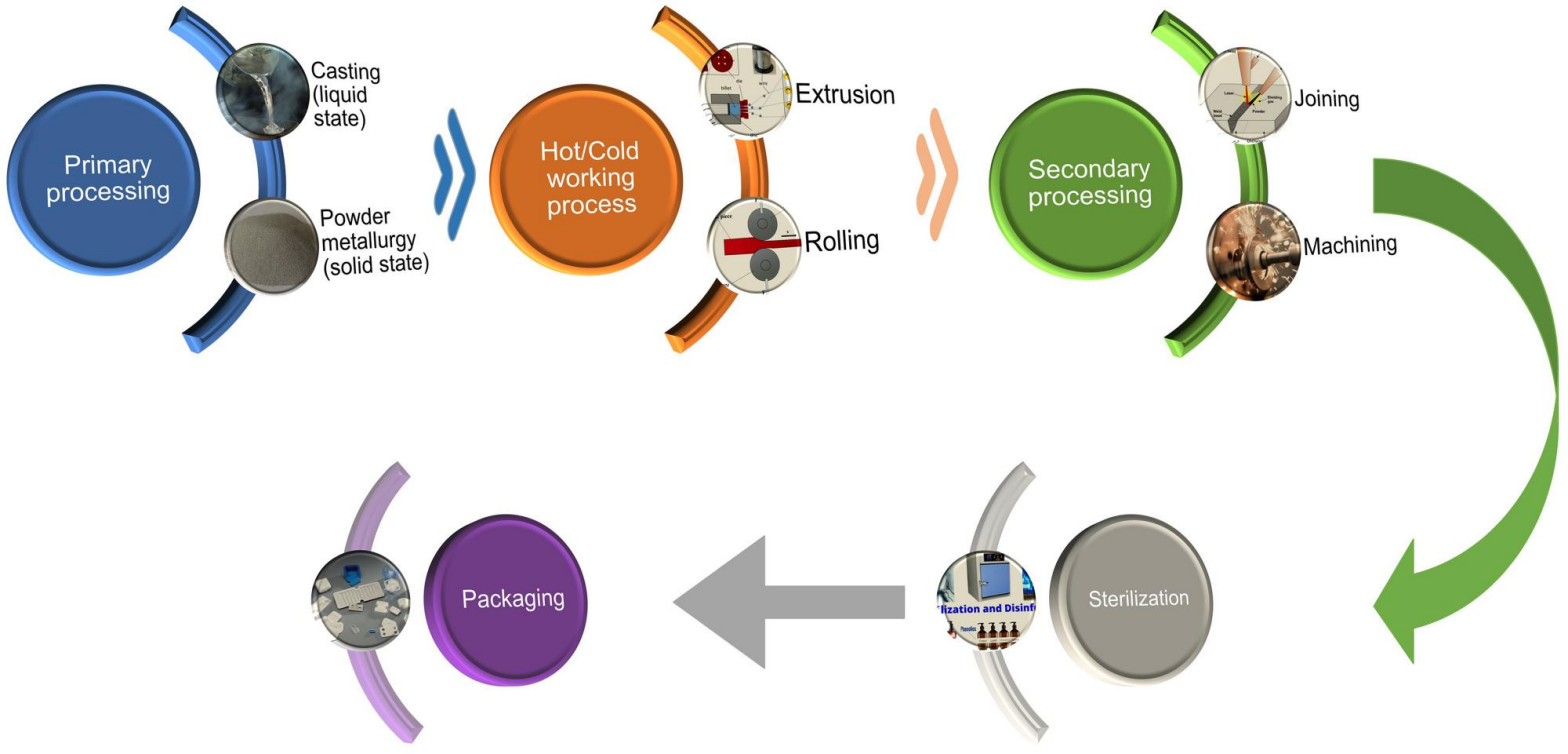
Flowchart diagram of conventional processing steps in the case of Mg-based implants.

The conventional technologies include primary and secondary manufacturing methods. Figure 7 depicts the different phases of the process, highlighting that primary manufacturing technology can be categorized into either the solid (powder metallurgy (PM)) or liquid (casting) state methods.

##### Primary manufacturing methods

Casting is a cost-effective method that creates the initial shape of an implant. Additional processes like joining or machining are then used to achieve the final desired shape for the implant. [152, 153]. The main advantages of casting are simple controlling of the material composition, low costs, and user-friendly operating systems. The classical casting procedure consists of heating a metal piece at a temperature higher than the material melting point, pouring the liquid phase into molds with different shapes, and followed by a natural solidification process. Due to the fact that Mg exhibits a pronounced oxidative character, a protective atmosphere such as argon must be used. An advantage of casting is that the alloying elements can be precisely controlled during the process. First, the castings undergo hot or cold working procedures to achieve the desired shape, such as rods, wires, plates and tubes. Then, secondary processes, including machining, joining, sterilization and packaging, are utilized to produce the final form of the implant.

The other primary manufacturing technology is PM, which is suitable for trauma implant and scaffold manufacture. This method is based on a pressure applied to Mg powder placed into a desired shape. The process is usually followed by a sintering step, in which fusion welding, combination and dislocation, recrystallization and diffusion phenomena occur [154, 155]. The final product exhibits an increased mass density. In order to obtain a near-net and complex shape of implants, metal injection molding can be applied. Lately, another innovative PM route, namely spark plasma sintering, was adopted, and parts with a decreased grain growth were produced [156–158]. This primary manufacturing technology is used in the fabrication of MAGNEZIX^®^ screws [159]. Supplementary, the PM approach is also suitable for porous scaffold manufacture when a pore-forming agent is used. It was reported that an improvement in cell adhesion and proliferation was obtained in the case of textured surfaces obtained after PM [159].

**Figure 8.**
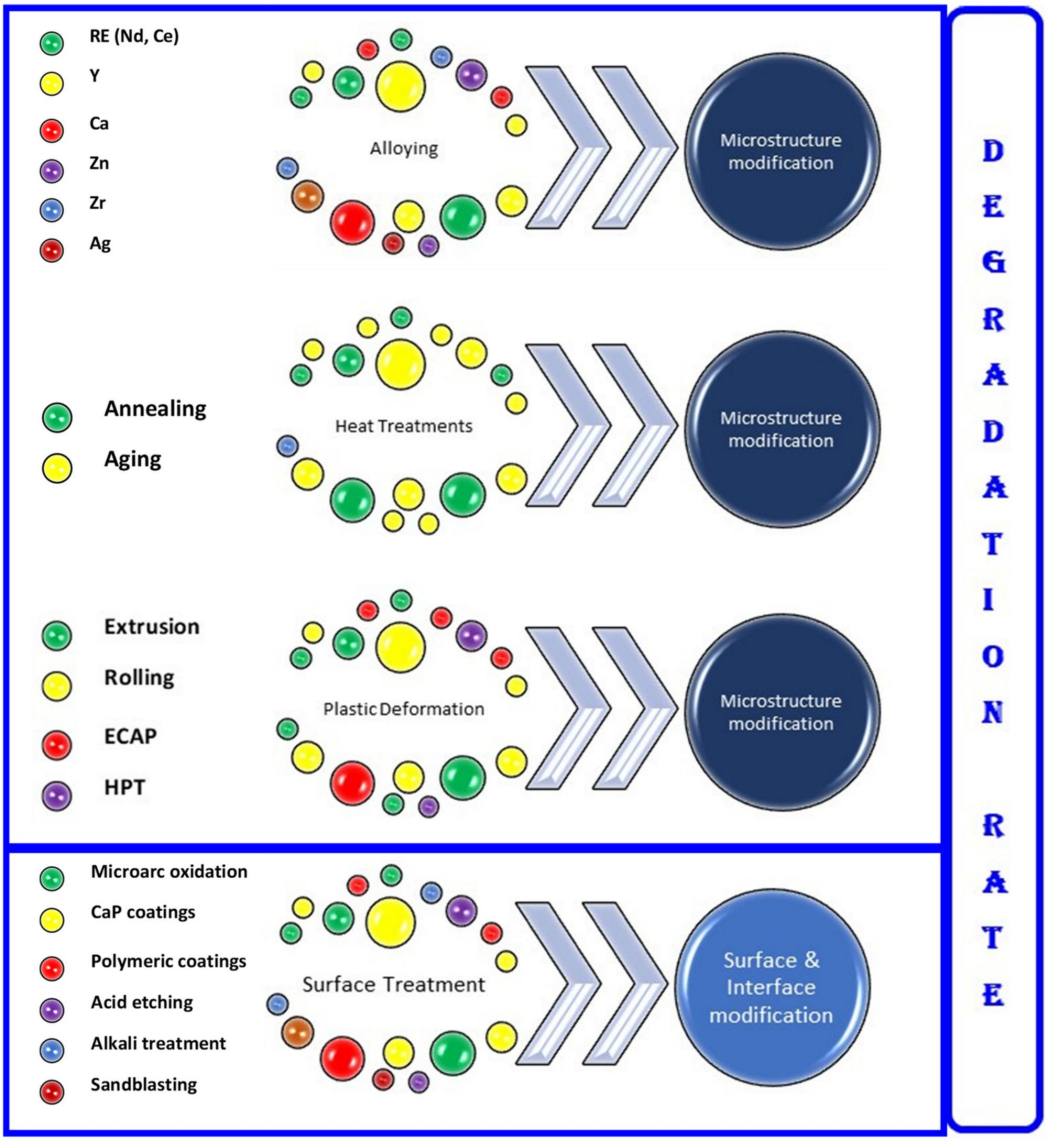
Main mechanisms used to control the speed of the Mg-based alloys’ degradation rate [47]. (Figure is licensed under CC-by 4.0.)

The intermediate step of the flowchart diagram presented in Figure 7, consisting of rolling or extrusion, has a beneficial effect on the material microstructure, strongly influencing the mechanical properties based on compressive residual stresses generation characterized by high penetration depths and proper surface finishing [160]. Also, the degradation rate is highly influenced by this procedure (Figure 8). It was found that in the case of high residual compressive stresses present in the subsurface due to deep rolling, the corrosion of the Mg-based implant was reduced by a factor of about 100.

##### Secondary manufacturing technologies

Mechanical machining is a commonly used method for creating complex geometries in Mg-based implants. However, dry machining can be problematic due to the pyrophoric effect of Mg-based alloys, which can cause chips and parts to heat up to temperatures higher than their melting point. To prevent the risk of fire, it is important to carefully monitor cutting speed and cooling gas flow rate. Literature [159] suggests that a speed of 440 r/min is suitable for abrasion and 628 r/min for polishing in dry conditions. When using computer-aided design/computer-aided machining (CAD/CAM) technology, a protective atmosphere can improve results. Mechanical machining is preferred for shaping medical implants as it ensures a well-defined surface. Recent research has investigated the influence of various factors on the machining process [158, 159].

During the manufacturing of Mg-based implants, drilling is a crucial operation. Research [161] has shown that using a 5% NaOH lubricant and a point angle of 55° results in a well-finished drilled hole. Additionally, increasing the cutting speed from 1000 to 1400 r/min leads to a decrease in the depth of the stress layer, from 40 to 25 μm. If the machining is performed in a cryogenic environment, the surface roughness and temperature decrease since the material hardness increases compared to dry machining by 87% [162].

It can be concluded that Mg-based alloys are characterized by good machinability associated with lower force consumption, good surface finishing, and easy chip formation. In order to prevent the Mg chip’s flammability, abrasive water jet machining can be used [159]. This process exhibits some advantages compared to conventional milling, as it follows reduced machining time and low stresses due to the deformation procedure.

Table 4 underlined the relationship between the manufacturing technique and the Mg-based alloy interface.

**Table 4.** Influence of processing technique on implant interfaces

Mg-based alloys	Processing technology	Conclusion	Ref.
Mg-Ca	Turning and deep rolling	Research has shown that implementing the deep rolling process after machining results in increased residual compressive stresses in the subsurface, leading to a significant reduction in corrosion rate, up to 100 times lower. Furthermore, applying the deep rolling process after the turning process has also been found to improve corrosion resistance	[160]
Mg-Al-Zn (AZ91D)	Dry milling	In order to manage the surface’s microhardness and roughness, improvements were made to the dry milling process. Taguchi with gray relational analysis was used to select an optimal process parameter setting. The feed rate was identified as the most critical parameter	[163]
Mg-Al-Zn (AZ91D)	Wire electrical discharge machining (WEDM)	A recast layer formation comprised microcracks, debris and HAZs, was noticed due to deposition and melting after the machining. This layer can be removed with the help of the WEDM method	[164]
Mg-Al-Zn (AZ31)	Cutting using TiAlN coated carbide tool	It was discovered that adjusting the cutting speed and feed rate did not significantly impact the temperature of the chip. However, it was noted that there is a direct correlation between increasing the cut depth and higher chip temperature	[165]

##### Other techniques

The wrought technique consists of a mechanical force applied to transform a bulk metal piece into a desired shaped part. The main approaches of wrought methods involved in the case of Mg-based implants are extrusion, rolling and forging. Usually, depending on the recrystallization temperature, two types of methods performed in hot and cold working conditions are used. The trauma implants are obtained after forging and hot rolling operations, and homogenized microstructures, characterized by reduced secondary phase and small grains, were obtained [166]. Another study showed that hot working conditions favored the manufacture of an implant with good mechanical properties and increased corrosion rates [167].

**Figure 9.**
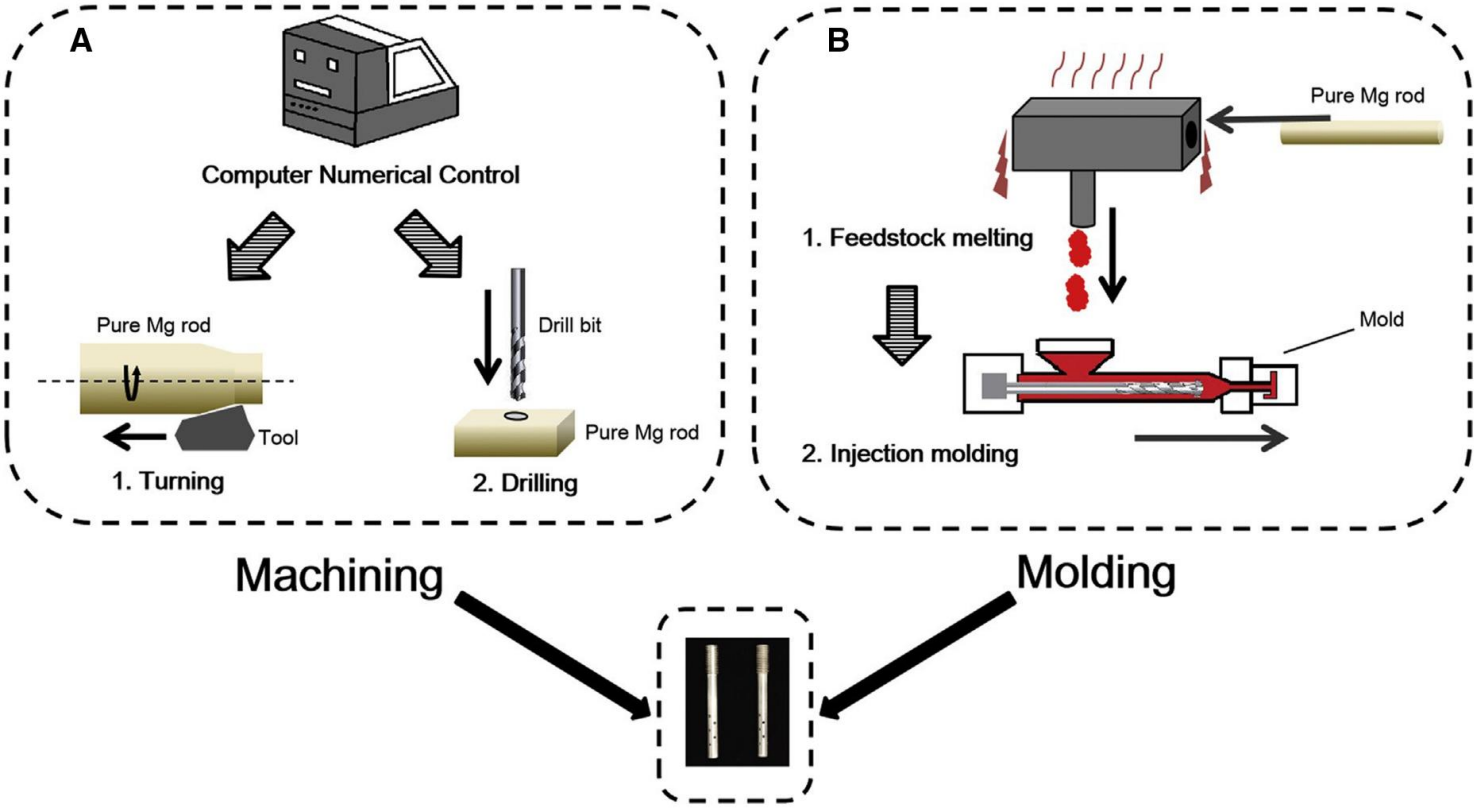
Machining (**A**) and molding (**B**) technologies used in pure Mg hip cannulated stent manufacturing [168]. Reprinted from [168] Copyright (2023), with permission from Elsevier.

Metal injection molding is a technology that creates complex-shaped implants without secondary operations. It uses metal powder and organic binder for injection molding. This technique works with tungsten, rhenium, molybdenum and niobium alloys. In terms of creating magnesium implants, metal injection molding combines slurry making and component production into one process, making it more efficient than traditional machining techniques. While the benefits of this method, such as a fine-grained microstructure and homogeneous element distribution, need to be further confirmed, it is quickly becoming a popular alternative to machining technology for creating orthopedic implants. In Ref. [168], this method was applied to develop an innovative cannulated Mg hip stent with biomaterial injection to treat incipient osteonecrosis of the femoral head by releasing Mg ions into the necrotic zones and providing mechanical support for a limited time. Paraffin was used to fill the canal of the stents to create a biocomposite model similar to what would be achieved through biomaterial injection. The part is made by melting raw material of Mg pure rods in an injection machine barrel at 600°C or higher. A mold release agent helps clear the final product. Molten Mg is injected into the mold at high speed, and after 1 min, the final product is released. The mold holes and the screw thread are created through secondary processing. Figure 9 presents a comparison between machining and molding methodologies.

**Figure 10.**
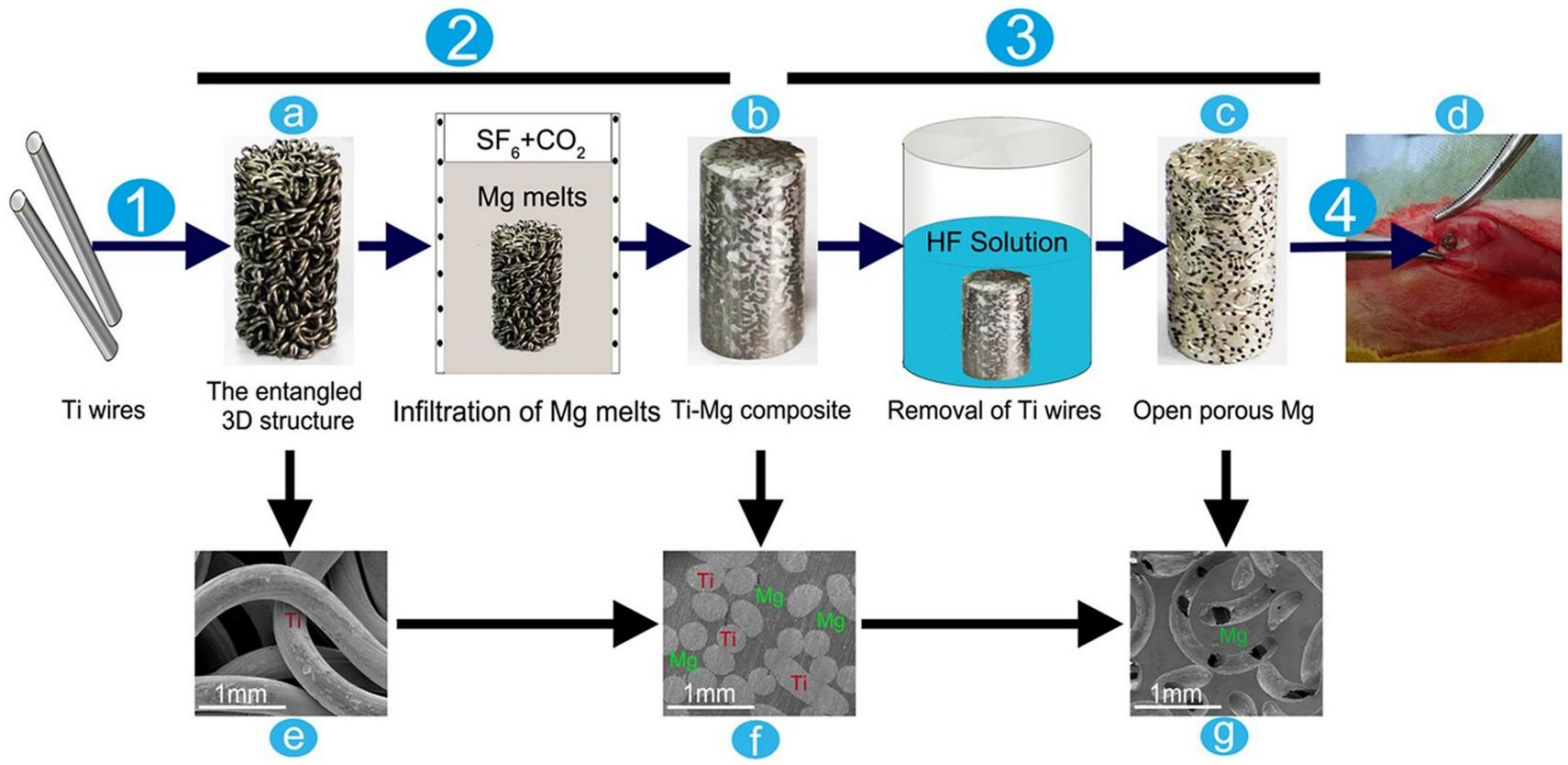
Example of TWSH technology application for the 3D Mg scaffold. (**A**, **E**) preparation of 3D titanium wire structure (step 1); (**B**, **F**) Ti-Mg composite manufacturing (step 2); (**C**, **G**) open porous Mg scaffold (step 3); (**D**) implantation of scaffolds in a rabbit animal model (step 4) [91]. Reprinted from [91] Copyright (2023), with permission from Springer Nature.

Another conventional technology for scaffold manufacture is the space holder method based on different material particles that are temporarily added to the metallic matrix powder to generate pores [169]. These particles are mixed and compacted with the metallic ones and then eliminated during or before the sintering step, leading to a porous implant structure. The following materials can be used as space holders: ammonium hydrogen carbonate [170], carbamide [171], sodium chloride [172], saccharose or sucrose [150] and polymethylmethacrylate [173]. A special type of this technology was applied for porous Mg scaffold manufacture, and it was based on a titanium wire used as a space holder (TWSH technology) [91, 174]. Firstly, a 3D shape made from Ti wires is manufactured, and then this pattern becomes a space holder, in which cast magnesium is infiltrated, leading to a Ti/Mg hybrid system. The final step involves Ti space holder dissolution at room temperature (RT) through hydrofluoric (HF) acid etching. The advantages of this method are a pipe-like porous structure and controllable pore size, while the main disadvantage is the use of HF. Figure 10 shows an example of the manufacturing process of an open-porous Mg scaffold and *in vivo* implantation.

Another method used in Mg scaffold manufacture is hydrogen injection, in which a certain amount of melted Mg is poured under advanced vacuum conditions into an alumina crucible. After that, high-pressure hydrogen is injected into the chamber. The melted Mg is heated under the effect of an inductive coil, and after the hydrogen is fully saturated, a solidification process in a water-cooled copper mold is performed. Usually, the melt is solidified only in one direction, and a straight pore structure is created as a direct consequence of the supersaturated hydrogen during the solidification procedure. The main difficulty of this method is the presence of casing equipment [175].

Regarding conventional technologies, the influence of different methods, processing parameters and implant geometry on the functional properties, surface characteristics, and implant behavior during their degradation are still analyzed for biomedical applications. A combination of *in vivo* and *in vitro* studies can be used as a database to correctly set the value of some parameters involved in mechanical processing to establish a standard procedure. An important drawback of conventional technologies is that complex-shaped implants with a porous structure cannot be fabricated through these methods. Some studies proposed the involvement of additional finishing processes, such as electrical chemical machining, electrical discharge machining, and deep rolling, to modify the Mg-based implant corrosion rate and the surface quality [160]. One of the meetest concerns linked to the machining of biomaterials is related to surface roughness and residual induced stresses, which influence the wear and corrosion resistances of the implant. The surface of the implants must be smooth, and the subsurface has to be favorable to residual stress accumulation. Due to magnesium’s pyrophoric character, the cutting speed must be carefully monitored, and the adhesion of workpiece material to the cutting tool that can generate the so-called “flank built-up” (FBU) phenomenon should be avoided. The FBU can be prohibited by using low cutting speed since the corrosion resistance of the implant can be improved through additional finishing procedure [176].

#### 3D printing technologies

**Figure 11.**
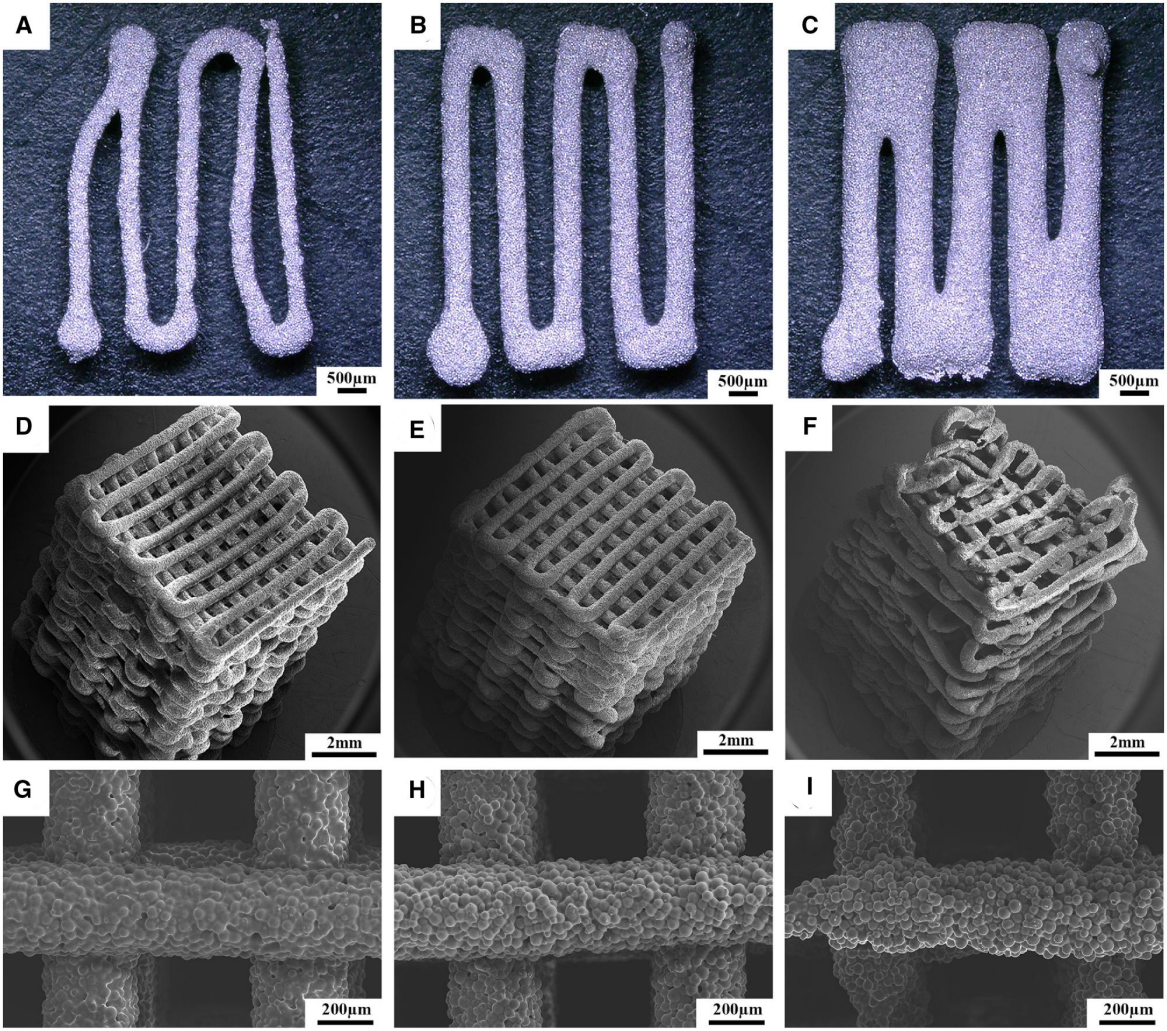
AM 3D porous Mg scaffold production step. (**A**)–(**C**) Printing a single layer strut; (**D**)–(**F**) samples exhibiting deflected surface, accurate alignment, and defects; (**G**)–(**I**) strut microstructure with 54, 58, and 62 vol.% Mg powder [177]. Reprinted from [177] Copyright (2023), with permission from Elsevier.

The production of 3D scaffolds with a regulated and controllable porous architecture is considered a challenge. It is well-known that conventional methods such as sintering, casting, space holder, or hydrogen injection can lead to an arbitrary porous structure with no control over pore size and shape and no possibility of obtaining adequate mechanical properties [144, 177]. The AM technology permits the production of high-quality implants, which exhibit a customizable architecture and shape. Based on this method, the size of the pores is controllable, and scaffolds adapted to each patient’s anatomy can be developed. Figure 11 presents an example of an AM-produced pure Mg scaffold with a porous structure.

Many studies reported adequate 3D printing technologies for Mg implants the SLM, selective laser sintering (SLS), binder-jetting and indirect AM methods [178].

SLM technology is based on an intense laser source and a CAD model that are utilized to melt and fuse the pre-spread powders layer by layer in a designated spot. A laser, an automated powder particle delivery system, a construction station, specialized software and a few other pieces of equipment constitute the system in operation. A laser diffraction tool with a galvanometer and a flattening sector lens directs the laser beam onto the building table. SLM selectively melts the powder particles layer by layer to finish the printing process of the required component, which has a 99.9% relative density [179, 180]. A dedicated software that takes into account the powder input, the stacking process, the scanning, the cooling and heating stages and the component development oversees the entire process. Using the SLM method, implants with excellent mechanical properties, high density and increased corrosion resistance can be manufactured. A narrow HAZ, which develops around the melting pool, can be an unintended consequence of the many heating and cooling cycles during SLM. The chemical composition of the alloy is influenced by this phenomenon, which can significantly alter its physical properties.

Another type of powder-based AM called SLS employs a medium-power laser to sinter powder particles, binding them together to construct solid structures. The primary distinction between the SLS and SLM techniques is that SLM uses laser scanning to achieve a complete melt, while SLS utilizes partial melting and solidification for the powder particles. SLS-processed scaffolds have poor formation quality because partially melted particles cannot completely integrate into the voids, leading to unwanted pores inside the bulk metal and struts. Hence, low densification and poor mechanical properties of SLS-processed implants and scaffolds limit their use in load-bearing orthopedic applications [181, 182].

The binder jetting technique consists of two steps. A layer of metallic powder is applied on the powder bed during the first step. The particles are bonded in a defined site via a chemical reaction or adhesion mechanism [183]. A solution that is dropped on the bonding spots determines a chemical reaction between it and the powder particles resulting in a predefined layer shape. A solid or liquid polymer is employed to attach the particles in the case of the adhesion mechanism. A curing procedure can increase the green compound’s mechanical strength [184]. A self-supported structure is created when this step is ended and can be retrieved from the powder bed. The second step is based on a debinding procedure to eliminate the unbonded powder and the binder, and post-treatment processes can be applied. The parts manufactured through this technology are characterized by a homogenous microstructure and exhibit dimensional accuracy and high surface quality [185]. As method drawbacks, it can be pointed out the low mechanical properties of the produced parts, the requirement for densification treatments, and the increased costs of binder materials [186].

In the case of the indirect AM method, the principle of infiltration technology is used. Firstly, a CAD-designed polymeric template is produced using AM technology, then it is infiltrated with NaCl paste, and the polymer is dissolved through a heating treatment [187]. Liquid Mg is cast under applied pressure inside the negative NaCl template. At the end of the process, to obtain the required Mg part, the NaCl template is removed. The main method advantage consists of the fact that the use of the Mg powder, which is characterized by explosive nature, is eliminated. The scaffolds manufactured based on this technology exhibit macroscopic geometrical sizes for struts and pores since, in the biomedical field, an important requirement states that micro and nano dimensions are adequate for cell adhesion, development, and integration [188].

Nowadays, AM is considered an important technique for 3D scaffolds with complex structures and patient anatomy-adapted shapes due to precise control over the pore size and geometry, the regularity of the designed implant, and the homogeneity of the chemical components. Much research must be undergone to introduce these types of implants for bone tissue engineering in clinical trials, although many studies reported biocompatibility, cytocompatibility and animal tests and proved the efficiency in bone defect treatment of AM-manufactured 3D scaffolds. As in the case of conventional technologies, the properties of AM scaffolds depend on process parameters such as laser input power, spot diameter, layer thickness and hatch spacing [189–191]. By optimizing these parameters, an adequate value of the scaffold density can be achieved. The post-processing treatments are important to correct unwanted effects such as increased surface roughness and porosity. In Ref. [192], PEO surface treatment enhanced the mechanical properties and reduced the degradation rate of the scaffold manufactured through SLM technology. A smooth surface is well known to be beneficial in corrosion resistance enhancement and fatigue failure prevention [193]. Unfortunately, sometimes partially melt particles attach to the surface of as-built Mg parts, and surface modification or scaffold coatings are required to control the surface roughness, mechanical properties and corrosion behavior.

## Adapted interface between Mg alloys and bone

Pure Mg and its alloys are considered emerging materials used in biomedical implant manufacture for trauma healing and bone regeneration. Many studies found significant surface challenges for Mg alloys, as it was underlined in Interface challenges of Mg-based implants section. To summarize them, firstly, it is well-known that Mg is a chemically active element with a high corrosion rate inside the human body due to Cl^−^ ions existence, so as a direct consequence, the Mg-based implants will lose their mechanical integrity and stability in a short time, favoring the premature implant failure in some cases. Secondly, pitting corrosion is a characteristic phenomenon for Mg alloys, and fracture or stress concentration can occur at the pit place. Finally, another important concern is linked to the hydrogen emission during the material degradation fact that leads to locally increased pH value of the tissue, having, as a consequence, a diminishing of its growth or hemolysis [194].

In order to solve these drawbacks, several solutions can be adopted. One of the most important is the so-called “overall optimization” that consists of alloying Mg with different chemical elements such as Zn, Zr, Al or Y in order to increase its corrosion resistance [195]. Another type of microstructure optimization can be obtained through an adequate adjustment of the processing parameters, which can lead to a refined grain structure that is beneficial to a reduced corrosion phenomenon. Supplementary, it was noticed that an amorphization process applied to the Mg-Zn-Ca alloy conducted to a metallic glass with a low corrosion rate and hydrogen emissions [196]. By applying these overall optimization procedures, it is unfortunately very difficult to control the implant’s mechanical properties and biocompatibility. The method of choice is the surface modification of Mg-based alloys that can improve the surface quality in order to ensure an increased osteointegration of the implant and to reduce its corrosion rate. To modify the material surface, different methods can be applied [47, 196]. The following sections will show the most important techniques used to tune the Mg alloy interfaces. To present them, we detail examples from the literature by combining our results with other authors’ findings. We comprehensively describe some of the surface microstructural modifications such as pulse electron beam, laser surface melting (LSM), surface mechanical attrition (SMAT), shoot peening and laser shock peening (LSP); physical deposition coating methods such as sputtering, pulsed laser deposition (PLD), dip coating, chemical vapor deposition (CVD) and spray coating; and chemical coating technologies such as acid and alkali treatment, electrochemical conversion, electrophoretic deposition (EPD) and hydrothermal treatment combined with electrodeposition. This section focuses on modifying the Mg-based alloy surface properties using the above-mentioned methods.

Table 5 presents examples of Mg-based alloys with modified or coated surfaces using different methods by underlining the control of Mg degradation and its improvement of mechanical properties [81, 197, 216]. Also, summarized the advantages and disadvantages of these related technologies.

**Table 5.** Examples of Mg-based alloys with different microstructural modifications or coating technologies emphasize the surface importance of controlling the degradation process and mechanical properties of the alloy

Type of surface microstructural modification or coating technology	Advantages	Disadvantages	Mg-based alloy/surface	Control of degradation	Mechanical properties	Ref.
*Surface microstructural modifications*						
PEB	Improved surface mechanical features and grain refinement, high degradation resistance, removal of surface impurities	Increased surface roughness, the apparition of crater defects	Mg-Al-Zn (AZ91D), Mg-Al-Mn-Zn (AM60B)/PEB surface modification	The corrosion process was investigated in NaCl medium. The PEB determined an increased Al content and fractions of intermetallic phases in the surface layer, which reduced the corrosion rate	After PEB surface treatment (30 kV, 18 pulses), the Vickers microhardness exhibited an increase, reaching a maximum value of 239 HV for AM60B and 215 HV for AZ91D	[197]
			Mg-Sn/PEB surface modification	PEB-treated samples were immersed for 12 h in 0.6 M NaCl. Localized corrosion was observed, and after 0.5 h, accelerated pitting corrosion and high H_2_ emission were reported. After 12 h, investigations showed corrosion depths of <15 μm and filiform corrosion. In comparison with extruded samples, it was concluded that the PEB improved the alloy corrosion resistance	The PEB treatment deteriorated the strength of the material and improved its ductility. The tensile yield strength was about 270.5 ± 1.2 MPa, and a tensile elongation of 11.5 ± 0.4% was measured	[198]
LSM	Increased degradation behavior, enhanced the apatite formation, increased wear resistance and good mechanical properties determined a non-porous microstructure	Increased costs, thermally induced stresses, and crack apparition	Mg-Zn-Dy/LSM surface modification	The degradation of the alloy was investigated in the HBSS. The laser processed alloys exhibited an important improvement regarding *in vitro* degradation. A maximum of 72% improvement at 45 J/mm^2^ LSM-treated samples in comparison with as-cast Mg-Zn-Dy	The average value of the hardness was increased from 73 HV (as cast) to 94 HV (LSM treated)	[199]
SMAT and shot peening	Improved wear resistance and mechanical properties, high surface residual stresses	Decreased degradation rate, contamination of the surface	Mg-Al-Zn (AZ91D)/SMAT	The corrosion rate of SMAT (2 mm balls) was the lowest in comparison with SMAT (3 and 5 mm balls)	As a function of the ball diameter used in SMAT, the Vickers microhardness was between 190 HV (2 mm balls) and 210 HV (5 mm balls)	[200]
			Mg-Al-Zn (AZ91)/shot peening	The shot peening treatment improved the corrosion resistance, with a lower value for the corrosion current density	The shot-peening treated-samples exhibited the highest surface microhardness of 64 HV_0.025_ at an impact pressure of 0.15 MPa. The microhardness gradually decreased from the treated surface to its inner bulk	[201]
LSP	Increased control over the surface morphology, improved wear resistance and surface mechanical properties, high residual compressive stresses	No important improvement in decreasing the corrosion rate, surface contamination	Mg-Al-Zn (AZ31)/LSP surface modification	The weight loss and immersion tests proved that the LSP did not influence in a high amount the corrosion process of the alloy, and increased particle or ion emissions were not noticed	The mechanical properties of the treated samples were improved. It was reported that with an increase of the hardness from 60 to 77 HV, the fatigue strength reached the value of 133 MPa for one million cycles, and regarding the friction coefficient, a constant value of 0.1 was found in the case of a force equal to 150 mN	[202]
*Physical deposition coating*						
PVD—sputtering	High coating quality, a large range of materials can be deposited, improved degradation resistance	High costs, only thin coatings are obtained, difficult to obtain complex shape coatings	Mg-Al-Zn (AZ31B)/HAp coating	The coatings deposited at 400 and 200°C had more electropositive values for corrosion potential. Lower values for the corrosion current density were reported for all the samples, and it was concluded that the coating improved in a high amount the corrosion resistance	Knoop microhardness revealed reduced value for coated samples as a function of the deposition temperature. The untreated sample exhibited a value of 76.92 ± 0.92 HK. When the temperature increased to 400°C, a reduction from 70 HK (at 23°C) to 60 HK was reported. The elastic modulus increased at 140 GPa for 400°C in comparison with 90 GPa at RT	[81]
PVD thermal evaporation and ion plating	Simple PVD process, increased corrosion resistance, high deposition rate	Thin coatings and possible delamination during long immersion time	Mg foam/Zn coatings made through thermal evaporation	The Zn-coating had a dense and smooth texture exhibiting an important interface bonding that increased the corrosion resistance	The coatings improved the elastic modulus and compressive strength by more than 70%	[203]
			Mg-Gd-Zn with Mg-Al hydrotalcite/Zr_2_ON_2_ coating deposited through arc ion plating	The polarization curves for the uncoated and coated samples were obtained in the SBF solution. It was noticed that the coated alloys had a lower current density (2.73 μA/cm^2^) and a nobler corrosion potential (−1.54 V vs. SCE)	The maximum value of the hardness of the coated samples was about 12 GPa in comparison with about 6 GPa obtained for the uncoated alloy	[204]
PVD PLD	Multi-component film coatings, high deposition rate and low temperature of the substrate, simple PVD process	Low-quality films, the feasibility of laser methods for the large zone was not proven, high costs	Mg-Ca/HAp	The HAp film deposited at 200°C exhibited the most electropositive value (−1.511 V) for the corrosion potential. The lower value of the current density (1.411 μA/cm^2^) was obtained for the HAp deposited at 300°C	In the case of film deposited at 200°C the intrinsic hardness was about 5 ± 1 GPa, and in the case of film deposited at 300°C, this physical quantity was estimated at about 9 ± 2 GPa	[83]
Dip coating	The complex and large shape of the coatings, reduced costs, high degradation resistance, high biocompatibility	Non-uniform coating with variable thickness, a time-consuming method	Mg-Ca-Mn-Zr/CA coating	The CA-coated Mg alloy had a more electropositive value (−1.60 V) and a much more electronegative value for the corrosion current density of about (4.88 μA/cm^2^)	The uncoated samples had an elastic modulus of about 42 GPa, a value that was considered very close to that of the human bone	[205]
Spin coating	Uniform coating, low coating time, reduced costs, very good degradation behavior, high biocompatibility	Difficulty in generating multi-layer coatings, limited coating area	Mg-Al-Zn (AZ31)/polyvinylpyrrolidone (PVP)/polyacryl acid (PAA) layer-by-layer assembled composite coating	The corrosion of coated samples improved by a high amount. The corrosion density current was about 3.11 μA/cm^2^, and the corrosion potential had the value of −1.40 V/SCE in comparison with the uncoated samples (*i*_corr_ = 16.3 μA/cm^2^ and *E*_corr_ = −1.5 V/SCE)	The adhesion strength was investigated based on the scratch test. The critical load of the adhesive failure of the coating/substrate system was about 3258 mN indicating a good adhesion	[206]
Electrospinning	Relatively low costs, coatings similar to the extracellular matrix, high degradation resistance, accelerated bioactivity	A high number of process variables, sample geometry and size	Mg-Ca-Zn/PCL/MgO-Ag (poly(ε-caprolactone)/magnesium oxide-silver)	The electrospun nanofiber coating was smooth, plain and porous. The corrosion test showed a good corrosion resistance for the coated sample (*i*_corr_ = 32.1 μA/cm^2^) in comparison with the uncoated alloy (184.3 μA/cm^2^)	The compression test was made before and after immersion in SBF. The compression strength for the coated sample was about 187.7 MPa after immersion compared to 172.6 MPa for the uncoated sample. Before immersion, a compression strength of 234.1 MPa was measured in the case of the uncoated sample	[207]
CVD	Very adequate for complex geometries, high degradation resistance, uniform thickness and controlled deposition	High costs, delamination, limited substrate size	Mg-Al-Zn (AZ31), Mg-Y-RE-Zr (WE43), Mg-Al-Zn (AZ91)/parylene C coating	The corrosion test indicated that parylene C has an important effect on the corrosion behavior in comparison with the uncoated samples for all the investigated alloy	The nanoindentation analysis revealed that the substrate has a higher impact on the elastic modulus than hardness (*H*). An almost constant value for *H* between 150 and 200 nm was achieved for parylene C coating. The Young’s modulus for parylene C coating is comprised in the range of 4–5 GPa, a value very similar to that of human bone	[208]
Spray coating	Many materials can be deposited with this method; this technology offers high anti-bacterial and degradation resistance	Limited possibilities to achieve a high coating thickness	Mg-Ca and Mg-Ca-Zr/ceramic coating materials such as ZrO_2_–Y_2_O_3_ and ZrO_2_–CaO	The corrosion rate for ZrO_2_–CaO coating was superior to ZrO_2_–Y_2_O_3_ coating. The polarization resistance measured for 1 h, 1 day and 7 days was between 1.6 and 2.2 Ωcm^2^ for ZrO_2_–Y_2_O_3_ and between 0.6 and 1.4 Ωcm^2^ for ZrO_2_–CaO	In the case of ZrO_2_–Y_2_O_3_ coating, Young’s modulus value was between 11 and 27 GPa. For ZrO_2_–CaO-coated samples, Young’s modulus was determined between 16 and 31 GPa	[209]
*Chemical conversion coating*						
Acid and alkali treatment	Low cost and simple technology, remove surface impurities, high degradation resistance and bioactivity, high biocompatibility	Poor quality of coatings, not adequate for a prolonged time immersion	Mg-Ca/acid treatment	*In vitro* corrosion was investigated in NaCl solution. Decreased values for corrosion current density were established at a calcium content between 0.4 and 0.8 wt%. The applied coating led to better insulation of the surface and decreased the material corrosion rate	The protective effect of MgF_2_ coating was put in evidence based on the four-point bending test under corrosion conditions. It was concluded that under a constant load, through passivation of coating, a dense Mg(OH)_2_ layer occurred. This protective layer delayed the corrosion evolution	[210]
			Mg-Al-Zn (AZ31)/alkali treatment	The uncoated and coated Mg-Al-Zn electrochemical polarization curves were obtained at 37°C in SBF solution. The coated sample exhibited a better corrosion behavior (the corrosion current density decreased from 11.8 μA/cm^2^ for the uncoated sample to 3.17 μA/cm^2^ for the alkali-treated sample). This increase in corrosion resistance is due to the Mg(OH)_2_ barrier layer induced by chemical conversion coating	Adhesion of the coating was analyzed, and it was noticed that the bonding strength of the films decreased from 57.8 MPa (alkali treatment for 3 h) to 50.2 MPa (alkali treatment for 6 h) and 47.6 MPa (alkali treatment for 12 h)	[211]
Electrochemical conversion—anodization	Increased corrosion resistance, promotes the apatite formation, high biocompatibility	High cost, non-uniform deposition, cracks apparition	Mg-Al-Zn (AZ31) and Mg-Zn-Zr (ZK60)/anodization in alkaline solution determined the presence of MgO and Mg(OH)_2_ in the anodized coating	The potentiodynamic dependencies for Mg-Al-Zn (AZ31) alloy indicated a decrease in the corrosion current density from 4.46 μA/cm^2^ (untreated) to 394.8e^−3^ μA/cm^2^ (anodized). In the case of Mg-Zn-Zr (ZK60), the *i*_corr_ shifted from 12.05 μA/cm^2^ (untreated) to 714.8e^−3^ μA/cm^2^ (anodized)	A quasi-static controlled displacement nanoindentation was performed. By analyzing the derived graphs as a function of displacement, it can be noticed that at a lower indentation depth of about 1 and 2 μm of the film, the hardness value for the Mg-Al-Zn (AZ31) (anodized) is twice lower (0.28 and 0.21 GPa) than in the case of Mg-Zn-Zr (ZK60) (anodized) (0.58 and 0.41 GPa)	[212]
Electrochemical conversion—electrodeposition	High degradation resistance, favorable for apatite formation, improved biocompatibility	Fragile coating during prolonged immersion, non-uniform coating, and variable pore size, the method is limited to substrate size and geometry	Mg-Zn-Ca/soluble CDHA coating	The corrosion behavior of the samples was investigated based on electrochemical polarization curves in SBF. The *E*_corr_ for the uncoated sample was −1645 mV, and in the case of the surface coating sample, it was equal to −1414 mV. Regarding the *i*_corr_, it was measured the value of 110 μA/cm^2^ for the bare Mg alloy, and for the coated sample, this quantity was about 25 μA/cm^2^	The mechanical properties of the Mg-Zn-Ca alloy were investigated based on SSRT tests in SBF at 37°C. Cylindrical samples were prepared. After that, they were pulled at a strain rate of 2.16 × 10^−5 ^mm/s until fracture. A similar test in the air was performed for comparison. The UTS for uncoated samples was about 144 MPa (SBF) and 162 MPa (air), and for the coated samples, it was equal to 152 MPa (SBF) and 175 MPa (air). It was concluded that the mechanical properties improved by an important amount	[213]
Electrochemical conversion—EPD	High purity deposition process, dense and uniform coating, improved corrosion resistance	EPD coatings exhibit poor adhesion, almost in all the cases the process parameters must be optimized	Mg-Al-Zn (AZ91D)/ternary HAp/chitosan (CS)/GO coating	The corrosion phenomenon was investigated based on potentiodynamic polarization plots. Different amounts of GO between 0.5 and 2 were used. It was noticed that by increasing the GO content, the corrosion resistance increased. For the HAp-CS-2GO sample, the *E*_corr_ had the most electropositive value of −1485 mV vs. SCE in comparison with all the samples (i.e. *E*_corr_ = −1565 mV for bare sample), and the most electronegative value of *i*_corr_ of 0.061 μA/cm^2^ in comparison with all the investigated samples (i.e. *i*_corr_ = −31.7 μA/cm^2^ for bare sample)	It was noticed that the elasticity modulus and hardness of the composite coatings increased from 60 ± 3.12 MPa to 8 ± 0.53 GPa, and from 40 ± 1.5 MPa to 3.1 ± 0.42 GPa	[214]
Electrochemical conversion—micro-arc oxidation	Promotes apatite formation, dense and uniform coatings, improved corrosion resistance and mechanical properties, excellent anti-bacterial property and cytocompatibility	Decreased corrosion protection during long immersion time	Mg-Zn-Zr (ZK60)/MAO coating	The corrosion rate (CR) had the lowest value of about 0.1559 g/mm^2^ h for the two-step MAO treatment because the coating is much more compact, and it prevented the penetration of the corrosive solution	The microhardness of the coated samples showed high values of about 583 HV due to the fact that the MAO coating was prepared through a special technology based on two steps (1.2–0.6 A mode). The coating was uniform and dense	[215]
Hydrothermal treatment	Low cost, thin film coatings, high degradation resistance, improved biocompatibility	Not adequate for a long time of immersion, poor quality	Mg-Al-Zn-Ca (AZ91-3Ca)/hydrothermal calcium phosphate coating	The degradation tests showed evidence that uncoated samples were severely impacted and deteriorated after soaking in SBF for 28 days. Regarding corrosion rate, it decreases from 0.19 ± 0.04 mm/year (uncoated sample) to 0.09 ± 0.01 mm/year in the case of CP7100 sample	Uniaxial compression was performed after 1, 3, 7, 14 and 28 days of sample immersion in SBF. The samples produced at pH of 7, 100°C and 3 h of hydrothermal treatment (CP7100) exhibited the highest value of compression strength (259 ± 10.65 MPa) after 14 days of SBF immersion. It was concluded that the coating was very efficient in increasing the mechanical properties	[216]

### Surface microstructural modifications

Surface microstructural modifications greatly influence the degradation and biocompatibility of Mg-based alloys. The Mg-substrate morphology can be changed by acting on the grain size and by accumulating the residual stresses in the neighborhood of the surface [217, 218]. The metallurgical modifications of the surfaces have an impact on the wettability, bioactivity and biodegradation of the Mg-based alloy.

One of the most important technologies is the pulsed electron beam (PEB) treatment, which is based on an electron beam that has melting and quenching effects, which determine metallurgical surface modification since the structure of the bulk material is not changed [219]. Regarding the corrosion properties of the material, due to the fact that the method determines the apparition of a protective passive nano-grained layer and an eruption process of the impurities, they can be improved in a high amount. Unfortunately, in some cases, after this method is applied, crater defects can appear due to the rapid melting followed by the solidification process, having, as a result, a decrease in the degradation resistance of the alloy. Morini *et al.* [197] investigated the surface properties modification of Mg-Al-Zn (AZ91D) and Mg-Al-Mn-Zn (AM60B) alloys under the effect of low energy high current PEB. They selected the acceleration voltage between 15 and 30 kV, and two settings of pulses were chosen. The low set configuration had several pulses between 2 and 16, and the high regime consisted of a number of pulses between 20 and 100. It was determined that the protective layer thickness in the first case had an average value of about 11 μm, comprised a region of 2 μm subjected to melting, solidification, and evaporation and another one characterized by a thermally affected zone of the material. The Vickers microhardness exhibited an increase after the first type of treatment was applied, reaching a maximum value of 239 HV (30 kV, 18 pulses) for AM60B and 215 HV in the case of AZ91D. A higher value was reported for the high regime conditions in the case of AZ91D (392 HV), and a lower value of 234 HV was detected for AM60B. It can be concluded that the PEB treatment greatly influenced the alloy’s mechanical properties. Regarding the corrosion process in NaCl medium, it was noticed that the second treatment determined an increased Al content and fractions of intermetallic phases in the surface layer, which hindered an accelerated degradation of the material. Lee *et al.* [198] analyzed the microstructure and corrosion resistance of an Mg_2_Sn-dispersed Mg alloy with surface modifications made through PEB. The corrosion properties were investigated based on NaCl immersion for up to 12 h. In the case of untreated samples, after 30 min was observed an accelerated H_2_ emission in developed corrosion pits since the treated samples did not exhibit such a behavior. After 12 h, the treated material was characterized by corrosion depths of <15 μm, while for the untreated material, a value of 80 μm was determined. The authors have concluded that grain refinement and impurities removal are also important to form a highly protective layer. There are just a few studies in the literature regarding this surface modification method, and advanced *in vivo* and *in vitro* studies are necessary to underline the effect of PEB treatment on the mechanical properties and degradation of Mg-based alloys.

The LSM treatment determines the generation of a microstructure with a non-porous cellular/dendritic aspect characterized by intermetallic phases placed along the grain boundaries that act as corrosion barriers. The fine-grain boundaries become nucleation zones for the biomineralization process. This method is useful when a high control of the surface wettability is necessary. Wu *et al.* [220] determined the effect of the LSM procedure on the microstructure and surface morphology of Mg-Al-Zn (AZ31B) alloy. A prismatic crystallographic texture was put in evidence along the axis perpendicular to the sample surface due to the intense temperature that resulted during the applied treatment. The grain growth in the *Z*-axis direction determined the formation of a cellular/dendritic microstructure, which presented a uniform distribution of the Mg_17_Al_12_ phase. Based on immersion tests in SBF, improved corrosion resistance and wettability of the surface were evidenced. The contact angle decreased as a function of the applied processing conditions. In the case of the maximum values for the laser fluence, a high value for the surface roughness (3.95 μm—3.18 J/mm^2^) and a low value for the contact angle (43°—3.18 J/mm^2^) were reported. Regarding the corrosion resistance, the weight loss increased from 4 to 8 mm/year for the untreated samples, and for the laser-treated samples, the corrosion rate decreased from 2.5 to 0.5 mm/year. Additional immersion tests showed a mineral HAp layer formation, improving the treated alloy corrosion resistance. Guo *et al.* [221] investigated the effect of grain refinement by laser surface treatment on Mg-Al-Zn (AZ91D) alloy regarding cell adhesion behavior. After the LSM treatment was applied, the surface roughness increased from an average of 0.16–2.53 μm. The surface energy variation was achieved from 23.1 mJ/m^2^ (untreated samples) to 42.9 mJ/m^2^ (laser-treated samples). Supplementary, good cytocompatibility, cell adhesion, and proliferation were observed, and it was concluded that LSM treatment is adequate to increase the biocompatibility of the Mg-based implants. Rakesh *et al.* [199] modified the surface of Mg-Zn-Dy alloy by melting treatment. The melted region of the surface was characterized by fine grains since columnar grains were present near the liquid-solid zone. It was noticed that the Vickers microhardness for the laser-treated samples increased 2-fold. The authors concluded that the degradation behavior and the mechanical properties improved significantly due to microstructural refinement generated by the rapid heating-cooling cycle of the melted zone. It can be noticed that laser parameter control is essential in the microstructure evolution and has a great influence on surface wettability and roughness.

From the mechanical surface modification methods, two technologies are used: surface SMAT and shot peening. Through SMAT, significant plastic deformation is generated into the Mg alloy surface, determining the apparition of high compressive residual stresses with no influence on the bulk material microstructure. Usually, this process occurs under the effect of flying balls and has, as a result, a hard nanocrystalline layer made by a dynamic recrystallization process and sub-grain formation. Chen *et al.* [222] studied the effect of SMAT on Mg-Al-Zn (AZ31B) corrosion fatigue. The hydrogen evolution tests showed that SMAT treatment increased the corrosion rate of the treated alloy due to the influence of different factors such as surface roughness, defect density, residual stress and microstrain. The surface roughness increased in a high amount after the treatment was performed, favoring a better cell adhesion but exhibiting at the same time a negative influence on the corrosion behavior. It is well-known that a smooth surface is beneficial to form a passive oxide layer. The electrochemical corrosion tests sustained the idea that SMAT led to an increase in the corrosion phenomena, and the authors concluded that larger diameter balls produced high values of mechanical stresses that negatively influenced the surface characteristics. Similar studies [223, 224] showed that SMAT must be used only as a pre-treatment solution and be combined with a surface coating method. In the case of shoot peening, a similar procedure to SMAT is applied but involves balls with lower diameter values. Yao *et al.* [201] used the shot peening method as surface pre-treatment on Mg-Al-Zn (AZ91) combined with a Zn layer coating procedure. The alloy’s mechanical properties, such as microhardness, were improved and also an increased corrosion resistance was noticed. The material weight loss was reduced by 10 times for the treated sample. When shot peening is not combined with a coating technology, an increase in the corrosion rate is observed because this method is linked to high-roughness surfaces. Peral *et al.* [225] investigated the effect of shot peening at room and high temperatures on the Mg-Al-Zn (AZ31) alloy microstructure. In both cases, a refined microstructure was obtained, but regarding the microhardness tests, better values were found for the RT treatment (hardened depth of about 350 μm). It was noticed that the hardening effect dropped and disappeared at 360°C, and a possible explanation could be linked to the fact that at a high temperature, an equilibrium occurred between the softening generated by the high temperature and the hardening effect due to the shot peening procedure. The corrosion rate was also investigated in the Ringer Lactato solution, and it was observed a high corrosion rate for the treated samples. The authors concluded that the high surface roughness negatively influenced the corrosion behavior of the material.

The LSP method has some advantages compared to the classical shot peening. LSP offers increased control over surface morphology, wear resistance and good mechanical properties. Zhang *et al.* [202] made a study regarding the effects of LSP technology on the biocompatibility and mechanical behavior of Mg-Al-Zn (AZ31) alloy. By applying this treatment, the surface roughness increased from 0.12 to 0.36 μm. Also, following the X-ray diffraction spectroscopy investigations, a twinning-generated grain reorientation to the {0002} plane was evidenced. The mechanical properties of the treated samples were improved. It was reported that with an increase of the hardness from 60 to 77 HV, the fatigue strength reached the value of 133 MPa for one million cycles, and regarding the friction coefficient, a constant value of 0.1 was found in the case of a force equal to 150 mN. This fact can be explained based on the hardening effect of the surface induced through the LSP method and the deep oxidation layer that appeared after the treatment was applied. The weight loss and immersion tests proved that the LSP did not influence in a high amount the corrosion process of the alloy, and increased particle or ion emissions were not noticed. The cytocompatibility tests on human adipose-derived stem cells led to comparable results obtained in the case of untreated samples. Guo *et al.* [226] investigated the effect of LSP on the corrosion behavior of Mg-Zn-Zr (ZK60) alloy. They observed by analyzing the material microstructure, surface roughness and the residual stresses, which occurred after the treatment, that high compressive stresses were induced and were directly linked to an increase of the corrosion rate by 52.1%. In conclusion, by increasing the laser power density, the plastic deformation can be tuned, and the surface roughness can be increased from 0.2 to 6.11 μm. A refined grain structure with an average dimension of 17 μm was found. The LSP-treated alloy evidenced a reduced corrosion crack phenomenon. Unfortunately, this surface metallurgical modification can induce surface contamination, and no noteworthy improvement of degradation properties or biocompatibility was reported in the literature.

It can be noticed that surface metallurgical modification methods are suitable for tailoring the subsurface and surface microstructure. Other methods’ disadvantages, such as instability of the formed layer or low coating adhesion, can be overcome based on these techniques.

### Physical deposition coating

The physical deposition coating method is applied to develop, through physical technologies, a secondary layer on an Mg-based substrate to obtain increased control over the alloy biocompatibility and corrosion rate. Compared with the conversion coatings, the adhesion between the substrate and the coating is reduced. The main physical deposition coating methods are physical vapor deposition (PVD), CVD, dip coating, thermal spray coating, spin coating, electrospinning and sol-gel coating.

PVD consists of ions or atoms deposition process onto a base substrate in order to obtain uniform, adhesive, and high-quality coatings. One of the most used PVD methods found in literature is sputtering technology. Bita *et al.* [227] investigated the adhesion process of bioceramics such as HAp or BG on Mg-Ca alloys. For the coating deposition, a magnetron sputtering (MS) system that had a radio frequency (RF) generator was used. This equipment worked in a high-purity argon atmosphere, and a deposition rate of about 8 and 5.5 nm/min was achieved for the HAp and BG layers, respectively. The morphological features of the coatings were analyzed based on SEM images. It was noticed that a rougher surface characteristic is present in the case of HAp coatings since, in the case of BG, a finer microstructure was evidenced. Atomic force microscopy (AFM) was used to evaluate the roughness parameters. The arithmetic average of absolute values (*R*_a_) was found to be 106 nm for the HAp coating and 72 nm for the BG layer. In the case of HAp-coated samples, an air annealing treatment at 500°C/1 h was applied to generate a crystalline layer with a thickness of 1 μm. The cohesion/adhesion pull-out values were higher than 15 MPa, as it was indicated in the ISO 13779-2 standard. This fact proved that the developed coatings exhibited adequate performance and could be successfully applied in load-bearing implant manufacture.

**Figure 12.**
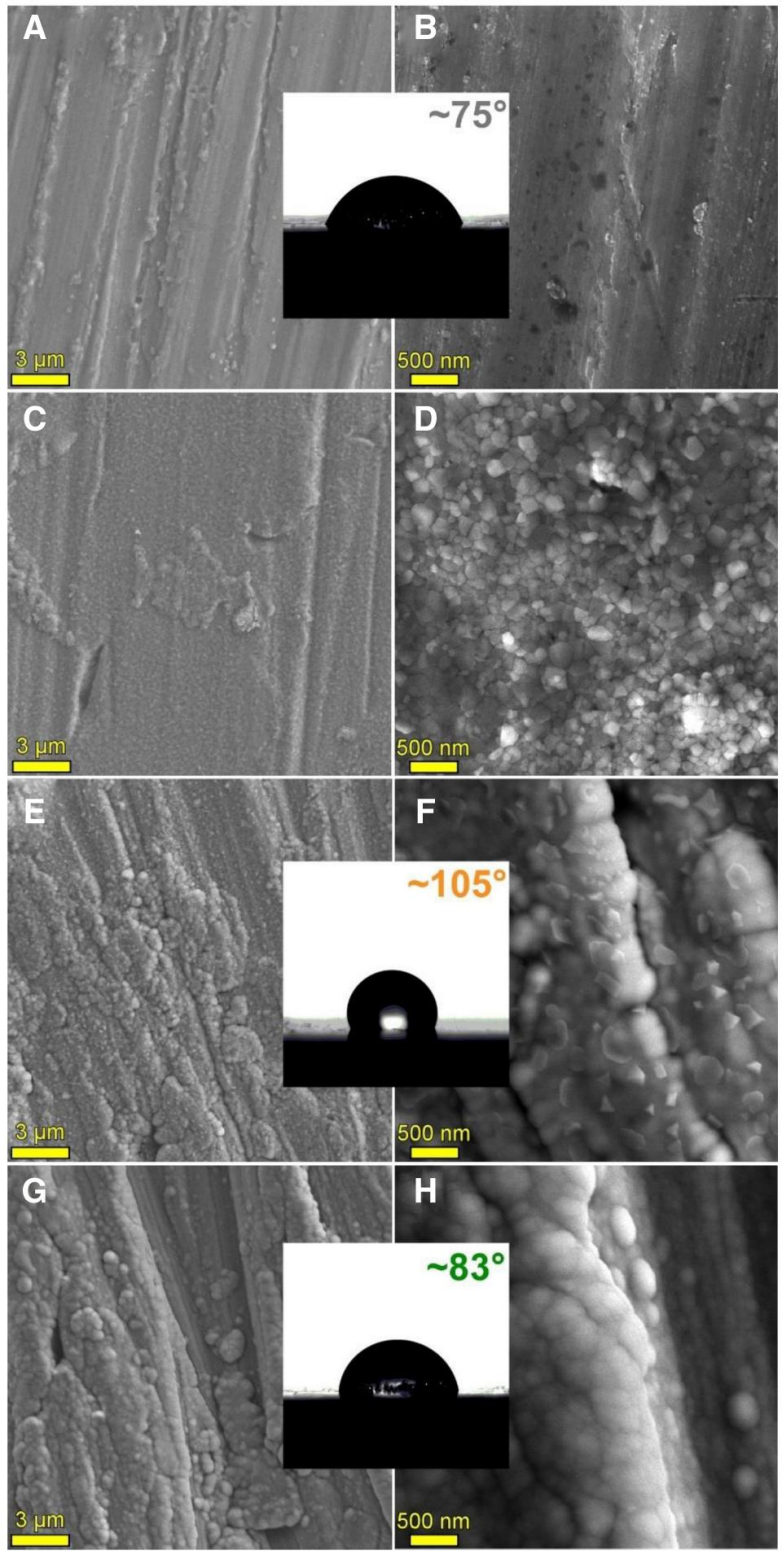
SEM Images and wettability investigations of (**A**, **B**) uncoated Mg-Ca alloy; (**C**, **D**) uncoated Mg-Ca alloy annealed in air at 500°C; (**E**, **F**) HA2-coated samples; (**G**, **H**) BG2-coated samples [228]. (Figure is licensed under CC-by 4.0.)

The electrochemical and *in vitro* biological response analysis of crystallized HAp and silica-rich glass coatings on Mg-Ca alloy was performed in [228]. The coatings were deposited with a RF-MS system, including a Vacma UVN-75R1 device coupled with a 1.78-MHz generator in an inert argon atmosphere. Coatings with a thickness of 1 μm were obtained, and as mentioned previously in [227], for the HAp-coated samples (HA2), a supplementary heat recrystallization treatment was used. Regarding the BG coating (BG2), an increased silica content was obtained using fused silica coupons placed on the magnetron cathode target. It was performed an extended physical-chemical analysis that included the surface and cross-sectional morphology, surface wettability, crystalline status/phase composition and chemical structure investigations. From the SEM images, it was noticed that both types of coatings exhibited agglomerates, and polyhedral-shaped grains with sizes between 150 and 350 nm were put in evidence only in the case of HAp coating. Regarding the wettability, the two coatings increased the contact angle, reaching values of about 83° for the BG coating and 105° in the case of HAp (Figure 12).

**Figure 13.**
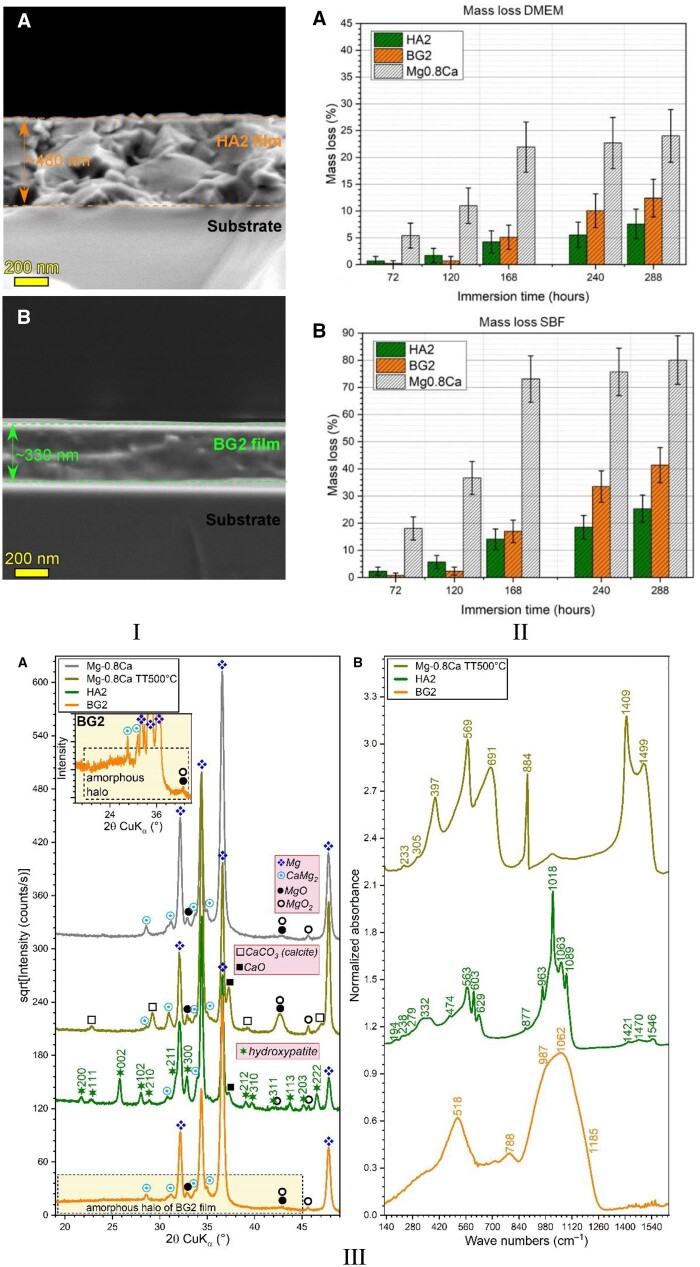
Experimental results obtained for Mg-Ca alloys coated with HAp and bio-glass. (**I**) SEM images of the cross-sectional morphology of HAp (HA2) (**A**) and bio-glass (BG2) (**B**) coated samples; (**II**) mass loss in DMEM (**A**) and SBF (**B**) for uncoated and coated samples; (**III**) XRD pattern (a) and FTIR spectra (b) of bare Mg-Ca, bare heat thermal treat at 500°C Mg-Ca, HAp-coated alloy and bio-glass-coated alloy [228]. (Figure is licensed under CC-by 4.0.)

Based on the X-ray diffraction collected in grazing incidence, diffraction peaks characteristic for MgO and MgO_2_ did not present a modification for HAp-coated samples in comparison to the uncoated ones. It can be concluded that the HAp layer is a compact one. Regarding the BG coating, an amorphous state was evidenced. The FTIR analysis showed that for HAp film, split and sharp IR absorption bands, which characterized the orthophosphate vibrational mode, occurred. In the case of BG-coated samples, the amorphous state of the deposited layer was once again confirmed. Some results are presented in Figure 13. Functional performance tests included hydrogen release, weight loss measurements and electrochemical behavior analysis. The HAp-coated sample exhibited a low weight loss and hydrogen release compared to the BG-coated alloys and bare Mg-Ca material. The corrosion tests revealed that in the case of SBF electrolyte, the coated samples had more electropositive corrosion potentials (i.e. −1.69 V for HAp; −1.75 V for BG) compared with the uncoated material (−1.86 V) proving an increased corrosion resistance. This observation is in good accordance with the mass loss results. The material and RF-MS coatings’ cytocompatibility was investigated by analyzing the fibroblast and osteoblast cell viability after 24 and 72 h. For the first time interval, the BG-coated samples exhibited a decreased cell viability at 7 days of cell culturing since after 72 h, all the samples presented a decrease of fibroblast and osteoblast viability obtained at 7 days. It was concluded that the crystalline HAp coatings had better performance than bare material and BG-coated samples.

Parau *et al.* [81] investigated the deposition temperature effect on magnetron-sputtered HAp coatings on Mg-Al-Zn (AZ31B) substrate. The coatings were prepared based on the above-mentioned physical deposition method at different temperatures ranging from RT to 400°C. Regarding the microstructure, an increased grain size directly proportional to the temperature was reported. The coated and uncoated sample corrosion rate was investigated, and it was concluded that the highest corrosion resistance was obtained at a sputtering temperature of 200°C. It was concluded that the hardness value decreased with the temperature increase. The authors noticed that the HAp coatings exhibited good anticorrosive and mechanical properties of the magnetron sputtering made at a temperature lower than 200°C because if this value is exceeded, a diminishing of the properties is observed, and the coatings become unnecessary.

**Figure 14.**
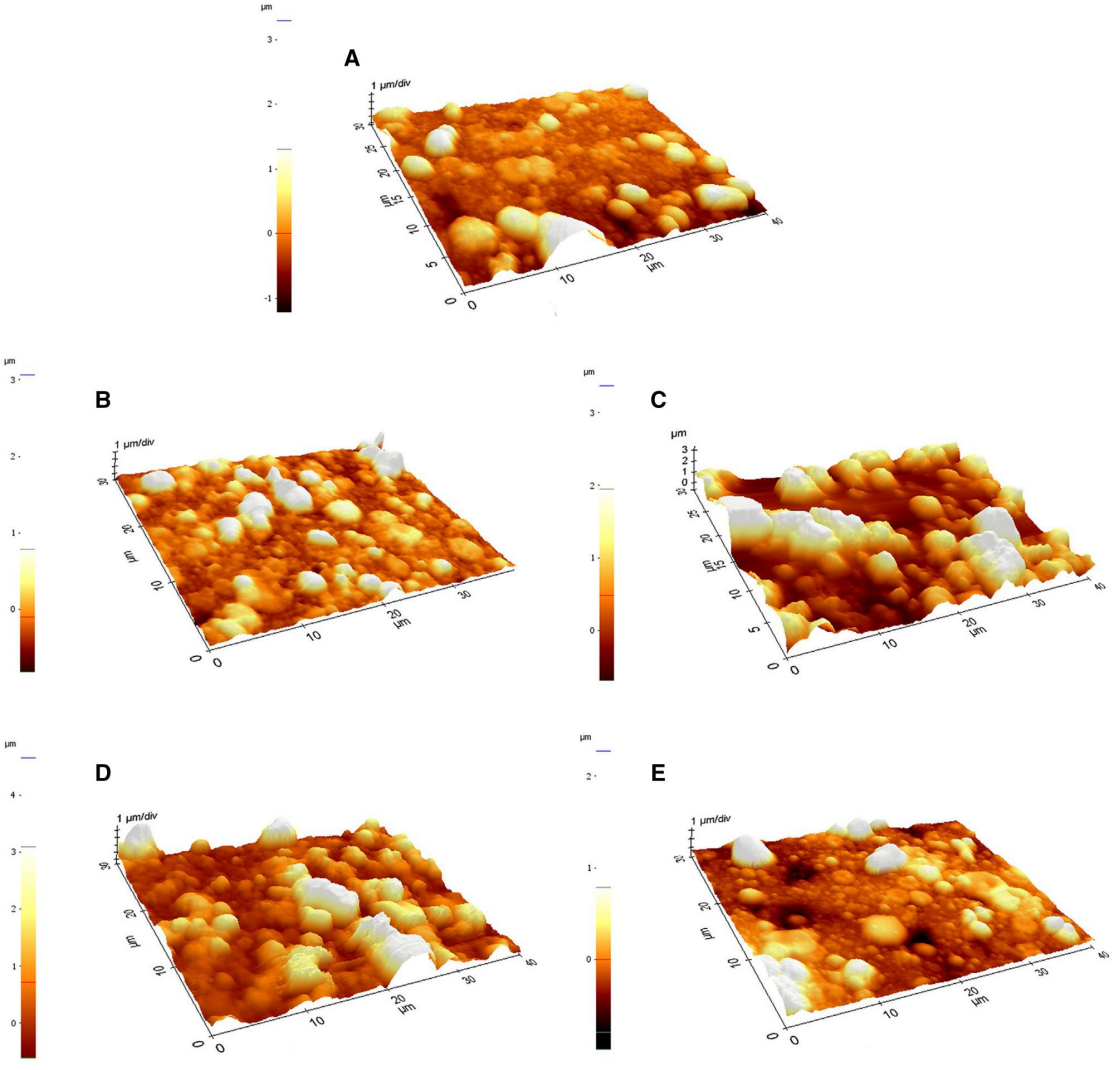
AFM images of surface topology with HAp films deposited through PLD at (**A**) RT, (**B**) 100°C, (**C**) 200°C, (**D**) 300°C, (**E**) 400°C [83]. Reprinted from [83] Copyright (2023), with permission from Elsevier.

PLD is another PVD technique in which a laser beam is directed to deposit a target material on a given substrate in a vacuum chamber. The target material is vaporized and deposited as a thin film, and high-quality coatings are obtained. Rau *et al.* [83] deposited HAp coatings on Mg-Ca through the PLD method and investigated the corrosion resistance of the alloy in an SBF medium. The PLD setup was comprised a stainless-steel deposition room equipped with a high vacuum pump system. An Nd:YAG laser with four harmonics was involved. The working temperature varied from 25°C to 400°C, and the deposition process occurred in vacuum conditions (10^−5^ mbar). The thickness of the HAp layer was comprised between 2.3 ± 0.4 μm at RT and 2.4 ± 0.4 μm at 200°C until 1.6 ± 0.3 at 400°C. A granulated morphology of the surface characterized the layer deposited at 200°C. It was observed that between 200°C and 300°C crystalline state of HAp can be achieved, since between RT and 100°C and above 400°C amorphous state and dissolution effect of the HAP are reported. The authors concluded that the best deposition temperature must be comprised between 200°C and 300°C. These samples exhibited a hardness between 5 and 9 GPa since the average surface roughness was found to be 619 nm at 200°C and 744 nm at 300°C. Figure 14 presents the surface topology investigated through AFM. Regarding the corrosion investigations, the lowest value for the current density was equal to 1.411 μA/cm^2^ in SBF solution deposited at 300°C and at 200°C a value of 1.612 μA/cm^2^ was obtained. In conclusion, it was recommended that the deposition temperature be set at the interval mentioned above to manufacture a high-quality coating for medical implants.

**Figure 15.**
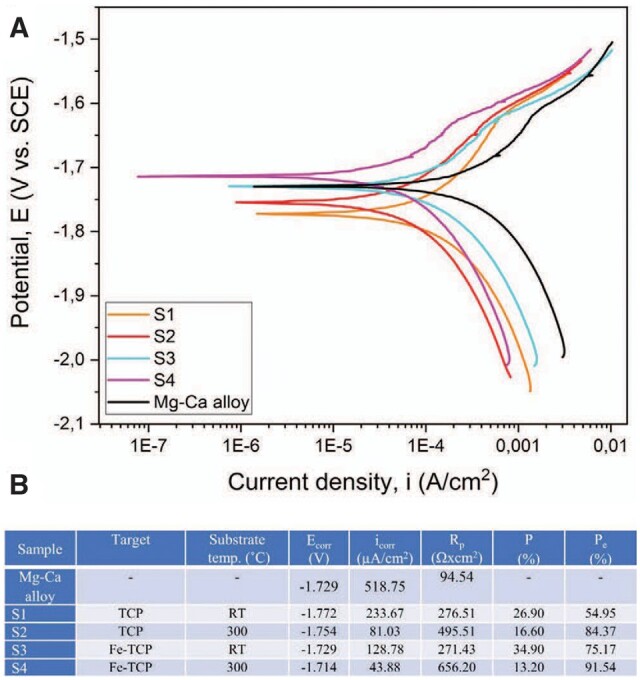
(**A**) Tafel curves for the S1—Mg-Ca coated with TCP at RT; S2—Mg-Ca coated with TCP at 300°C; S3—Mg-Ca coated with Fe-TCP at RT; S4—Mg-Ca coated with Fe-TCP at 300°C; (**B**) Important electrochemical parameters obtained in SBF electrolyte [84]. Reprinted from [84] Copyright (2023), with permission from Wiley.

**Figure 16.**
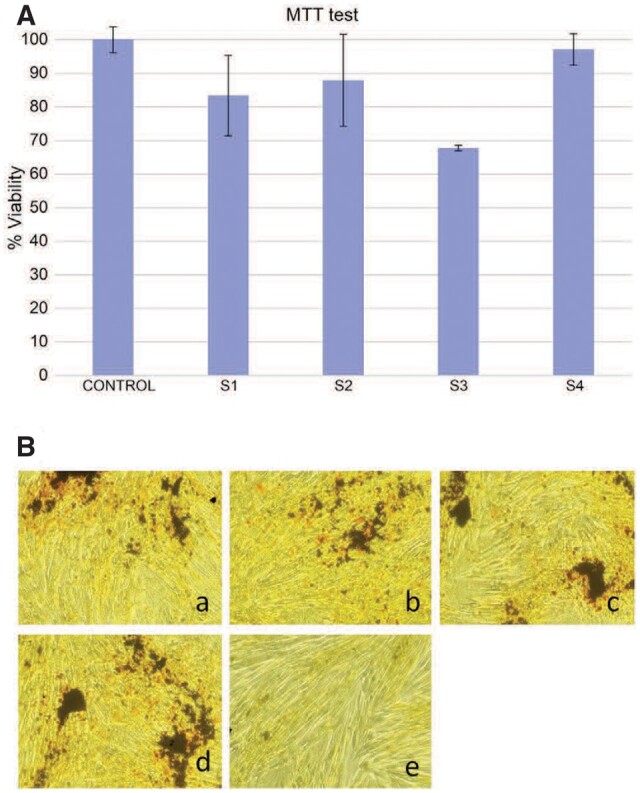
(**A**) MTT Test for S1–S4 samples. The control is represented by cells grown in the absence of the S1–S4 samples. (**B**) Osteogenic differentiation of cell with S1–S4 extracts (**a**—S1; **b**—S2; **c**—S3; **d**—S4; **e**—control) [84]. Reprinted from Ref. [84] Copyright (2023), with permission from Wiley.

Antoniac *et al.* [84] developed iron-substituted tricalcium phosphate (Fe-TCP) coatings deposited through PLD on Mg-Ca alloys in order to improve corrosion behavior. The main components of the PLD chamber were a holder heater, a vacuum pump and a target holder rotation-translation setup. Based on an Nd:YAG laser, thin TCP and Fe-TCP films were deposited at a laser wavelength of 355 nm and fluence of 2 J/cm^2^. All the coatings were characterized by a thickness of 2 μm ± 5%, and the deposition rate for TCP was set at 0.17 nm/pulse, since for the Fe-TCP, it was about 0.34 nm/pulse. The corrosion rate of the coated and uncoated samples was investigated in the SBF electrolyte. The most electropositive value for the corrosion potential was obtained in the case of Mg-Ca alloy with Fe-TCP coating deposited at 300°C. This sample exhibited the lowest value of 43.88 μA/cm^2^ for the corrosion current density in the SBF medium, a fact that underlined a good corrosion resistance compared with other samples. Increased corrosion resistance was put in evidence for the Fe-TCP coating deposited at RT on the Mg-Ca substrate (Figure 15). The MTT assay was used to investigate the material cytotoxicity. Again, the best cell viability of about 97.0% compared to the control sample was achieved for the Fe-TCP coating deposited at 300°C. This type of coating is characterized by an increased amorphous phase content, it is much more soluble than the other coatings, and the iron ions manifested a benefic effect on cell proliferation (Figure 16). There were also microbiology tests performed. The inhibition of *Escherichia coli* bacterial growth was determined in the case of all samples since no significant effect was put in evidence against *S.aureus*. The authors concluded that the Fe-TCP coating deposited through the PLD method at 300°C is adequate for Mg-based implants due to the fact that iron presence modifies the coated layer morphology and increases the corrosion resistance.

Rau *et al.* [229] deposited RKKP (the name initials for A. Ravaglioli, A. Krajewski, M. Kirsch, A. Piancastelli) glass-ceramic material containing wollastonite and HAp phases on Mg-Ca substrate based on PLD technology. The film deposition procedure was conducted in a stainless-steel chamber through laser ablation at 4 × 10^−4 ^Pa pressure. The laser source made an ablation based on a second harmonic at 532 nm at a 10 Hz repetition rate and a pulse length of 7 ns. The structure and morphological characterization evidenced a 100-μm thick, compact, and dense film with an average surface roughness of about 295 ± 30 nm. The electrochemical corrosion tests were made based on the linear polarization technique, and it was concluded that the RKKP coating improved the corrosion resistance of the Mg-Ca alloys in the SBF solution.

**Figure 17.**
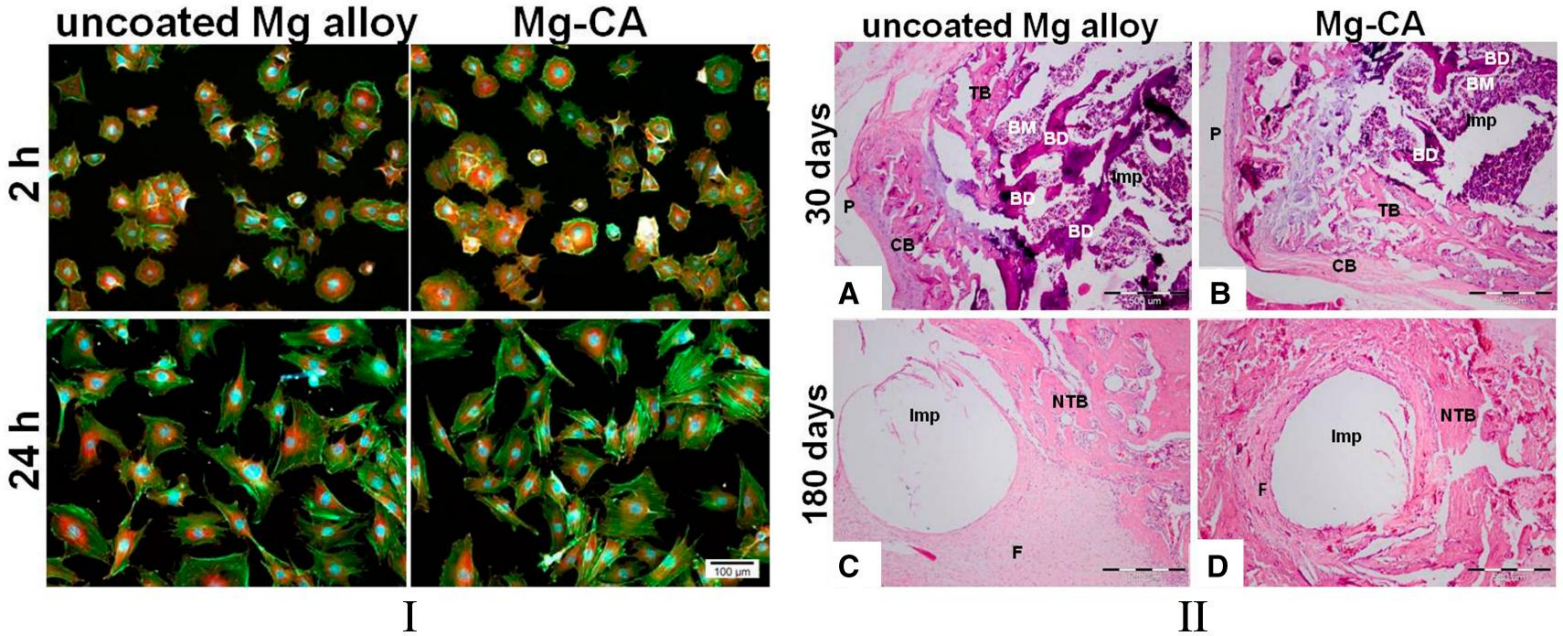
*In vitro* and *in vivo* tests. (**I**) Fluorescent images of MC3T3-E1 cells grown in culture media for uncoated and coated Mg-Ca. (**II**) Hematoxylin and eosin staining of bone sections of the rat implanted with uncoated (**A**, **C**) and CA-coated (**B**, **D**) samples. (BM, bone marrow; TB, trabecular bone; BD, bone destruction; CB, compact bone; imp, intra-medullary implant site; P, periposteum; NTB, newly formed trabecular bone) [205]. (Figure is licensed under CC-by 4.0.)

Dip coating is a physical deposition method adequate for samples with large geometrical dimensions and complex shapes. This technology permits the generation of ceramic or polymeric layers to sustain biomineralization and control the degradation of the substrate. Neacsu *et al.* [205] performed a complete material characterization and *in vitro* and *in vivo* studies of cellulose acetate (CA)-coating on Mg-Ca-Mn-Zr alloy through the dip coating technique. The synthetic polymeric coating formation was investigated based on FTIR and SEM. The coated samples exhibited a smooth surface because the compact polymeric layer with a thickness of 90 μm had pores with a small diameter. This porosity was induced by the solvent molecules that evaporated and conducted to the channel formation. The FTIR spectra put in evidence a clear difference between the coated and uncoated samples and sustained the CA layer presence on the material surface. From the electrochemical corrosion analysis, it was concluded that the CA-coated material exhibited a higher electropositive corrosion potential of about −1.60 V and a lower corrosion current density of 4.88 μA/cm^2^ compared with the uncoated samples (−1.85 V, 497.96 μA/cm^2^). The *in vitro* tests were performed on MC3T3-E1 mouse pre-osteoblast cells. It was observed that a positive evolution of the cell viability over a time of 5 days. Mg-based implants were inserted into a rat femur with no complications and following the standard protocol. The histological analysis of the implantation sites at 90 and 180 days after the surgery proved new bone formation around the implant. In the case of CA-coated samples, a reduced quantity of fibrous tissue was present at the boundary between bone and implant (Figure 17). It was concluded that CA coatings on Mg substrate have great potential in the orthopedic implant market and are very efficient in bone regeneration.

**Figure 18.**
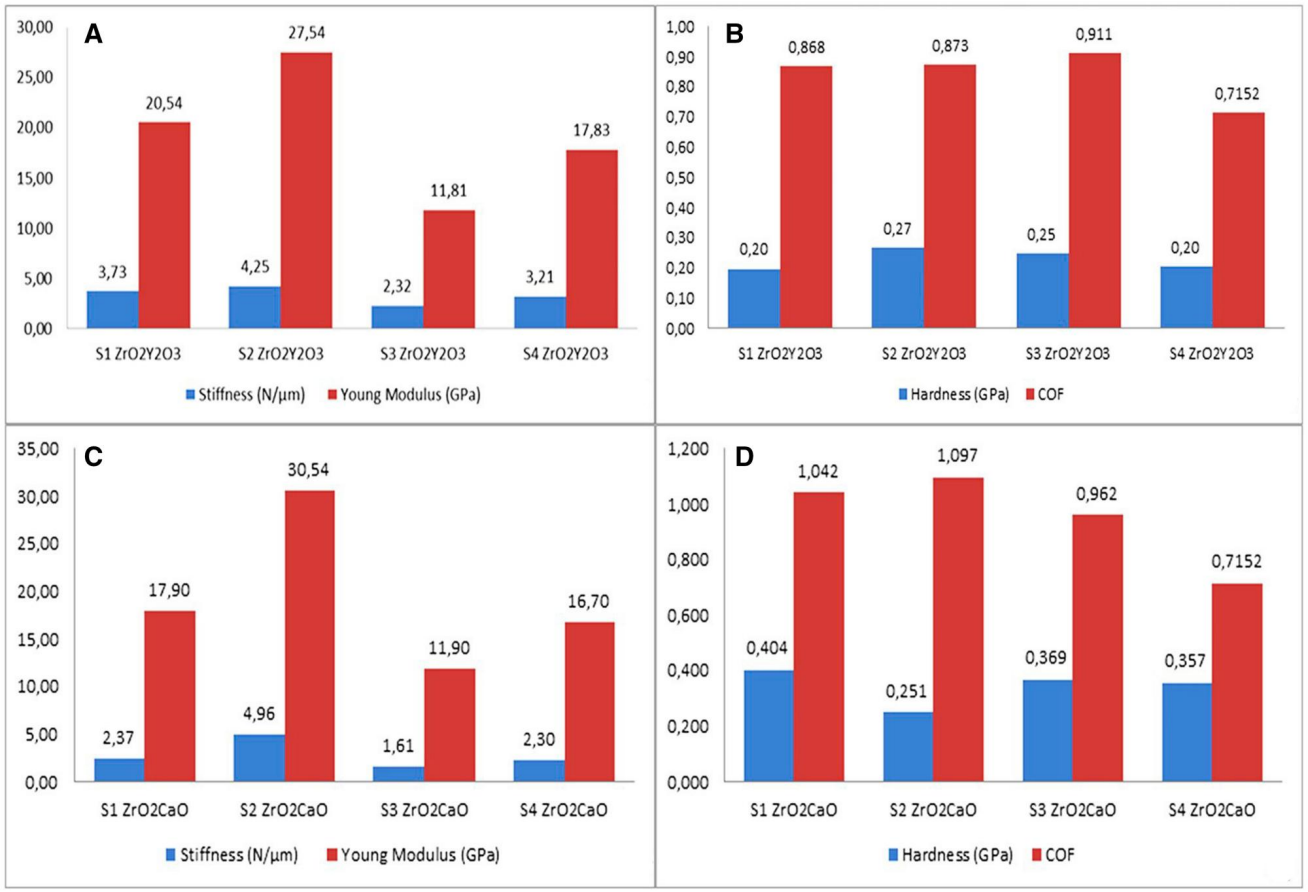
Mechanical properties determined in the case of coated investigated samples. (**A**, **C**) Stiffness and Young modulus for both types of coating; (**B**, **D**) hardness and COF for both types of coating [209]. Reprinted from [209] Copyright (2023), with permission from Elsevier.

CVD is another physical method in which volatile precursors react with the Mg substrate to generate coatings. This method is characterized by expensive equipment with a controllable deposition rate and can lead to coatings with constant thickness for variated sample shapes. Surmeneva *et al.* [208] deposited through the CVD method parylene C coating with a thickness of 2 μm on different Mg-based alloys such as Mg-Al-Zn (AZ31 and AZ91) and Mg-Y-RE-Zr (WE43). AFM, XRD and FTIR were used to investigate surface morphology and elemental composition. The AFM method proved that the parylene C coatings are smooth and uniform with an increased roughness compared to the uncoated samples. Through XRD, it was put in evidence the semicrystalline structure of the parylene C with (020) preferred orientation of the monoclinic cell. All the polymer-coated samples had higher polarization resistance in Hanks’ solution. It was concluded that in the case of saline media, parylene C is a very good coating. It determined a reduction in the corrosion process compared to the uncoated samples. The nanoindentation analysis was performed, and the coating exhibited an elastic behavior. It was concluded that this type of polymeric coating was very special and offered increased corrosion protection and modification of the surface mechanical properties. Spray coating can enhance the bioactivity and biodegradation of Mg-based alloys by applying different non-metallic and metallic coatings through thermal spray processes. Istrate *et al.* [209] prepared ZrO_2_-Y_2_O_3_ and ZrO_2_-CaO coatings through plasma jet spraying on Mg-Ca and Mg-Ca-Zr alloys. SEM images put in evidence that the ZrO_2_-Y_2_O_3_ ceramic layer exhibited columnar type grain with porous aspect and increased roughness, with inclusions, which remained unmelted during the coating procedure. In the case of ZrO_2_-CaO coating, a compact structure and increased adherence without unmelted particles were observed. The average surface roughness was found to be between 68.5 and 99.8 μm for ZrO_2_-Y_2_O_3_ film, and in the case of the ZrO_2_-CaO coating, this parameter varied between 55.4 and 69.1 μm. The corrosion potential of the samples exhibited similar values for both coatings and had values between 1269.6 and −1588.2 mV and demonstrated an increase in the corrosion resistance compared with the uncoated samples. Figure 18 presents the mechanical properties of the investigated samples (S1—Mg-1.3Ca-5.5Zr; S2—Mg-Ca; S3; Mg-0.7Ca-0.4Zr; S4—Mg-0.7Ca).

The *in vitro* tests on coated samples exhibited moderate cell viability due to the osmolality phenomenon, hydrogen release and Mg ions emission. Overall, it was found that these innovative ZrO_2_-based coatings are adequate for Mg-Ca-Zr alloys and can be considered for future implant applications.

Table 6 summarizes the main physical deposition methods with some literature examples [230, 245].

**Table 6.** Main physical deposition methods with selected literature examples

Technique	Method	Mg-based substrate	Coating	Remarks	Ref.
PVD	Sputter coating	Mg-Ca	HAp	The coating had a benefic effect on the corrosion process (the corrosion current density was reduced from 90 to 1.8 μA/cm^2^)	[230]
		Mg-Zn	TiO_2_	After the coating process, the implant surface became smoother without micro-cracks that favored the thrombosis apparition. The hemolysis ratio was reduced from 47% to 0.1%	[231]
		Mg-Gd	Mg5Gd	After the coating procedure, columnar growth was reported. The degradation rate of the alloy was reduced	[232]
	Thermal evaporation	Mg-Al-Zn (AZ31)	Mg_17_Al_12_	The corrosion resistance and degradation rate of the alloy improved	[233]
	Ion plating	Mg-Gd-Zn	Zr_2_ON_2_, Mg-Al; Mg-Al/Zr_2_ON_2_	After the coating procedure, a crack-free surface was obtained. Increased corrosion resistance and high biocompatibility were reported	[204]
	Pulse laser deposition	Mg-Ca	HAp	The best deposition temperature must be comprised between 200 and 300°C. These samples exhibited a hardness between 5 and 9 GPa since the average surface roughness was found to be 619 nm at 200°C and 744 nm at 300°C. After the coating, the corrosion resistance increased	[83]
Dip coating	–	Mg-Al-Zn (AZ31)	HF acid pre-treatment followed by dip coating in HAp silane coating	The nano HAp particles were dispersed in silane and enhanced in a high amount the corrosion resistance.	[234]
		Pure Mg	Polyurethane (PU) functionalized with polyethylene glycol (PEG)	The coating decreased the degradation rate and showed good antibacterial properties against *E.coli*	[235]
Spin coating	–	Chitosan/water glass on Ca-P	Mg-Zn	The corrosion was hindered for a long time in Hanks’ solution. A decreased hydrogen emission was reported	[236]
Sol-gel coating	–	Sol-gel derived nHAp	Mg-Al-Zn (AZ91)	The coating improved the corrosion process of the alloy and reduced in a high amount the hydrogen and Mg ion emissions.	[237]
		58S and 68S bioactive SiO_2_	Mg-Al-Zn (AZ91D)	The surface exhibited small cracks but showed an excellent biomineralization character. Increased corrosion resistance and high biocompatibility were reported.	[238]
Electrospinning	–	Electrospun polycaprolactone (PCL) and PCL combined with nano BG	Nitric acid pre-treated Mg-Al-Zn (AZ91)	After sample immersion in SBF, a cauliflower-like morphology was noticed.	[239]
		PCL/nHAP through electrospinning + dip coating	Mg-Al-Zn (AZ31)	The *in vivo* tests performed on New Zealand rabbits showed excellent bone healing and osteointegration of the implant	[240]
CVD	Atomic layer deposition	Zirconia (ZrO_2_) nanofilms	Mg-Sr	The zirconia coating significantly improved the cell viability.	[241]
		ZrO_2_ and titania (TiO_2_)	Mg-Al-Zn (AZ31)	Both coatings reduced in a high amount the corrosion rate. Zirconia exhibited a better performance in comparison with titania coating.	[242]
Thermal spray coating	–	HAp, Nb, HAp/Nb	Mg-Zn-Zr (ZK60)	The corrosion potential was reduced four times. The coatings created a hydrophilic surface	[243]
		Ti, Ta/Ti	Mg-Al-Zn (AZ31)	The coated alloy corrosion improved and due to the passivating nature of the coating, an island-like rough surface was obtained. It facilitates the implant biomineralization process	[244]
		High-velocity oxygen fuel (HVOF) Micro HAp particles	Mg-Al-Zn (AZ61)	The HVOF method produced a surface that presented small cracks. A gradual loss in degradation resistance in the SBF solution was noticed.	[245]

Based on physical methods there can be applied different types of coatings such as ceramic [85, 246–248], polymeric [236, 239, 240], and metallic [244]. Sometimes, pre-treatments must be made to obtain a good adhesion phenomenon between Mg-based substrate and coating.

### Chemical conversion coating

As a result of a chemical or electrochemical reaction, a highly adhesive protective layer is produced on the Mg-based surface, and the obtained coatings are characterized by a low mechanical strength coupled with poor durability under the effect of corrosive media. The method has an advantage because it can lead to a uniform layer formation that can cover even the most complicated geometries. The main techniques used in practice are alkali, acid, electrochemical and hydrothermal treatments.

**Figure 19.**
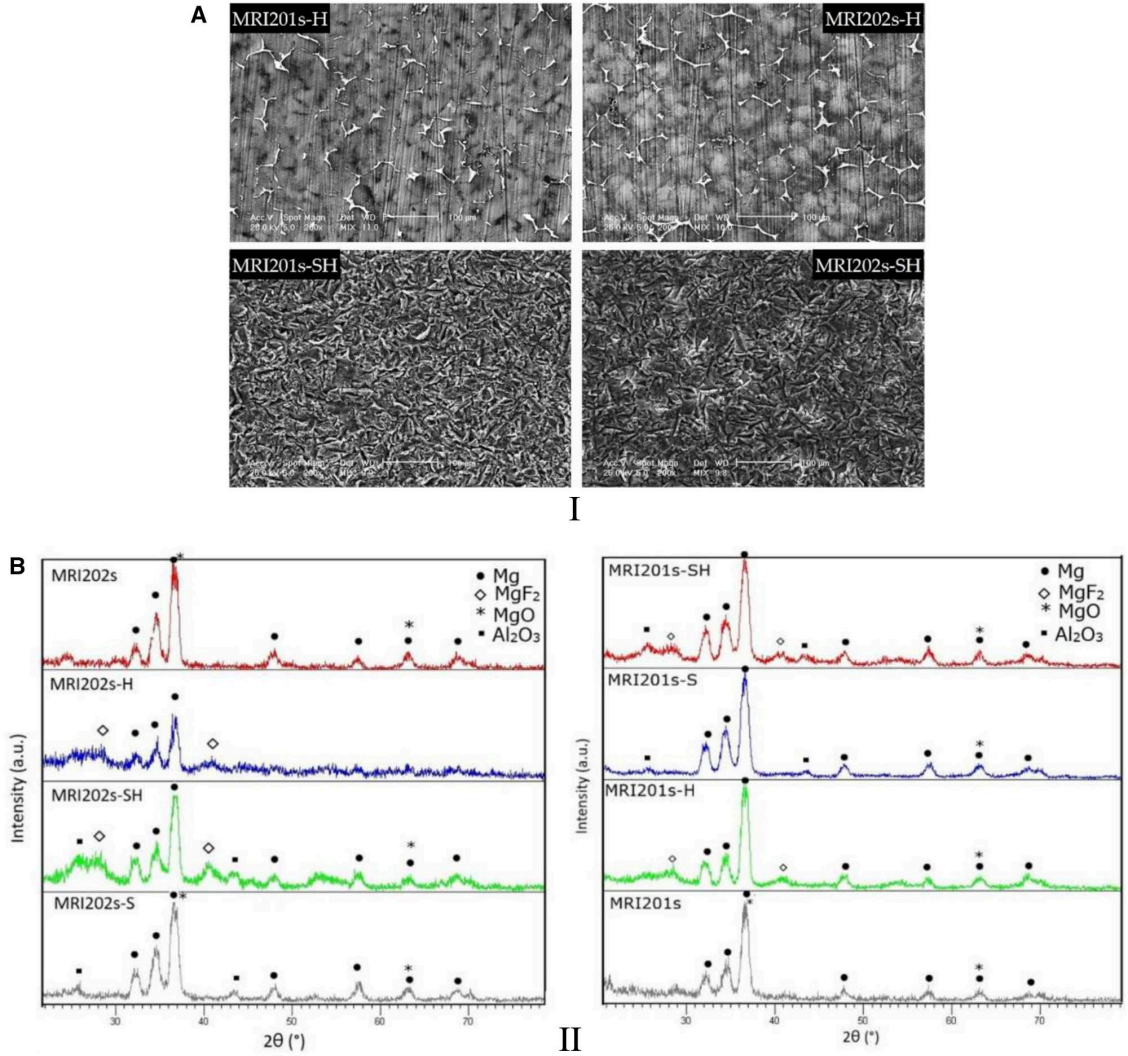
Investigations on Mg-Nd-Y-Zn-Zr alloys. (**I**) SEM micrographs for HF-treated samples (MRI201s-H, MRI202s-H) and sandblasted + HF-treated samples (MRI201s-H, MRI202s-H) in the case of two Mg-Nd-Y-Zn-Zr alloys; (**II**) XRD spectra for untreated and untreated alloy (H—HF treatment, S—sandblasting, SH—sandblasting + HF) in the case of two Mg-Nd-Y-Zn-Zr materials [51]. (Figure is licensed under CC-by 4.0.)

Acid treatment consists of sample introduction in acid solutions. A surface etching effect occurs due to acid action; in this way, the contaminants are removed, and the formation of micro-galvanic cells is prohibited. A thin film forms on the material surface, resulting in degradation rate reduction, passivation and increased implant biocompatibility, favoring cell adhesion and proliferation. Quan *et al.* [51] developed a fluoride conversion coating on Mg-Nd-Y-Zn-Zr alloys and investigated its corrosion properties. The authors chose to apply three surface modifications as follows: treatment with HF acid solution, sandblasting with alumina (Al_2_O_3_) particles and sandblasting combined with acid immersion. SEM coupled with energy X-ray dispersive spectroscopy were used to analyze the surface morphology and elemental composition. The structural characteristics of the samples were investigated based on X-ray diffractometry, and the surface wettability was also checked. By analyzing the SEM images, it was observed that both samples (bare Mg-based alloy and sandblasted material) treated with HF exhibited a smoother surface, and the surface morphology of the substrate was kept (Figure 19 (I)). It was concluded that due to the fact that material grains were visible, the MgF_2_ layer was very thin. The XRD spectra confirmed the presence of the MgF_2_ conversion layer (Figure 19 (II)).

**Figure 20.**
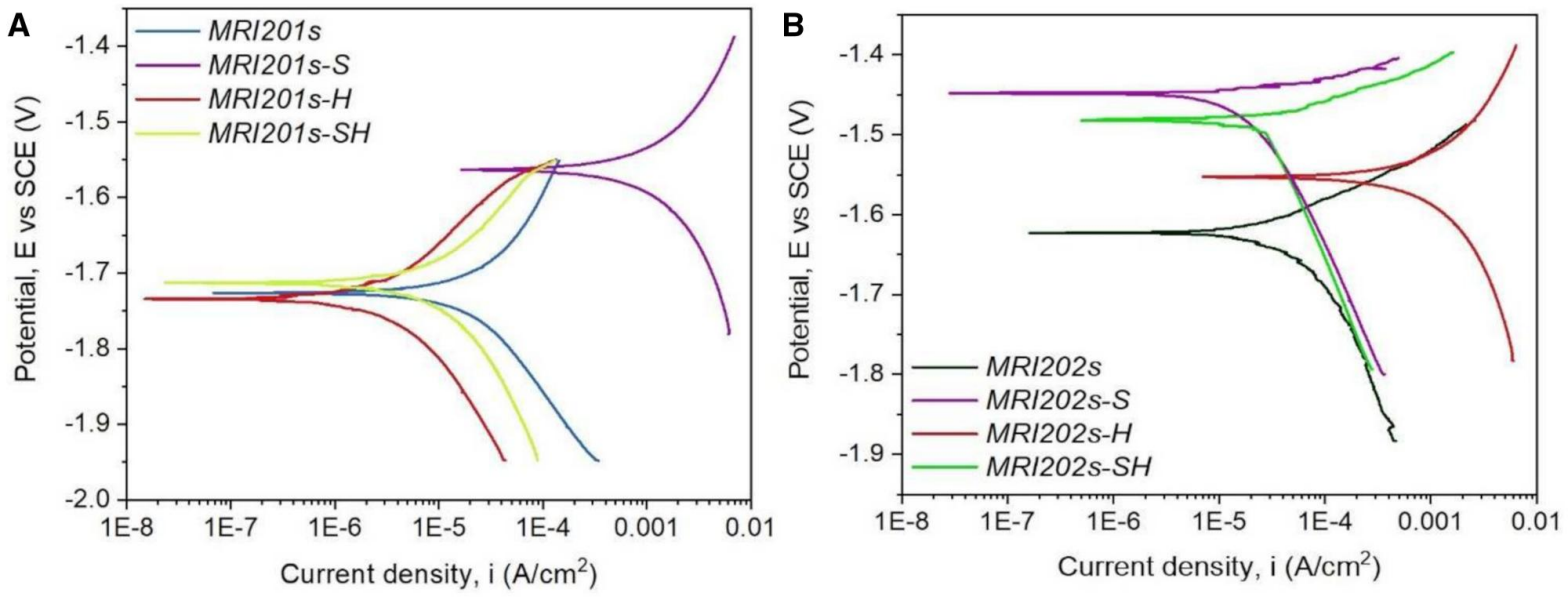
Tafel plots for Mg-Nd-Y-Zn-Zr alloys and the four applied surface treatments. (**A**) MRI201s; (**B**) MRI202s [51]. (Figure is licensed under CC-by 4.0.)

Following the measurements of the contact angles, it was concluded that the HF treatment determined a decrease of this quantity to values of about 20° and was benefic in converting the surface nature to a much more hydrophilic one. The corrosion tests performed in NaCl solution revealed that the acid treatment increased the corrosion resistance almost 10 times higher for the first type of alloy and almost 4 times higher for the second one in comparison with the untreated samples (Figure 20). In conclusion, it was observed that the HF treatment is a suitable method applied to change the surface characteristics, leading to highly biocompatible and anticorrosive implants that can be successfully applied in the orthopedic field.

**Figure 21.**
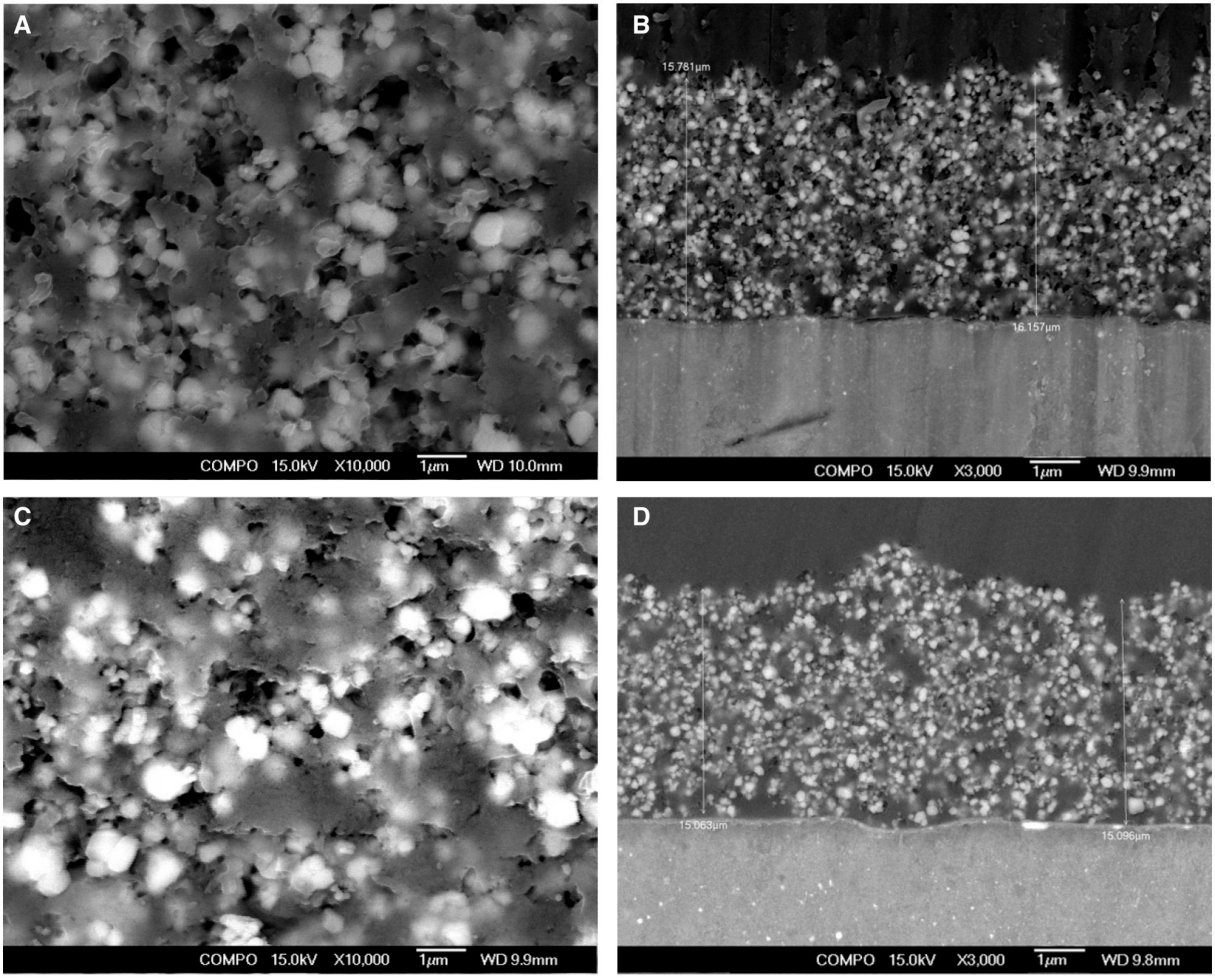
The Hap-coated samples morphology (**A**, **B**) plain and cross-sections views for the first Mg-Zn-Mn alloy and (**C**, **D**) for the second investigated material [82]. (Figure is licensed under CC-by 4.0.)

A subclass of the chemical conversion coatings is the electrochemical conversion, which determines the apparition of a protective film on the substrate surface with the help of an electrochemical reaction. This method is characterized by increased control over the coating thickness and a reduced work temperature. Electrochemical conversion is suitable for samples with intricate geometries. The main techniques used are EPD, anodization, PEO and electrodeposition. Antoniac *et al.* [82] modified the degradation behavior of two Mg-Zn-Mn alloys based on HAp coating made through EPD. For the coating deposition, a laboratory DC power source supplied at 37 V was used, the bare Mg alloys were immersed in HAp solution, and within 5 min, a thin ceramic layer was formed. SEM images showed the formation of a thin HAp layer with a thickness of about 16 μm (cross-section view). Both types of Mg-based alloys exhibited a porous and homogenous surface morphology (Figure 21). The elemental analysis, XRD and FTIR analysis proved the existence of the main chemical elements of the material and HAp. The SEM images taken after the corrosion process of the samples occurred put into evidence the existence of hydroxides and oxides of Mg and Mn. Both coated samples exhibited good corrosion resistance in comparison with the bare alloys. The authors concluded that EPD is an adequate technique for thin HAp layer coatings and can be successfully applied to tune the Mg-based surface properties.

**Figure 22.**
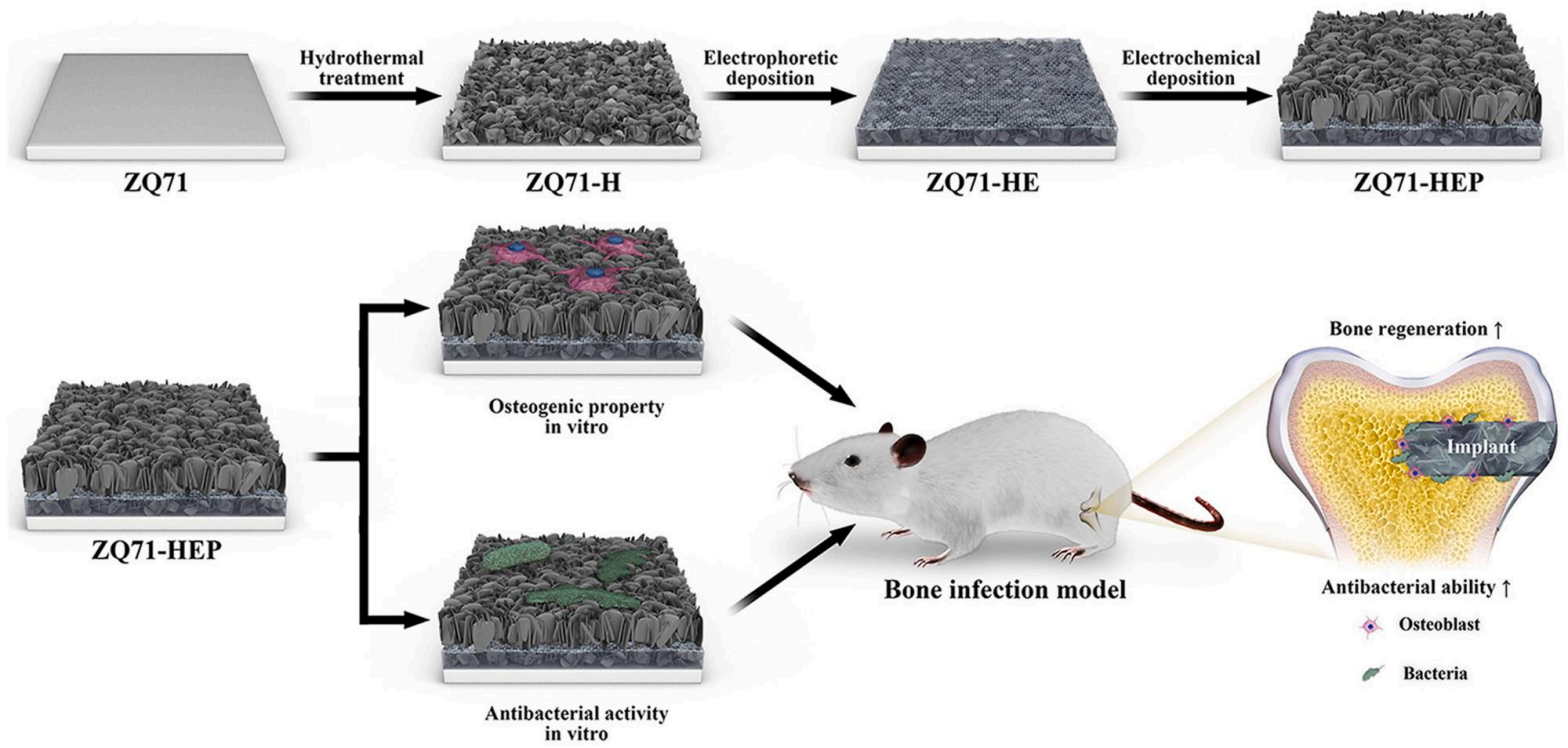
The implant composite coating preparation steps and *in vitro* and *in vivo* tests for biocompatibility and antibacterial properties investigations [249]. (Figure is licensed under CC-by 4.0.)

Hydrothermal treatment and electrodeposition are efficient ways to develop coatings on a metallic substrate. Yuan *et al.* [249] prepared a composite coating consisting of hydroxide/graphene oxide (GO)/HAp on two Mg-Ca-Zn-Ag (ZQ71 and ZQ63) alloys to enhance bone regeneration and increased antibacterial efficiency. They applied a combined strategy comprised hydrothermal treatment, EPD, and electrochemical deposition. Firstly a hydrothermal treatment was used to transform the smooth Mg-based alloy surface into Mg(OH)_2_ coating (ZQ71-H and ZQ63-H), then with the help of EPD, a GO film apparition was induced (ZQ71-HE and ZQ63HE), and by introducing it in the Mg(OH)_2_ coating, nucleation sites for self-assembly HAp nanosheets made from electrochemical deposition method occurred (ZQ71-HEP and ZQ63-HEP). This three-layer coating did not contain cracks, and a strong interfacial bond was put in evidence based on cross-section SEM images. The roughness and the hydrophilicity of the samples were improved (Figure 22).

**Figure 23.**
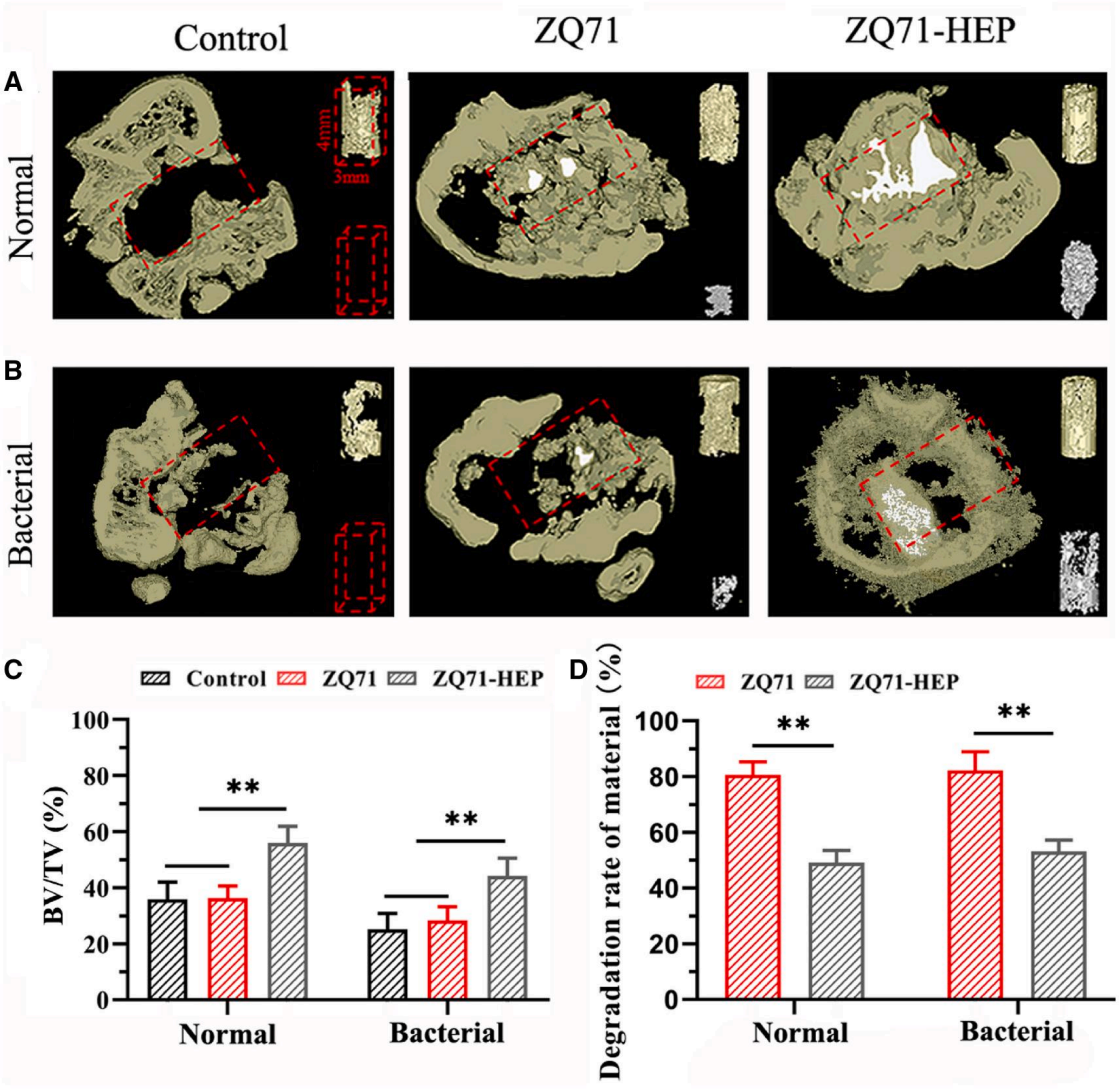
*In vivo* micro-CT evaluations in normal and bacteria-infection conditions. (**A**) 4 weeks postoperative reconstructed image of bone placed in the defect region in normal condition; (**B**) 4 weeks postoperative micro-CT image of bone within the defective region in bacterial conditions; (**C**) Ratio of newly formed volume (BV) versus total volume of the defect area (TV); (**D**) Degradation rate of the material in normal and bacterial conditions (^*^P <* *0.05, ^**^P <* *0.01, ^***^P <* *0.001) [249]. (Figure is licensed under CC-by 4.0.)

**Figure 24.**
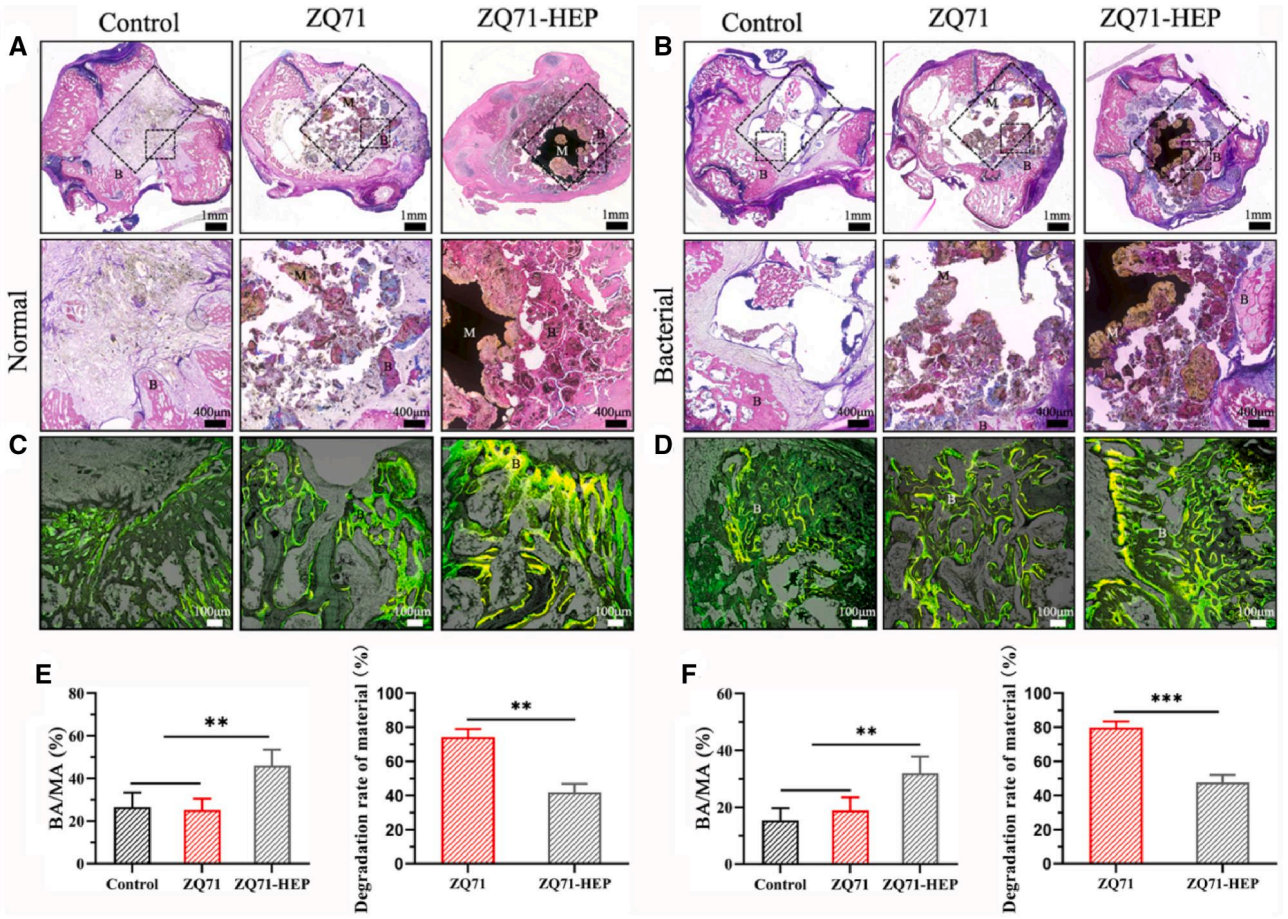
H&E staining of histological sections in ZQ71 alloy. (**A**) Four weeks postoperatively normal conditions; (**B**) 4 weeks postoperatively bacterial conditions; (**C**) *in vivo* new bone formation labeling in normal conditions; (**D**) *in vivo* new bone formation labeling in bacterial conditions (yellow: tetracycline label, green: calcein label); (**E**) new bone substitution and degradation rates of the implant in normal conditions; (**F**) new bone substitution and degradation rates of the implant in bacterial conditions (^*^P <* *0.05, ^**^P <* *0.01, ^***^P <* *0.001) [250]. (Figure is licensed under CC-by 4.0.)

The corrosion tests evidenced an increased corrosion resistance. The implant cytocompatibility was tested using MC3T3-E1 cells, and it was concluded that it promotes the osteogenic cell differentiation process. The *in vitro* investigations showed that the composite coating exhibited good results regarding bone regeneration in healthy animals and in the case of infected ones. Some results are presented in Figures 23 and 24.

Overall, it was noticed that these composite coatings are innovative ones that combine antibacterial with osteoconductive properties and represent a significant step in the implant manufacturing market.

Table 7 summarizes the main chemical conversion coating methods with some literature examples.

**Table 7.** Main chemical conversion coatings with selected literature examples

Technique	Method	Mg-based substrate	Coating	Remarks	Ref.
Electrochemical conversion	Anodization	Mg-Al-Zn (AZ31), Mg-Zn-Zr (ZK60)	MgO, Mg(OH)_2_, MgCO_3_	The time of the anodization process it was varied. Increased corrosion resistance was observed.	[212]
	Electrodeposition	Mg-Zn	Brushite, HAp, fluoridated HAp	A significant improvement in the corrosion resistance of the material was achieved	[250]
		Mg-Al-Zn (AZ21)	Calcium stearate	A dense and homogenous coating was obtained. Due to cracks apparition, a superhydrophobic surface was reported	[251]
	EPD	Mg-Al-Zn (AZ91)	Chitosan (CS)/Bioglass (BG)	The wettability increased with coating concentration. The coating induced high biocompatibility properties on the surface	[252]
		Mg-Y-RE-Zr (WE43)	CS/BG	The coating provided high cytocompatibility of the implant	[253]
	PEO	Mg-Al-Zn (AZ31)	MAO-coating	Improved degradation rate and cell viability	[218]
		Mg-Al-Zn (AZ31B)	MAO pre-treatment followed by sol-gel deposited calcium metaphosphate (CMP) coating	MAO pre-treatment improved the corrosion resistance and reduced the micro-cracks apparition	[254]
Acid treatment	–	Mg-Nd-Zn-Zr	HF treatment -MgF_2_ coating	The corrosion resistance increase.	[255]
		Mg-Ca	H_3_PO_4_ and HNO_3_ treatment Mg_3_(PO_4_)_2_ and Mg(OH)_2_ layers	The coating was considered a stable platform for HAp development.	[256]
Alkali treatment	–	Mg-Ca	Mg(OH)_2_	The degradation behavior improved by a high amount, and toxicity was shown against L-929 cells	[257]
		Mg-Zn-Y-Nd	Mg(OH)_2_	The corrosion rate was reduced due to eliminating the secondary phases on the material surface	[258]
Hydrothermal treatment	–	AZ31-3Ca	CaP	The substrate was covered with a layer with no cracks. Increased corrosion resistance and hydrogen emission was reported	[216]

Conversion coating methods are one of the most accessible technologies to tune the Mg-based alloy surface. After they are applied, an oxide/hydroxide formation on the surface of Mg-based implants is reported. These coatings are characterized by an increased adhesion strength compared to the deposition methods, but they exhibit a main drawback regarding their stability over a long period. One of the most used methods with the best results is the EPD technology. Regarding the other conversion coatings strategies, such as hydrothermal, acid, or alkali treatments, they are involved in Mg-based implant surface tunning only as pre-treatments.

## Recommendations from a translational perspective, challenges and opportunities

Today we can notice an increased demand for bone repair and treatment materials. As mentioned previously, the Mg-based alloys are promising because they are biodegradable and highly biocompatible, and the need for a second surgical intervention is prohibited. Supplementary, Mg exhibits good mechanical properties with values close to those of the cortical bone, so as a direct consequence, it reduces the occurrence of incomplete healing or re-fracturing phenomena by avoiding, in most cases, the stress shielding effect apparition. Many literature studies in our review paper reported the Mg-based alloys' capabilities to stimulate and enhance new bone formation and development and their benefic results on fracture healing management. The translational potential of Mg as the main material for biodegradable implant manufacture is worth mentioning. Shan *et al.* [259] investigated the Mg-based implant potential in promoting bone metabolism. They found that more studies proved that the signaling pathways involved in Mg-mediated bone metabolism included the phosphoinositide 3-kinase (PI3K)-Akt signaling pathway, the RANKL signaling pathway, the TRPM protein pathway, and the Wnt signaling pathway. Regarding the TRPM protein pathway, the intracellular Mg^2+^ concentration is regulated through TRPM6 and TRPM7. The latest signaling pathway upregulates the expression of Runx2 and ALP, generating increased osteoblasts. The authors observed that the main pathways that influenced osteogenic activity are the P13K-Akt and Wnt signaling pathways. The P13K-Akt pathway stimulates the differentiation of MSCs into osteoblasts. Wnt is classified into the Wnt-β-catenin pathway and in a non-classical Wnt signaling. Some research [260, 261] evidenced that Mg ions can generate the β-catenin accumulation in the cytoplasm through proteasomal degradation before the transcriptional activity of the T cell factor-lymphatic enhancer factor that helps the osteoblastic differentiation. Mg ions play a vital role in osteoclast activity through the OPG-RANK-RANKL signaling pathway. A Mg deficit is usually correlated with bone resorption by influencing the serum levels of RANKL and OPG [262, 263]. In addition, Mg has an important effect on angiogenesis by increasing the expression level of the VEGF and the endothelial nitric oxide synthase [264]. It can be observed that when a certain Mg concentration is obtained, the P13K-Akt signaling pathway is activated, and osteogenic differentiation is promoted. Osteoclast differentiation is controlled by M-CSF and RANK, which sustain the coexistence of bone resorption and formation processes during the early stages of Mg alloy implantation. It can be observed from this study that Mg-based implants are an excellent candidate for fracture treatment and bone reconstruction process, playing an important role in bone remodeling, as explained above. However, much more research must be performed to establish and understand exactly how Mg ions act during osteogenesis and angiogenesis phenomena.

All cytotoxicity testing based on ISO 10993 Parts 5 and 12 [265, 266], which describe the main steps used for the biocompatibility assessment of medical implants and devices, could not be directly applied to prescreening toxic evaluation of Mg-based alloy [267]. ISO 10993-5 stipulates that cytotoxicity should be measured through cell death, cell development and proliferation inhibition and colony-forming ability [265]. Also, the extracts must be prepared and incubated for a certain time in the extraction medium following ISO 10993-12 at a constant humidified temperature of 37°C and 5% CO_2_ [266]. The direct cytotoxicity tests consist of direct exposure of the cells to the samples, while the indirect tests are characterized by specimen immersion. The control parameters that are considered in classical cytotoxicity tests for *in vitro* investigations are extraction time, extraction medium and extraction volume-to-surface ratio [268]. Initially, these testing standards were developed for non-degradable materials, which makes them problematic when applied to degradable alloys. In the case of non-degradable materials, it is well-known that only a few ions can be released and accumulated into the extracts during the recommended 72 h of immersion. The biosafety analysis strongly depends on the chemical composition of the material because some elements can be toxic even at a trace level. Biodegradable Mg-based alloys react with water, generating increased Mg ions release and high pH and osmolality values in the surrounding medium [269]. The indirect cytotoxicity test is considered more appropriate for Mg-based alloys, and a typical 3 h extraction time is recommended [268]. Fisher *et al.* [270] investigated these concerns and related them to extract preparation, which in some cases can be linked to cell death for *in vitro* investigation due to osmotic shock apparition. Supplementary, the *in vitro* test character was considered static, which can differ from the dynamic behavior of degradable materials inside the human body. Other drawbacks, such as strong hydrogen emission and the apparition of different corrosion products, were analyzed by several groups [267, 271, 272], and concluded that they must be carefully addressed by taking into consideration that inside the human body, these changes are regulated by the local environment. They proposed the use of a bioreactor as a solution. In cases where this flow system is unavailable, a dilution of the extract must be applied. Fischer *et al.* [270] concluded that protein inclusion in the corrosion medium, establishment of the right concentration range, cell culture conditions, and types of cells, such as primary ones collected directly from patients, are important factors that must be considered when biocompatibility of new magnesium-based alloys is investigated. The authors recommended diluting the extracts 10 times to control the extracellular osmolality (lower than 400 mOsmol/kg). Starting from previous analyses, Wang *et al.* [267] noticed that a 6–10 times dilution process of the extracts for *in vitro* investigations must be applied and considered for inclusion in the current ISO standards. Different protocols were presented in the literature, and an overall conclusion that can be foreseen is the fact that an increased concentration of ${\text{HCO}}_{3}^{-}$and albumin can determine interactions with the Mg byproducts and can interfere with classical *in vitro* protocols [92, 273]. Fischer *et al.* [274] showed that corroded Mg transformed tetrazolium salts to formazan, being linked to false cell viability results. The principle of tetrazolium-based tests such as XTT (2,3-bis(2-methoxy-4-nitro-5-sulfophenyl)-5-((phenylamino)carbonyl-2H-tetrazolium hydroxide)) and MTT (3-(4,5-dimethyl-2-thiazolyl)-2,5-diphenyl-2H-tetrazolium bromide) consists of conversion of the yellow-colored tetrazolium salt based on metabolic action of living cells into an orange (XTT) or blue (MTT) formazan dye. One of the most used cell lines involved in the XTT/MTT test is mouse fibroblastic cells (L929) [275]. The main conclusion of the study was that XTT and MTT tests can lead to false negative or false positive results. The authors did not exclude the importance of such investigations but revealed that many controls must be included to analyze the influencing parameters and obtain approximately real results. They stated that tetrazolium-based tests must be carefully used and, when possible, replaced with other investigations, such as BrdU assays, which proved to be not influenced by the corrosion products of Mg-based alloys. Gai *et al.* [276] developed a novel method to evaluate the dynamic biocompatibility of degradable biomaterials, such as Mg-based alloys, based on real-time cell analysis (RTCA). They made measurements of Mg^2+^ concentration and osmolality based on ISO 10993:12 on extracts immersed in different media and investigated the material biocompatibility using MTT assay and xCELLigence RTCA on mouse fibroblastic cells (L929), human osteosarcoma cells (MG-63), and human umbilical vein endothelial cells. The RTCA is adequate for dynamic cellular tests by providing a high accuracy comparable with conventional cellular assays. The results of the MTT assay evidenced an inhibition rate of about 80% in the case of tested cell lines for Mg-Ca alloys since, in the case of pure Mg extracts with a similar concentration, a value of 40% was obtained. The RTCA test showed different dynamic modes for the cytotoxic process in a direct relationship with the tested cell line, dilution rate of the extract, and Mg-based alloy chemical composition. It was concluded that RTCA is a suitable tool for the cytotoxic evaluation of degradable metals, but much more research is needed on a wider range of biomaterials to develop a methodology for this method in order to establish a future emendation of ISO standards. Jung *et al.* [271] identified a test scheme that included an indirect assessment of soluble Mg corrosion products’ influence on material extracts based on BrdU-assay, XTT-assay, and LDH-assay and a direct assessment of the surface compatibility by considering the cell attachment, proliferation, and viability through Live-dead staining, direct BrdU-, XTT- and LDH assays. These tests were applied for Mg-based alloys, and cytotoxicity with reduced cell viability (<75%) was reported in the case of *in vitro* studies compared to the *in vivo* results [277]. A possible explanation is that the OH^-^ and Mg^2+^ ions are dissolved faster inside the human body due to Cl^-^ ion presence and then eliminated based on different routes outside the body. Thus, following the previous studies, a dilution step of Mg alloy extract before the cytotoxicity investigations on different cell lines is recommended. Today, the 10 mM level of Mg^2+^ ions found in the diluted extracts is considered a safe limit for cell viability higher than 75% [267]. In addition, tetrazolium-based tests should be used carefully and, in some cases, in which the cytotoxicity analysis is considered of utmost importance, they can be replaced with other types of assays, as explained above, due to interaction between corrosion products of Mg and tetrazolium salt.

*In vivo* investigations are essential to study the safety and effectiveness of an implant prior to its introduction in clinical trials. Usually, small animal models such as rats, rabbits, or mice are used for biosafety evaluations, and drawbacks such as system toxicity or skin irritation are observed in good accordance with the ISO standards [278–281]. The bioefficacy of the implants is analyzed based on large animal models such as goats, sheep, dogs, or pigs. Some animal studies are presented in Tables 1–3 and are widely described in the article in a direct relationship with the material properties. Unfortunately, many studies found in the literature did not correlate the place of implantation in animals with a corresponding zone in the human body. There are no surgical recommendations that can be followed in a possible clinical trial, and some discrepancies regarding the effect of the treatment between animal models and humans can be foreseen. Some trauma-related implants have already been approved for use in humans, and today, different fracture treatments can be applied (Table 2). Examples are provided in Design and examples section in the case of different screw and pin implants. Actual and future studies must facilitate a transition from simple application to practical problem-solving. In this direction, Zhu *et al.* [282] noticed that biodegradable magnesium implants can alleviate *the medication-related osteonecrosis of the jaw* (MRONJ) in rats, concomitantly enhancing the angiogenesis. The authors explained that MRONJ is a complication localized in the jaw, and it can be induced by antiangiogenic and antiresorptive drugs, which deteriorate the angiogenesis process. The *in vivo* studies were performed on six groups of 50 SD rats that were treated as follows: the first group of 5 rats with Ti implant (Veh + Ti), the second group of 15 rats with bisphosphonate (BP) and Ti implant (BP + Ti), the third group of 15 rats with BP and Mg implant (BP + Mg), the fourth group of 5 rats with BP, Mg implant and VEGF receptor-2 inhibitor (BP + Mg + SU5416), the fifth group of 5 rats with BP, Mg implant and CGRP receptor antagonist (BP + Mg + BIBN) and the sixth group of 5 rats with BF, Mg implant, VEGF receptor-2 inhibitor and CGRP receptor antagonist (BP + Mg + SU5416 + BIBN). New bone formation and blood vessel apparition were investigated using immunohistochemistry, histomorphometry and micro-CT analysis methods. They noticed 8 weeks after surgery that the third group exhibited a low occurrence of MRONJ-like lesions and osteonecrosis, with high bone microstructural parameters and increased expression of CGRP and VEGF compared to the second group. In addition, by blocking the VEGF receptor-2 and CGRP receptor, the new bone formation and blood vessel apparition were significantly reduced for the fourth, fifth and sixth groups, raising the possibility of MRONJ-like lesion development, and increasing the risk of bone necrosis. The main conclusion of this study was that Mg ions can upregulate the VEGF- and CGRP-mediated angiogenesis and, in this way, alleviate the MRONJ lesion. This study evidenced that Mg implants have a great translational potential to be used for internal fixation in the case of patients with a high risk of MRONJ. Other studies evidenced that Mg proved to be efficient in the case of osteoporotic and refractory bone defects patients. Lin *et al.* [283] mixed poly(methyl methacrylate) cement with Mg particles and observed increased angiogenic and osteogenic properties. Mg powder was involved in composite bioactive scaffold preparation and proved an efficient solution for critical-size bone defects [284]. Although there are many studies regarding Mg-based implants, it is still a challenge to translate them to clinical applications because the Mg ion metabolism still needs to be clarified, and further investigations are necessary. Also, there is no standard procedure for the biological evaluation of the Mg-based implants, as presented in this section, and their translation from bench to bedside is still limited. We can conclude that Mg implants have an important potential for bone regeneration, and increasing the research in this direction is of utmost importance.

A main challenge in Mg-based implant manufacture is their accelerated corrosion rate in physiological media. In order to address this limitation, surface modifications and coatings or alloying with different chemical elements can be applied. The coating procedure must be carefully done because if it is not properly conducted, an increased corrosion rate is reported, followed by implant loosening or an inflammatory reaction due to hydrogen release can occur. The coatings have to be characterized by increased biocompatibility to hinder side effects such as osteolysis. Regarding the alloying procedure of Mg-based implants, elements such as RE or aluminum are extensively used to decrease the material corrosion rate. However, the neurotoxic effect of Al or the toxicity and foreign body reactions caused due to RE addition must be further investigated. An important challenge in the Mg-based implant industry consists of optimizing their chemical composition and finding a proper coating that permits uniform substrate corrosion. Supplementary, the implant surface must provide healthy cell development and a good adhesion environment.

Innovative coatings functionalized with drugs can be developed as future opportunities because it is well-known that localized treatments are more effective. Multilayered composite or hybrid inorganic/organic coatings can be developed in this direction. For example, the outer layer can be polymeric and incorporate the drugs, and the inner one can be made from ceramic materials because a better adhesion is present at the metallic-ceramic interfaces. Dong *et al.* [285] developed poly (1,3-trimethylene carbonate) (PTMC) and polydopamine (PDA) bilayer and micro-arc oxidation composite coatings for pure Mg alloys applied in two steps. The first consisted of a micro-arc oxidation procedure, and the second comprised sample immersion. The authors noticed that by combining the PTMC and PDA coatings, the sample corrosion resistance was improved and stated that this type of innovative coating can be loaded with drugs in the future to treat different orthopedic diseases. The ultrasonic micro-arc oxidation procedure was selected to increase the adhesion forces between the substrate and coatings, and a MgO ceramic layer was obtained. After that, the combined PTMC-PDA layer sealed the holes of the MgO layer and attached very well to it due to the increased surface roughness. The *in vitro* experiments showed that the composite coating provided good corrosion protection and high biocompatibility. The animal studies proved that the coated samples had a slow degradation inside the body, and the coatings did not exhibit any toxic activities against animal viscera. It was concluded that the proposed inorganic/organic coating applied on a pure Mg surface could be successfully used in a translational perspective for degradable orthopedic Mg implants. Chen *et al.* [286] proposed an innovative fabrication route of a zoledronate-incorporated coating on magnesium alloys to be used as orthopedic implants. The translational potential of this study consisted of drug release mode, based on a hydrogen bond between medicine carriers and drug molecules that permitted a continuous administration of the active substances. The coating was obtained after a preliminary fluoride pretreatment and a dip coating procedure in chitosan-acetic acid and ZA mixed solutions. Li *et al.* [287] coated high-purity Mg with Ca-deficient hydroxyapatite (CDHA) based on the hydrothermal method, and then polydopamine (DOPA) was involved in immobilizing VEGF on the CDHA layer. The authors found that the composite coatings increased the corrosion resistance of the Mg implants and showed very good biocompatibility with a high proliferation rate and adhesion of MC3T3-E1 cells. Based on immunofluorescence and quantitative real-time PCR measurements, it was noticed that VEGF that was immobilized on Mg promoted the differentiation of MSCs into endothelial cells. This study showed a translational potential for large bone defect treatment, for which blood vessel regeneration is important to ensure oxygen and nutrient circulation.

Another future direction can be considered the development of self-reporting coatings against corrosion or coatings that include fluorescent indicators. These indicators can report when the implant has a defect, making its visualization possible. Some studies reported the importance of a self-healing capability of the coating to announce when the release of Mg^2+^ ions is higher than the permitted biological range or to underline an increase of the pH in the surrounding environment of the implant. Xiong *et al.* [288] made a pH stimuli-responsive self-healing coating applied onto Mg-Ca alloy. This complex layer was a sandwich comprised fluoride precoating, silk-phytic acid, and silk fibroin. The silk-phytic acid was loaded into the median layer and exhibited inhibitory activity by dissolving the Mg^2+^ and Ca^2+^ ions. On the other hand, the phytic acid had self-healing capabilities simultaneously with a pH-responsive property. This innovative coating proved to exhibit increased corrosion resistance and excellent biocompatibility. Li *et al.* [289] designed a self-repairing coating with immunomodulatory functions on Mg. They encapsulated curcumin and released F^-^ ions into mesoporous silica capsules. This hybrid coating consisted of three layers: an inner MgO layer, a poly-l-lactide layer, which intercalated F^−^encapsulated mesoporous silica nanocapsules loaded with curcumin, and a brushite outer layer. It was noticed that F^−^ ions made the self-healing function since the bone immunomodulatory effect was sustained by curcumin. The authors concluded that through a high concentration of curcumin, it is possible to achieve BMMSC differentiation into osteoblasts concomitantly with enhanced bone integration due to contact osteogenesis. All these applications exhibited a high translational potential for bone-critical size defects by guiding stem cell differentiation and maintaining a permanent control of the environmental factors in the defect neighborhood.

Our study’s main conclusion is that adapted interfaces for biodegradable Mg-based alloys are an actual topic, and much more research must be done in order to adapt the Mg-based alloys’ corrosion rate and modify its surface to obtain better implant integration and increased osteogenesis, according to the clinical needs for specific pathology. Innovative scaffolds can be developed to treat medium and large bone defects and conventional treatments can be combined with advanced ones. Although, as seen from our article, there are many medical challenges in a direct relationship with Mg-based implants, the research in this field is moving fast, and many of them are solved step by step. We can identify the need for a standardized system for *in vitro* and *in vivo* analyses to compare the large variety of results found in the literature. Even though many results appear, a need for biofunctional testing of specific orthopedic implants made by Mg-based alloys on animal models was identified. In this way, Mg alloy implants and scaffolds will be used in clinical practice as soon as possible to alleviate the existing orthopedic problems or offer a viable treatment solution for critical-size bone defects.
